# Supplementary material for: Analysis and reporting of adverse events in randomised controlled trials: a review
Source: BMJ Open. 2019 Mar 1;9(2):e024537. doi: 10.1136/bmjopen-2018-024537 (PMC6398660; doi:10.1136/bmjopen-2018-024537)
Supplement: Supplementary data [file bmjopen-2018-024537supp001.pdf]

Supplementary material

Table A1: Data items extracted from publications

			Items collected	Instructions
**Study details**		1	Study number	
		2	Journal	
		3	Funding source: public, private, both or unspecified.	Studies will be assumed to be funded by industry only if this is explicitly stated.
**Study characteristics**		4	Control: placebo, active or both	Select placebo if no active treatment is given, else active. Both should be selected for trials with multiple arms where there is at least one group receiving no active treatment and one group receiving an active treatment.
		5	Number of centres	
		6	Number randomised	
		7	Study duration (length of trial follow-up)	
**Methods**	Details of how AE outcomes were defined (coding, attribution) and were collected (mode of collection, timing)	8	Describe the collection method: passive surveillance, patient prompted, clinical examinations (e.g. vital signs or urine samples), and laboratory tests. (Select all that apply)	*Passive*: If authors state that AEs were collected throughout the study with no further information we will assume that collection was passive.<br>*Prompted*: Prompted methods include, but are not limited to: questions about both specific events and AEs in general, questionnaires, or diaries.
		9	Stated the timing of collection.	
		10	Mention dictionary for coding of events: Researcher defined, MedDRA, CTCAE, WHO-ART, COSTART, ICD-10, other or not applicable	
		11	Describe who undertook the assessment of attribution to study drug: blinded assessor, unblinded assessor or not specified.	
**Planned analysis**	Details of any plans for analysing AE outcomes	12	Describe analysis for AE outcomes in the statistical methods.	Reference must be made to harmful events e.g. AEs or a specific harm event, this cannot be simply how binary events will be analysed.
		13	Define a 'safety' population for analysis.	
		14	Specify a planned interim analysis with stopping criteria: based on efficacy, based on safety, based on both efficacy and safety, yes but no other details given, no planned interim analysis or unclear	Criteria for stopping must be set out, it is not enough to say that the DMC reviewed the data.

**Results**	Details of what was reported and where	15	What was reported in the main paper: summaries of type of AEs (e.g. AE, SAE, AR, ADR), actual AE terms, both, neither or not applicable?	Not applicable is relevant when for example authors explicitly state that there are no events or there is only one event so summaries are inappropriate.
		16	What was reported in the appendix: summaries of type of AEs (e.g. AE, SAE, AR, ADR), actual AE terms, both, neither or not applicable?	Not applicable is relevant when for example authors explicitly state that there are no events or there is only one event so summaries are inappropriate.<br><br>We will only search the appendix/supplementary material for AE data if the main article makes reference to it.
		17	Who was the AE analysis performed on: all randomised, participants who took at least a single dose, other or not specified?	
		18	How were number of drop-outs/withdrawals reported: By treatment arm, overall, not reported or not applicable?	Not applicable is relevant when there are no drop-outs/withdrawals.<br><br>This does not include discontinuation of treatment.
		19	Were drop-outs/withdrawals due to AEs reported: Yes, no or not applicable?	Not applicable if drop-outs/withdrawals are not reported or if it is reported that there are no drop-outs/withdrawals.
		20	Were specific AEs causing withdrawals reported: Yes, no or not applicable?	Not applicable if drop-outs/withdrawals due to AEs are not reported or if it is reported that there are no drop-outs/withdrawals due to AEs.
		21	What was the selection criteria for the AEs reported?	Free text response where possibilities can include for example**:** most frequent, above a severity threshold, SAEs.<br><br>Include details of what's in the main journal article and what is in the appendix separately.
	Details of how AEs were summarised and presented - binary outcomes	22	What summary information was given: Number of people, number of events, both, unclear, not summarised or not applicable?	Only select 'number of events' if presented for each individual event not just overall number of events.<br><br>Not applicable is only relevant when report that there are no AEs.
		23	What analysis was performed: frequencies, percentages, differences and 95% confidence intervals, significance tests, other? (Select all that apply)	

	Details of how AEs were summarised and presented - continuous outcomes	24	Were continuous outcomes dichotomised: Yes for all, yes for some, no or not applicable?	This includes measures that will have been captured as continuous and then dichotomised for example blood levels, blood pressure etc.
		25	If continuous outcomes were analysed as continuous what analysis was performed: differences in measures of central tendency, significance tests, other? (Select all that apply)	
	Details of how AEs were summarised and presented	26	Were signal detection methods used?	
		27	Were any graphical summaries of AEs presented?	
		28	Were severity ratings given: Yes for all, yes for some, no or not applicable?	
		29	Were numbers of serious events presented: Yes by treatment arm, yes overall, no or not applicable?	If death is reported as part of the efficacy outcome it is not enough to constitute reporting serious events.
		30	Were serious events coded as treatment related: Yes for all, yes for some, no or not applicable?	
		31	Provided information on the duration of events?	This refers to the length of the actual AE i.e. how long did it last.
		32	Provided information on the timing of events?	This refers to the time of onset of the AE.
		33	Accounted for multiplicity of statistical tests?	
		34	Referenced CONSORT extension for harms?	

Table A2: Rationale for items extracted

Item	Rationale
How AE data was collected (mode of collection, timing) and defined (coding, attribution) during the study.	Variation in the collection and definition of events could explain differences in the incidence of observed events.[13, 14] For example specifically asking participants about an event of interest in one treatment group whilst relying on patient report in another is likely to lead to a disparity in incidence of events unlikely to be related to the medicinal product.
Assessment practices of severity of the event or relatedness to the medicinal product.	Attribution of causality by an unblinded assessor allows for subjectivity and bias (even if subconscious) to enter into their decision which can have important implications on the risk-benefit assessment.
Planned AE analysis (final and interim monitoring plans and analysis populations).	For example, the intention-to-treat population is likely to underestimate the AE risk by inflating the denominator. Therefore, this needs to be considered when making conclusions about a drug's safety profile.
How events were selected for inclusion in the journal article.	Due to the space constraints in journal articles it is not always feasible to report all AEs experienced by participants. Therefore, articles often only report a subset of AEs and how these are selected for inclusion has important implications for the safety evaluation. Arbitrary selection criteria can lead to inconsistencies in what is presented across trials for the same disease and/or drug. This prevents an accurate overview of the AEs experienced and invalidates any potential systematic review of events.
How and what summary event information was presented in the journal article.	For example, the number of events and duration of events provides insight into the impact of AEs, with repeated or longer events potentially having far wider clinical implications than a single, shorter event for both patients and prescribers.
How AEs were analysed.	There are many challenges to be considered when analysing AEs in clinical trials. For example, inappropriate statistical testing can lead to misleading conclusions e.g. failure to find a statistically significant result leading authors to conclude that the medicinal product is safe or chance imbalance could lead the authors to erroneously stopping a trial too early.[3-6]

Table A3: Examples of reporting practice in reviewed articles

Example no.	Study	Example practice	Example
1	Litonjua et al.[20]	Description of AE collection method	*"Study staff met with pregnant women monthly to administer a brief health questionnaire, assess medication use, and monitor for complications (via the questionnaire and medical record review)… After delivery, children were monitored by telephone every 3 months and in-person annually for 3 years, during which time infants' health, respiratory symptoms, and medications were assessed"*
2	Miller et al.[21]	Description of AE collection method	*"Safety evaluations included physical examinations, assessment of vital signs, clinical laboratory tests, and reporting of adverse events at each study visit"*
3	Libman et al.[22]	Description of planned AE analysis	*"The proportions of participants experiencing any adverse event, any related adverse event, any gastrointestinal event, any event other than a gastrointestinal event, at least 1 severe hypoglycaemic event, and at least 1 diabetic ketoacidosis event in each treatment group were compared using the Fisher exact test. The number of adverse events, new adverse events, serious adverse events, and non-serious adverse events were compared between groups using a Wilcoxon rank sumtest."*
4	Gross et al.[23]	Description of planned AE analysis	*"Safety analyses and secondary efficacy analyses used binomial regression, analysis of covariance, or the marginal Cox proportional hazards model as appropriate"*

Table A4: Stopping criteria for safety

Study	Main article text	Appendix text
Myles et al.[24]	"O'Brien–Fleming stopping boundaries were used to assess efficacy, and a less stringent boundary was used to assess harm."	
Billings et al.[25]	"The data and safety monitoring board (DSMB) reviewed patient recruitment practices, safety reporting, and data quality after 30 patients completed the study; performed an interim analysis after 277 patients … had completed the study to assess *safety of the intervention*; and performed a second interim analysis after 546 patients … had completed the study to assess the safety, efficacy, and futility of the intervention. The DSMB made recommendations based on qualitative assessments of the safety, efficacy, and futility of the intervention…"	"*Suspend enrolment in any study arm … due to safety concerns based on study intervention. Safety concerns include*:<br>• Increase in in-hospital all-cause mortality in subjects randomized to A or B such that the DSMB deems the increase is excessive compared to A or B.<br>• Increased treatment toxicity in either treatment group deemed excessive. Toxicity is defined as moderate or severe myalgias.<br>• Increased severity of adverse events deemed "Probably Related" or "Possibly Related" to study intervention in either treatment group. Itemized adverse event reports separated by treatment will be provided.<br>• Increased AKI incidence in either treatment group deemed excessive. • Increased incidence of stroke or hemodialysis requirement in either group (secondary endpoints) deemed excessive."
Beardsley et al.[26]	"An independent data and safety monitoring committee oversaw trial safety and analyzed unblinded data after every 50 deaths, according to its charter ..."	"The Haybittle-Peto boundary, requiring $p<0.001$ at interim analysis to consider stopping for efficacy, will be used as guidance. *A level of significance of 1% will be used as a guide for stopping the trial early because of a detected harm of dexamethasone*. In addition, the DMEC will receive conditional power curves to assess whether it remains realistic that the trial will demonstrate superiority of dexamethasone conditional on the data accrued up to the point of the interim analysis. Importantly, the DMEC recommendations will not be based purely on statistical tables but will also use clinical judgment."
Kor et al.[27]	"In addition to statistical criteria for significance, the study included a priori "go-no-go" definitions for recommending continuation to phase 3 study ... Briefly, continuation to phase 3 would occur with a positive primary outcome finding along with an acceptable safety profile. An acceptable safety profile was defined as a serious adverse event profile for aspirin that was not statistically worse than placebo (95% CI for the relative risk of any serious adverse event covers the null value of relative risk = 1.0). The "no-go decision" was defined as early termination by the data and safety monitoring board for safety or unfavorable risk/benefit ratio. An indeterminate case in which there was a non–statistically significant effect but this effect was in a clinically meaningful direction was also defined."	Initiate Phase III Study: Demonstrated efficacy signal in addition to adequate safety profile Criteria: Early termination for benefit at interim analysis or $p<0.08885$ at final analysis (alpha=0.10 for study). *Serious adverse event profile of ASA not statistically worse than placebo (95% confidence interval for the relative risk of any SAE covers the null value of RR=1.0).*<br>Further Development Potentially Required: Weak efficacy signal Criteria: Primary endpoint did not achieve a priori level of significance but there were at least a general consistency of secondary endpoints indicating propensity for efficacy with a larger sample size and/or more specific primary endpoint.<br>Abandon Treatment Platform: Harm (in efficacy or safety endpoints) Criteria: Study terminated early per recommendation by DSMB for safety and/or risk/benefit ratio concerns (i.e., stop for futility, harm, unacceptable risk profile, etc.)

Nichol et al.[28]	We used a group sequential statistical approach to do two equally spaced pre-planned interim analyses (at 33% and 67% of total recruitment) to assess accumulated safety data (differential proportions of deep venous thrombosis and total mortality). This *approach was chosen to provide for early stopping for probable harm* or strong evidence of benefit. We applied the Haybittle-Peto criterion ($\lvert Zk \rvert \geq 3$) for early stopping at these analyses.	

Table A5: Selection criteria used to select AEs presented in the main journal report

Selection criteria	n	%
All AEs presented	20	10.87
AEs in greater than x% in any group	10	5.43
AEs in greater than x% in treatment group	4	2.17
AEs in greater than x% in all patients	1	0.54
Most common (no criteria specified)	9	4.89
Predefined AEs	26	14.13
SAEs	15	8.15
AEs leading to study drug discontinuation/interruption	3	1.63
Treatment related AEs	5	2.72
Grade 3>= events	9	4.89
AEs in greater than x% in any group & predefined/special interest AEs	4	2.17
AEs in greater than x% in any group & frequency between groups differed by more than y% & predefined/special interest AEs	1	0.54
AEs in greater than x% in all patients & predefined/special interest AEs	3	1.63
AEs in greater than x% in treatment group & AEs of special interest	2	1.09
AEs in greater than x% in any group & all SAEs	2	1.09
AEs in greater than x% in all patients & all SAEs	1	0.54
AEs in greater than x% in any group & SAEs related to treatment	1	0.54
Most common (no criteria specified) & predefined/special interest AEs	3	1.63
Most common (no criteria specified) & all SAEs	4	2.17
Most common (no criteria specified) & all SAEs & AEs leading to study drug discontinuation/interruption	1	0.54
Most common (no criteria specified) & treatment related SAEs	1	0.54
AEs where frequency between groups differed by more than y% & all SAEs	1	0.54
AEs of special interest	6	3.26
Grade >=3 AEs in greater than x% of patients	1	0.54
Grade >=3 AEs in greater than x% in intervention & y% in control	1	0.54
Most common (no criteria specified) grade 3>= AEs	1	0.54
Most common SAEs (no criteria specified)	1	0.54
SAEs & AE of special interest	1	0.54
Treatment related AEs in greater than x% of patients	1	0.54
Treatment related AEs in greater than x% in any group	1	0.54
AEs in greater than x% in treatment group & SAEs	1	0.54
AEs in greater than x% in treatment group & SAEs & predefined AEs	2	1.09
AEs in greater than x% in any group & significantly different & SAEs	1	0.54
AEs in greater than x% in any group & treatment related AEs/SAEs	2	1.09
AEs in greater than x% in treatment group & treatment related AEs & SAEs	1	0.54
AEs in greater than x% in treatment group & treatment related AEs in greater than y% in all patients	1	0.54
AEs in greater than x% in any group & Grade 3>= events	1	0.54
AEs in greater than x% in all patients & Grade 3>= events	1	0.54
AEs in greater than x% in all patients & Grade 2>= treatment related AEs	1	0.54
AEs in greater than x% in any group & Grade 3>= events in greater than y% in any group	1	0.54
AEs in greater than x% in any group & SAEs in treatment group	1	0.54
AEs in greater than x% in any group & AEs of special interest & most common (no criteria specified) AEs leading to treatment discontinuation/interruption & predefined AEs	1	0.54
AEs in greater than x% in any group, AEs of special interest in greater than y% in treatment group & treatment related deaths	1	0.54

AEs in greater than x% in treatment group & SAEs in greater than y% in any group	1	0.54
AEs and SAEs occurring more often in treatment group than control	1	0.54
AEs in greater than x% in treatment group & occurred more often in treatment group than control & predefined/special interest AEs	1	0.54
AEs in greater than x% in any group & frequency between groups differed by more than y%, SAEs in greater than z% in any group & all grade >=3 AEs	1	0.54
AEs in greater than x% patients & more than y% difference between treatment groups & AEs leading to treatment discontinuation/interruption & most common SAEs (no criteria specified) & death	1	0.54
Predefined AEs, AEs leading to hospitalisation/death/study drug discontinuation/interruption & SUSARS	2	1.09
Some form of overall summary	6	3.26
Not specified how selected	6	3.26
Not summarised in main paper	11	5.98

Table A6: Selection criteria used to select AEs presented in the appendix

Selection criteria	n	%
All AEs	18	9.78
SAEs	18	9.78
All AEs & SAEs	4	2.17
AEs in greater than x% in any group	7	3.8
AEs in greater than x% in treatment group	2	1.09
AEs in greater than x% in all patients	1	0.54
AEs in greater than x% in any group & all SAEs	2	1.09
AEs in greater than x% in treatment group & all SAEs	1	0.54
AEs in greater than x% in all patients & all SAEs	3	1.63
AEs in greater than x% in treatment group & all SAEs	1	0.54
AEs in greater than x% in treatment group & greater than in control group & all SAEs	1	0.54
SAEs in greater than x% in any group	1	0.54
AEs in greater than x% in any group & SAEs in greater than y% in any group	1	0.54
AEs in greater than x% in any group & AEs of special interest	2	1.09
Treatment related AEs	5	2.72
Treatment related AEs in greater than x% in any group	2	1.09
Grade 3>= events	2	1.09
Predefined AEs	8	4.35
AEs of special interest	1	0.54
AEs leading to study drug discontinuation/interruption	2	1.09
AEs leading to study drug discontinuation & SAEs	1	0.54
Grade 3>= events leading to study drug discontinuation & grade 3>= laboratory results	1	0.54
Treatment related AEs & AEs leading to study drug discontinuation	1	0.54
AEs in greater than x% in all patients leading to treatment discontinuations, SAEs in greater than x% in any group, serious predefined/special interest AEs and clinically significant laboratory results	1	0.54
AEs in greater than x% in any group, treatment related AEs in greater than x% in any group, treatment related SAEs and select AEs	1	0.54
Clinical laboratory data	1	0.54
Predefined AEs, AEs leading to hospitalisation/death/study drug discontinuation/interruption & SUSARS	3	1.63
Deaths	2	1.09
Some form of overall summary	5	2.72
Not specified how selected	2	1.09
Not summarised in the appendix	84	45.65

Table A7: Population used for AE analysis

Analysis population	n	%
Those that took at least a single dose	75	40.76
All randomised	54	29.35
Randomised and not withdrawn/ineligible	19	10.33
Not specified	17	9.24
Not applicable	3	1.63
Took a single dose and underwent AE/toxicity assessment	3	1.63
Active treatment groups	2	1.09
Completed treatment and assessed for primary outcome	2	1.09
Other	2	1.09
Patients who treatment was at least attempted on	1	0.54
Intention-to-treat population	1	0.54
Randomised and assessed for primary outcome	1	0.54
Randomised and attended at least on follow-up visit	1	0.54
Randomised and remained in follow-up	1	0.54
Randomised and underwent AE/toxicity assessment	1	0.54
Randomised, eligible and received at least a single dose	1	0.54
