# Supplementary material for: Analysis and reporting of adverse events in randomised controlled trials: a review
Source: BMJ Open. 2019 Mar 1;9(2):e024537. doi: 10.1136/bmjopen-2018-024537 (PMC6398660; doi:10.1136/bmjopen-2018-024537)
Supplement: Author's manuscript [file bmjopen-2018-024537.draft_revisions.pdf]

BMJ Open is committed to open peer review. As part of this commitment we make the peer review history of every article we publish publicly available.

When an article is published we post the peer reviewers' comments and the authors' responses online. We also post the versions of the paper that were used during peer review. These are the versions that the peer review comments apply to.

The versions of the paper that follow are the versions that were submitted during the peer review process. They are not the versions of record or the final published versions. They should not be cited or distributed as the published version of this manuscript.

BMJ Open is an open access journal and the full, final, typeset and author-corrected version of record of the manuscript is available on our site with no access controls, subscription charges or pay-per-view fees (http://bmjopen.bmj.com).

If you have any questions on BMJ Open's open peer review process please email info.bmjopen@bmj.com

BMJ Open

Lessons to learn from the reporting of adverse events in randomised controlled trials: a systematic review

Journal:	*BMJ Open*
Manuscript ID	bmjopen-2018-024537
Article Type:	Research
Date Submitted by the Author:	01-Jun-2018
Complete List of Authors:	Phillips, Rachel; Imperial College London, Faculty of Medicine, School of Public Health<br>Hazell, Lorna; Drug Safety Research Unit, Clinical Research; University of Portsmouth, Associate Dept. of Pharmacy and Biomedical Sciences<br>Sauzet, Odile; Universität Bielefeld,<br>Cornelius, Victoria; Imperial College London Faculty of Medicine, School of Public Health
Keywords:	Randomised controlled trials, Harm data, Adverse drug reactions, Systematic review, Investigational drug, Adverse events < THERAPEUTICS

SCHOLARONE™
Manuscripts

Lessons to learn from the reporting of adverse events in randomised controlled trials: a systematic review

Authors:

Rachel Phillips* - Imperial College London, London, United Kingdom

Lorna Hazell – Drug Safety Research Unit, Southampton, United Kingdom

Odile Sauzet – Bielefeld University, Bielefeld, Germany

Victoria Cornelius - Imperial College London, London, United Kingdom

* Corresponding author

Address: Imperial Clinical Trials Unit, Imperial College London, 1st Floor Stadium House, 68 Wood Lane, London, W12 7RH

Email: r.phillips@imperial.ac.uk

Telephone: 020 759 49356

Word Count: 3788

For peer review only

For peer review only - http://bmjopen.bmj.com/site/about/guidelines.xhtml

Abstract

Objective

To ascertain current approaches to the collection, reporting and analysis of adverse events (AEs) in randomised controlled trials (RCTs).

Design

Systematic review of clinical trials of drug interventions from four high impact medical journals.

Data sources

Electronic contents table of the Lancet, the BMJ, the New England Journal of Medicine (NEJM) and the Journal of the American Medical Association (JAMA) were searched for reports of original RCTs published between September 2015 and September 2016.

Methods

A pre-piloted checklist was used and single data extraction was performed by three reviewers with independent check of a randomly sampled subset to verify quality. We extracted data on trial characteristics, collection methods, assessment of severity and causality, reporting criteria, analysis methods and presentation of AE data.

Results

We identified 184 eligible reports (BMJ n=3; JAMA n=38, Lancet n=62; and NEJM n=81). Sixty-two percent reported some form of spontaneous AE collection but only 29% included details of specific prompts used to ascertain AE data. Numbers that withdrew from the trial were well reported (80%),

For peer review only

however only 35% of these reported whether the withdrawals were due to AEs. Results presented and analysis performed was predominantly on 'patients with at least 1 event' with 84% of studies ignoring repeated events. Despite a lack of power to undertake formal hypothesis testing, 47% performed such tests for binary outcomes.

Conclusions

This review highlighted that the collection, reporting and analysis of AE data in clinical trials is inconsistent and RCTs as a source of safety data are underutilised. Areas to improve include reducing information loss when analysing at patient level and inappropriate practice of underpowered multiple hypothesis testing. Implementation of standard reporting practices could enable a more accurate synthesis of safety data and development of guidance for statistical methodology to assess causality of AEs could facilitate better statistical practice.

Keywords

Randomised controlled trials; adverse events; harm data; adverse drug reactions; systematic review; investigational drug.

For peer review only

For peer review only - http://bmjopen.bmj.com/site/about/guidelines.xhtml

Article Summary

Strengths and Limitations of this study

1. This is the first systematic review to examine and quantify analysis practices for AEs in RCTs.
2. This review characterises what those leading the field in clinical trials are doing and provides some examples of good practice that could be adopted.
3. Articles included in this review were published in four of the top ranked medical journals therefore results are likely to be biased towards better findings than we would expect if we included all RCTs.
4. At present there is no guidance as to the best statistical methodology to assess causality of AEs in RCTs.

For peer review only - http://bmjopen.bmj.com/site/about/guidelines.xhtml

INTRODUCTION

The methods to analyse and report beneficial effects from randomised controlled trials (RCTs) are well developed but this progress has not been matched for adverse event (AE) outcomes. An adverse event is defined as 'any untoward medical occurrence that may present during treatment with a pharmaceutical product but which does not necessarily have a causal relationship with this treatment'.[1] An adverse drug reaction (ADR) is defined as 'a response to a drug which is noxious and unintended …' where a causal relationship is 'at least a reasonable possibility'.[1, 2] RCTs provide an opportunity to compare rates of AEs between arms allowing causality to be evaluated. However current analysis and reporting practices are inadequate.

Previous studies have examined the methods for AE collection and presentation, and highlighted the inadequacies in AE reporting in journal articles.[3-12] In 2004 the Consolidated Standards of Reporting Trials (CONSORT) Group produced an extension to their guidelines for reporting trial results to cover the reporting of harms, however implementation of these guidelines has been shown to be poor.[6, 10-13] Recently a joint pharmaceutical/journal collaboration published practical guidance and examples on what should be reported in journal articles and how it should be displayed to ensure transparency and aid clinical interpretation. They promote the use of clinical judgement in reporting rather than mandatory guidance.[14] However there remains uncertainty about best practice for reporting, analysing and presenting AE data.

There are many challenges associated with analysing and reporting AEs in clinical trials. RCTs are typically designed to determine the efficacy of an intervention but are often underpowered to detect important differences in AEs between arms which may suggest an ADR. Often large numbers of AEs are reported during a study, sometimes exceeding the number of patients in the clinical trial.

For peer review only - http://bmjopen.bmj.com/site/about/guidelines.xhtml

Performing hypothesis tests on these AEs would lead to issues of multiplicity, however any adjustment for multiplicity would make a 'finding untenable'.[15, 16] The use of hypothesis testing may result in the medicinal product being deemed unsafe and a trial being halted too early due to a chance imbalance, or conversely deemed safe and not stopped early enough resulting in more patients than necessary suffering an ADR.[15, 17, 18] Unlike efficacy outcomes which are well defined and restricted in number at the planning stage of a RCT, we collect numerous, undefined AEs in RCTs. Furthermore, AE collection requires additional information to be obtained on factors such as severity, timing and duration, number of occurrences and outcome, which for our efficacy outcomes would have all been predefined.

The aim of this review was to evaluate current best practice for collection, reporting and analysis of AEs in RCTs. The aim being to identify and promote any areas of good practice, whilst highlighting any areas for improvement.

METHODS

Search strategy

Four high impact medical journals that publish clinical trials of drug interventions were selected: The Lancet (Impact Factor (IF) 44.00), the BMJ (IF 20.79), the New England Journal of Medicine (NEJM, IF 72.41) and the Journal of the American Medical Association (JAMA, IF 44.41). We manually searched the electronic contents table of the journals for reports of original RCTs published between September 2015 and September 2016, inclusive.

For peer review only - http://bmjopen.bmj.com/site/about/guidelines.xhtml

Selection criteria

The inclusion criteria were phase II-IV RCTs of drug interventions where the primary outcome was efficacy of the intervention. We did not restrict according to number of treatment arms and included both parallel and cluster RCTs. We excluded cross-over RCTs, RCTs with adaptive randomisation, observational studies, case reports, editorials and letters. We also excluded RCTs where the intervention was not a drug product (i.e. not classified as a clinical trial of an investigational medicinal product (CTIMP)). As the study aimed to assess how authors report and analyse AEs in efficacy trials, trials that were specifically designed to investigate safety as a primary outcome were not included.

Data extraction

Potentially eligible articles were identified based on titles and abstracts and the full text of these studies were retrieved. Supplementary material was also reviewed if readers were referred here from the main article for further results. Supplementary Table A1 lists all data items captured with guidance given to the reviewers for extraction. We focused on the following areas: how AE data was collected (mode of collection, timing) and defined (coding, attribution); how AEs were assessed in terms of severity of the event or relatedness to the medical intervention; if there was any planned AE analysis (final and interim monitoring plans and analysis populations); how events were selected for inclusion in the journal article; how summary event information was presented in the journal article and how AEs were analysed.[7]

For peer review only - http://bmjopen.bmj.com/site/about/guidelines.xhtml

The data extraction sheet was piloted and then single data extraction was performed by three reviewers (RP, VC and LH) with 10% independent check of a randomly sampled subset to verify quality. Where specific items were flagged for poor agreement these were re-extracted. Agreement between authors was over 80%. Any queries during data extraction were shared and disagreements between reviewers were resolved through discussion.

Data analysis

The proportion of trials reporting each item, 3-4 and 8-34 in supplementary Table A1 were calculated and summary statistics (median and ranges) were calculated for items 5-7. All analyses were performed in Stata version 15.[19]

RESULTS

Study characteristics

The search identified 184 eligible trial reports (BMJ n=3; JAMA n=38, Lancet n=62; and NEJM n=81) in which a total of 496911 participants were randomised with a median of 556 participants per trial (range 30, 205513; interquartile range (IQR) 281, 1704). The median trial follow-up was 52 weeks (range 48 hours to 10 years; IQR 24, 104 weeks) and 93% were multi-centre trials. Fifty-percent of studies had an active comparator and over 50% of trials received some element of industry funding (Table 1).

For peer review only - http://bmjopen.bmj.com/site/about/guidelines.xhtml

For peer review only

Table 1: Characteristics of included studies

Characteristic	N=184		
	**Median**	**(IQR)**	**min, max**
**Sample size**	556	(281, 1704)	30, 205513
**Centres**[a]	35	(12, 100)	1, 1368
**Trial duration (weeks)**[b]	52	(24, 104)	0.3, 521
	**n**	**%**	
**Journal**			
**BMJ**	3	1.6	
**JAMA**	81	44.0	
**Lancet**	38	20.7	
**NEJM**	62	33.7	
**Funded by**			
**Public**	70	38.3	
**Industry**	80	43.7	
**Both**	33	18.0	
**Centre**			
**Single-centre**	12	7.0	
**Multi-centre**	161	93.0	
**Control**			
**Placebo**	95	51.6	
**Active**	80	43.5	
**Both**	8	4.4	
**Neither**[c]	1	0.5	

Abbreviations: *IQR = Inter-quartile range; min = minimum; and max = maximum*

[a]11 reports did not specify the number of centres

[b]2 reports did not specify trial duration

[c]One trial compared interventional drug to behavioural change intervention

For peer review only

Collection and assessment methods

Sixty-two percent of reports made reference to some form of passive (e.g. spontaneously reported by patients) AE monitoring or collection methods but only 29% of reports included specific details (prompts e.g. questions about specific events or AEs in general, questionnaires, or diaries) regarding these collection methods (Table 2, examples 1-2).[20, 21] The timing of collection was well documented

(91%, 48 out of 53 reports) in the reports that included specific details about the prompts used to collect AEs. Although specific details on clinical examinations (e.g. vital signs and blood pressure) and laboratory tests were not widely reported (only 57% of reports (95 out of 166 reports with clinical examinations and/or laboratory results presented) included details on the timing of such assessments) it was often clear from the results presented that participants had undergone these assessments (83% and 79% of studies reported clinical and laboratory results respectively) (Table 3).

Table 2: Examples of reporting practice in reviewed articles

Example no.	Study	Example practice	Example
1	Litonjua et al.[20]	Description of AE collection method	*"Study staff met with pregnant women monthly to administer a brief health questionnaire, assess medication use, and monitor for complications (via the questionnaire and medical record review)… After delivery, children were monitored by telephone every 3 months and in-person annually for 3 years, during which time infants' health, respiratory symptoms, and medications were assessed"*
2	Miller et al.[21]	Description of AE collection method	*"Safety evaluations included physical examinations, assessment of vital signs, clinical laboratory tests, and reporting of adverse events at each study visit"*
3	Libman et al.[22]	Description of planned AE analysis	*"The proportions of participants experiencing any adverse event, any related adverse event, any gastrointestinal event, any event other than a gastrointestinal event, at least 1 severe hypoglycaemic event, and at least 1 diabetic ketoacidosis event in each treatment group were compared using the Fisher exact test. The number of adverse events, new adverse events, serious adverse events, and non-serious adverse events were compared between groups using a Wilcoxon rank sumtest."*
4	Gross et al.[23]	Description of planned AE analysis	*"Safety analyses and secondary efficacy analyses used binomial regression, analysis of covariance, or the marginal Cox proportional hazards model as appropriate"*

For peer review only - http://bmjopen.bmj.com/site/about/guidelines.xhtml

Table 3: Collection, assessment and analysis methods reported by studies

Section	Component	Data item	N=184	
**Collection**			n	%
	**How was AE/harm information collected?**			
		Passive collection	114	62.0
		Prompted collection	53	28.8
		Active screening		
		Clinical examinations	153	83.2
		Laboratory tests	146	79.4
	**Timing of prompted collection specified** (n=53)		48	90.6
	**Timing of active collection specified** (n=166)		95	57.2
	**Which, if any, dictionary was used to code AE data?**			
		CTCAE	18	9.8
		MedDRA	43	23.4
		CTCAE and MedDRA	1	0.5
		DAIDS	2	1.1
		ICD-10	1	0.5
		Researcher defined	2	1.1
		Other	3	1.6
		No dictionary reported	114	62.0
**Assessment**				
	**Who assigned attribution to study drug?**			
		Blinded assessor	9	4.9
		Unblinded assessor	7	3.8
		Both	1	0.5
		Not specified	164	89.1
		Not applicable[a]	3	1.6
**Analysis**				
	**Was any analysis for AEs specified in the methods section?**			
		Yes	57	31.0
	**Was a population for AE analysis specified?**			
		Yes	82	44.6
	**Was there a planned interim analysis with stopping criteria?**			
		No	138	75.0
		Yes for efficacy	24	13.0
		Yes for efficacy & futility	11	6.0
		Yes for efficacy & safety	3	1.6
		Yes for efficacy, futility & safety	2	1.1
		Yes but no other details given	6	3.3

Abbreviations: *AE = Adverse event; CTCAE = Common Terminology Criteria for Adverse Events; MedDRA = Medical Dictionary for Regulatory Activities; DAIDS = The Division of AIDS; and ICD-10 = International Classification of Diseases 10th revision.*

NOTE: Denominator specified in item column if it differs from total sample

[a]3 reports included no AE data

For peer review only - http://bmjopen.bmj.com/site/about/guidelines.xhtml

Prespecified analysis

Thirty-one percent of reports provided information on the planned analysis for AEs in the statistical analysis section of the paper and 45% pre-specified a safety population (Table 2, examples 3-4 and Table 3).[22, 23] A quarter of trials reported planned interim analysis with stopping criteria (Table 3), five (2.7%) of which included specific criteria on stopping for a harmful event (Supplement Table A2).

Selection of AEs and reporting practices

Five trials did not report any information on AEs. Two of these reports made the following statements "*there were no significant adverse events related to the procedure*" and "*no excess in mortality or major adverse events were found…*" and three made no mention of AEs.[24-28]

Twenty-four (13%) trials only provided a summary of the number of AEs or serious AEs rather than listing the actual AEs that occurred. For example "*Six serious adverse events occurred in the acetaminophen group and 12 in the ibuprofen group.*"[29] Of these 24 trials, 10 did provide specific details of the types of events in an appendix. This means 8% of trials either did not report AEs or only included a summary (Table 4).

For peer review only - http://bmjopen.bmj.com/site/about/guidelines.xhtml

Table 4: Summaries of results presented by studies

Component	Data item	N=184	
		n	%
**What was reported in the manuscript?**			
	Actual AE terms	73	39.7
	Summaries of AE type (e.g. AE, SAE)	24	13.0
	Both	80	43.5
	Neither	7	3.8
**What was reported in the appendix?**			
	Actual AE terms	76	41.3
	Summaries of AE type (e.g. AE, SAE)	7	3.8
	Both	22	12.0
	Neither	3	1.6
	Not applicable[a]	76	41.3
**Which population was the AE analysis performed on?**			
	All randomised	54	29.4
	Those that took at least a single dose	75	40.8
	Other	35	19.0
	Not specified	17	9.2
	Not applicable[b]	3	1.6
**Were drop-outs/withdrawals reported?**			
	No	33	17.9
	Yes by treatment arm	144	78.3
	Yes overall	2	1.1
	Not applicable[c]	5	2.7
**Were withdrawals due to AEs reported?** (n=146)			
	No	89	61.0
	Yes	51	34.9
	Not applicable[d]	6	4.1
**Were specific AEs causing withdrawals reported?** (n=51)			
	No	39	76.5
	Yes	12	23.5
**How were binary AE outcomes summarised by arm?**			
	Not summarised[e]	6	3.3
	Number of people with an event	154	83.7
	Number of events	11	6.0
	Both	12	6.5
	Unclear	1	0.5
**Were frequencies of AEs reported by arm?**			
	No	5	2.7
	Yes for some	13	7.1
	Yes for all	160	87.0
	Not applicable[e]	6	3.3
**Were percentages of AEs reported by arm?**			
	No	18	9.8
	Yes for some	25	13.6

For peer review only - http://bmjopen.bmj.com/site/about/guidelines.xhtml

For peer review only

	Yes for all	135	73.4
	Not applicable[e]	6	3.3
**Were between arm differences and 95% CI of AEs reported?**			
	No	141	76.6
	Yes for some	18	9.8
	Yes for all	19	10.3
	Not applicable[e]	6	3.3
**Were statistical significance tests between arms on AEs reported?**			
	No	92	50.0
	Yes for some	31	16.9
	Yes for all	55	29.9
	Not applicable[e]	6	3.3
**Were continuous AEs outcomes dichotomised for summaries?**			
	No	10	5.4
	Yes for some	28	15.2
	Yes for all	108	58.7
	Not applicable	38	20.7
**If continuous outcomes were left as continuous what between arm analyses was performed?** (n=38)			
**Differences in measures of central tendency estimated with 95% CI**			
	No	23	60.5
	Yes for some	1	2.6
	Yes for all	14	36.8
**Between arm hypothesis tests performed**			
	No	12	31.6
	Yes for some	2	5.3
	Yes for all	24	63.2
**Were any 'signal detection' approaches used?**			
	No	184	100.0
	Yes	0	0.0
**Were there any graphical presentations of AE outcomes?**			
	No	162	88.0
	Yes	22	12.0
**Were summaries of severity rating of AEs reported?**			
	No	103	56.0
	Yes for some	41	22.3
	Yes for all	35	19.0
	Not applicable[f]	5	2.7
**Were number of serious AEs reported?**			
	No	44	23.9
	Yes overall	2	1.1
	Yes by treatment arm	132	71.7
	Not applicable[g]	6	3.3
**For serious AEs was relatedness given?** (n=134)			
	No	77	57.5
	Yes for some	18	13.4
	Yes for all	38	28.4

For peer review only

For peer review only - http://bmjopen.bmj.com/site/about/guidelines.xhtml

	Yes overall	1	0.8
**Were there any AEs where information on duration of events was reported?**			
	No	175	95.1
	Yes	9	4.9
**Were there any AEs where information on the time of occurrence of events was reported?**			
	No	132	71.7
	Yes	52	28.3
**If any significance tests were performed on AEs was multiplicity of events accounted for?**			
	No	81	44.0
	Yes	3	1.6
	Not applicable	100	54.4
**Did the report reference the CONSORT extension to harms**			
	No	184	100.0
	Yes	0	0.0

Abbreviations: *AE = Adverse Event; SAE = Serious Adverse Event; CI = Confidence Interval; and CONSORT = Consolidated Standards for Reporting Trials.*

[a] Make no reference to the appendix

[b] 3 reports included no AE data

[c] 5 reports indicate no withdrawals

[d] 6 reports specify the number of withdrawals and reasons but none of the reasons are related to AEs

[e] This includes 3 reports with no AE data (as per footnote [b]), 2 reports that provide generic statements regarding AE data and 1 report that only reported continuous outcomes

[f] This includes 3 reports with no AE data and 2 reports that provide generic statements regarding AE data (as per footnote [e])

[g] 6 papers specifically state that no serious adverse events occurred

Eighty-nine percent of trials reported a subset of all the AEs they collected. How AEs are 'selected' for inclusion in the article was not consistent or clear, and in 3% of studies it was impossible to discern how the authors had selected the AEs they presented for inclusion. Twenty-six percent of reports selected events based on a frequency threshold e.g. events experienced by greater than x% in any group; 9% of reports used a measure of severity to select events e.g. AEs of grade 3 or higher; 23% of reports included events based on seriousness; and 8% included AEs based on relatedness to treatment (percentages are not independent as the majority of reports used several different criteria for selection). Supplementary Table A3 provides full details of selection criteria used.

For peer review only

For peer review only - http://bmjopen.bmj.com/site/about/guidelines.xhtml

We found that 41% of trials analysed AEs in participants that received at least one dose, 29% of trials used all randomised participants and 9% did not specify the analysis population (Table 4). Further details on analysis populations used are given in supplementary Table A5.

Nearly 80% of trials reported the number of participants who withdrew from the trial; of these 35% (51 of 146 reports) reported whether the withdrawals were due to AEs and of these 24% (12 of 51 reports) reported the actual events that caused withdrawals. Results presented and analysis performed was predominantly on 'patients with at least 1 event' with 84% of reports providing no information on the number of events occurring. An example of how to incorporate information on number of events is presented in [30]. Forty-one percent of trials reported information on the severity of AEs. Five percent of trials include a report of at least one event with duration, but presenting such data is limited in the main report. The trials that did present this information did so in a variety of ways. For example incorporating the information into the AE table with summary statistics such as the mean duration of certain events or presenting it for a subgroup of events in the footnotes of AE tables e.g. "*One event of non-serious squamous cell carcinoma (day 210, resolved on day 215; adalimumab treatment was not interrupted).*"[31-33] Twenty-eight percent of reports included information on the timing of AEs (Table 4).

Serious adverse events were typically well documented (73%) and six reports (3%) explicitly stated that no serious events had occurred. However for forty-four reports (24%) it was not possible to discern if no serious events had occurred or whether they were simply omitted from the report. Forty-two percent (57 of 134 reports) of reports included details on whether the events had been classified as related to the intervention (Table 4).

For peer review only - http://bmjopen.bmj.com/site/about/guidelines.xhtml

Analysis of AE outcomes

Binary

The majority of trials summarised binary outcomes using frequencies (94%) and percentages (87%). Despite a lack of power to undertake formal hypothesis testing, 47% reported p-values for binary outcomes. For example "*There were no between-group differences in the rate of patients with at least 1 adverse event (16.7% [14 patients] in the clopidogrel group vs 21.8% [19 patients] in the placebo group; difference, −5.2% [95% CI, −17% to 6.6%]; P = .44).*" However with a total safety population of 171 such a test would have only had 13% power to detect such a difference and was therefore substantially underpowered. The conclusion that "*No significant increase in adverse events was observed*" makes no reference to the 95% confidence interval presented which indicates that the findings were in fact compatible with a 17% decrease in experiencing at least on AE as well as a near 7% increase.[34]

Continuous

There was a pervasive practise (59%) of categorising continuous clinical and laboratory outcomes. Of the trials that did not dichotomise continuous AE data nearly 70% performed some form of statistical significance testing (Table 4). Whilst continuous outcomes do not suffer to the same degree regarding lack of power, multiple testing is still a problem, however no multiplicity corrections for continuous outcomes were performed.

Of the trials that performed statistical significance testing on AE data, only three made an adjustment for multiplicity of tests (all three on dichotomised outcomes).[31, 35, 36] Two of which used a Bonferroni correction and adjusted for the number of pairwise comparisons between each of the

For peer review only - http://bmjopen.bmj.com/site/about/guidelines.xhtml

treatment groups for each individual event rather than the total number of significance tests performed. As such both analyses would have still been effected by issues of multiple testing.

Twelve percent of reports used graphs to illustrate AE data (Table 4). The CONSORT extension highlighted the value of graphs for summarising such data, especially for conveying information on time-to-event outcomes.[37] An example of such a plot is included in the supplement of [38] (eFigure 2).

DISCUSSION

The safety profile of a medicinal product is established through evidence collected from several sources including clinical trials, observational studies and spontaneous reports.[39] The advantage of clinical trial data is that these provide a controlled comparison of the rate of AEs allowing causality to be evaluated but have the disadvantage that the sample size is often not large enough to detect rare ADRs.

To ensure that a useful and comprehensive picture of the safety profile is provided to all relevant parties clear reporting of AEs from clinical trials is required. Current research has shown the quality of reporting is substandard.[3-12] The aim of this study was to review best practice across four leading medical journals for AE collection, analysis and reporting practices, highlighting any areas for improvement and examples of good practice.

Principal Findings

Collection and assessment methods

For peer review only - http://bmjopen.bmj.com/site/about/guidelines.xhtml
For peer review only

The CONSORT extension to harm was developed with the aim to improve reporting of safety data in RCTs.[37] Of the ten recommendations many were not well reported.[13] This suggests that the CONSORT extension is not being routinely adopted by authors to aid their reporting. Most journals now request that authors include a completed CONSORT checklist when they submit their article but we are not aware of any journal that request the CONSORT harm extension to also be submitted. Of the four journals in this review the Lancet is the only journal that makes specific reference to the harm extension in their guidelines to authors. The CONSORT statement contains a single item related to safety, item 19: 'all important harms or unintended effects in each group' should be reported.[37] This may explain why some items listed on the CONSORT extension for harm were reported by so few trials. The adoption of CONSORT harms by journals may support better reporting.

We found that the method of AE collection was poorly reported. This has important implications for the type and frequency of AEs reported with "passive collection resulting in fewer recorded AEs".[40, 41] Where the method was given the timing of collection was typically also reported and we would recommend continuation of this practice. The frequency of AE collection has further important implications on the number of events reported. More frequent assessment and longer follow-up will result in more AEs reported.[13] It is important to consider these factors when making conclusions about the safety profile.

The method of attribution between drug and AE was another area where reporting practice was inadequate. However the joint pharmaceutical/journal collaboration indicate that such attribution has 'limited value' given the 'inherent subjectivity in such attribution'.[14]

For peer review only - http://bmjopen.bmj.com/site/about/guidelines.xhtml

Prespecified analysis

We found that formal assessments of AEs regarding stopping for emerging ADRs utilising statistical rules was rare. Subjective assessments of overwhelming amounts of data could easily lead to potential signals of harm being missed. There could be benefits to incorporating more objective methods alongside clinical review to monitor AE information, both for interim analysis by data monitoring committees (DMCs) and final trial analysis to help better identify drug harm relationships. Graphical displays have gone some way towards aiding interpretation.[42-46]

Selection of AEs and reporting practices

Due to space constraints in journal reports AE information is often included in the appendix. Whilst we encourage use of appendices and supplementary material for including additional detail on AEs, we caution authors against depositing all AE data into such documents without attempting to present a summary of the AE profile in the main article. It is important that the main report strikes a balance between efficacy and harm therefore allowing a risk-benefit assessment to be made solely from the article.

The failure to report any information on AEs restricts interpretation and prevents a risk-benefit assessment. We identified two reports that made generic summaries of the overall safety profile and it was clear in both that there had been harmful effects however the authors did not include any further information. Three reports contained no information leaving readers uninformed as to any additional information these studies may provide on the safety profile. Ambiguous reporting prevents building an accurate picture of the safety profile. As such profiles are developed on

For peer review only - http://bmjopen.bmj.com/site/about/guidelines.xhtml

accumulating evidence, it is important that each study report to the same standard and information is not wasted.

We found that the selection criteria used by authors to decide what AEs to include in the report were arbitrary and inconsistent. This will have important implications when synthesising data across studies to construct safety profiles. Authors would benefit from guidance to facilitate consistency but currently research in this area is lacking. Lineberry et al. recommended clinically relevant events that should always be reported (deaths, SAEs and events leading to discontinuation of intervention) and criteria that should be considered when deciding what other AEs to report e.g. interest based on the disease(s) under investigation, comorbidities of the study population, intervention mechanism, trial duration.[14] Standard outcomes for a drug class would be one potential solution to avoid issues of inconsistency suggested by Cornelius et al.[7]

CONSORT recommend that AE analyses should be performed on the intention-to-treat (ITT) population to maintain the random assignment.[13] However it is clear from our review that this population label is not always appropriately and consistently applied. There is a tendency for studies to make modifications to the ITT population. Using the ITT or modified-ITT population is likely to underestimate the risk by inflating the denominator with participants who may have never received the study drug.[47] Such estimates are appropriate for health economic evaluations where estimates of the cost-effectiveness will inform policy level decisions regarding how to treat the population. However a more appropriate population for AE analysis to inform prescriber and patient decisions may be those that receive at least one dose. It is important that authors are clear about what they are estimating and how this affects their conclusions.

For peer review only - http://bmjopen.bmj.com/site/about/guidelines.xhtml

Proxy outcomes can be used as a measure of the impact of AEs on patients. Examples include the number of withdrawal due to any reason, withdrawals due to AEs, the number of events an individual experiences, the severity of the AE and the duration. A high proportion of trials reported withdrawal for any reason and this is likely to be as a result of the CONSORT recommendations.[37] The other outcomes were not frequently reported and increasing this could facilitate interpretation.[13] This information would permit better evaluation of the impact of AEs and the tolerability of the intervention to inform patients' and clinicians' treatment decisions. Reporting numbers that experience at least one event only and not providing information on repeated events masks valuable information that may be important to the patient and the cost-effectiveness evaluation. For example, chronic, repeated headaches over an extended duration will have an important impact for patients compared to a single headache or headaches over a short duration but it is not possible to distinguish between these two scenarios when reported as 'at least one event'.[14] Severity of events was also an important aspect that was often not differentiated. For example there would be different impact on patients' quality-of-life with mild compared to severe nausea and which could lead to changes in dosing regimens. Displaying such information for all AEs in tables would soon become overwhelming and make interpretation difficult. Graphical approaches have been suggested as a solution to aid review. Examples of such a plots can be found in [48]. Online appendices and supplementary material provide more opportunity to include this important information.

For serious adverse events information on the time of likely onset can be useful information to inform patient monitoring plans. For example the documented risk of suicide and suicidal ideation within the first few weeks of starting anti-depressant allows patients and prescribers to remain alert and monitor closely for this period. Nearly a third of reports included such information and we would encourage authors to adopt this practice.

For peer review only - http://bmjopen.bmj.com/site/about/guidelines.xhtml

Analysis of AE outcomes

The majority of trials in this review included a balanced report of AEs alongside benefit. However many included generic statements regarding the safety profile such as 'the intervention was well tolerated' or 'the intervention exhibited a good safety profile' and these were frequently based on post-hoc statistical tests. Guidelines caution against such tests.[14] The results of which are difficult to interpret as a lack of significance does not indicate that the intervention is safe and conversely multiple testing without adjustment will increase the number of significant differences due to chance.[49, 50]

Graphs are an efficient method to convey and interpret large amounts of data and can make it easier to flag potential safety signals.[45, 46, 48] Twenty-two studies included in the review used graphs to present AE data and an example of one such report is given in the supplementary eTable of [51].

Limitations of trials

Trials are a valuable source for high quality adverse event data but compared to observational studies have smaller sample size, follow-up periods and generalisability which restrict the ability to detect rare ADRs, ADRs with long latency and drug interactions in complex populations. The typical duration of a trial means there is often insufficient follow-up to fully characterise the safety profile as it provides limited information on long-term exposure. Stringent inclusion criteria restrict the

For peer review only - http://bmjopen.bmj.com/site/about/guidelines.xhtml

population the intervention is assessed in and so limited information on drug-interactions is obtained.[17]

Limitations of this study

Articles included in this review were published in four of the top ranked medical journals therefore results are likely to be biased towards better findings than we would expect if we included all RCTs. However this review characterises what those leading the field are doing and provides some examples of good practice that could be adopted.

Conclusions and recommendations for future work

RCTs are a valuable source of information establishing the safety profile of medicinal products. Our review has demonstrated that data is not currently being fully utilised. Analysis of AE data is frequently inappropriate and reports often provide insufficient and inconsistent information to allow a comprehensive summary of the safety profile to be established.

This research has identified two areas that would benefit from future research. i) Improving the consistency of reporting important AE outcomes across trials to facilitate comparison and synthesis. This is in line with work from the COMET Initiative group (http://www.comet-initiative.org/). The development of CORE safety outcomes by drug class could be considered.[7] ii) Evaluation of methods to analyse AEs in RCTs.

For peer review only - http://bmjopen.bmj.com/site/about/guidelines.xhtml

COMPETING INTERESTS

None declared.

FUNDING

This research was supported by the NIHR grant number DRF-2017-10-131.

DATA SHARING STATEMENT

No additional data are available.

AUTHOR STATEMENT

RP conceived the idea for this review, conducted the search, carried out data extraction and analysis, and wrote the manuscript. VC conceived the idea for the review, performed data extraction and critical revision of the manuscript. LH performed data extraction and critical revision of the manuscript. OS performed critical revision of the manuscript.

For peer review only - http://bmjopen.bmj.com/site/about/guidelines.xhtml

REFERENCES

1. Edwards IR and Biriell C. Harmonisation in pharmacovigilance. *Drug safety* 1994; 10: 93-102. DOI: 10.2165/00002018-199410020-00001.

2. The International Council for Harmonisation of Technical Requirements for Pharmaceuticals for Human Use (ICH). ICH Harmonised Tripartite Guideline. E2A Clinical Safety Data Management: Definitions and Standards for Expedited Reporting. 1994.

3. Ioannidis JPA and Contopoulos-Ioannidis DG. Reporting of safety data from randomised trials. *The Lancet* 1998; 352: 1752-1753. DOI: 10.1016/S0140-6736(05)79825-1.

4. Edwards JE, McQuay HJ, Moore RA, et al. Reporting of Adverse Effects in Clinical Trials Should Be Improved. *Journal of Pain and Symptom Management* 1999; 18: 427-437. DOI: 10.1016/S0885-3924(99)00093-7.

5. Ioannidis JA and Lau J. Completeness of safety reporting in randomized trials: An evaluation of 7 medical areas. *JAMA* 2001; 285: 437-443. DOI: 10.1001/jama.285.4.437.

6. Pitrou I, Boutron I, Ahmad N, et al. Reporting of safety results in published reports of randomized controlled trials. *Archives of Internal Medicine* 2009; 169: 1756-1761. DOI: 10.1001/archinternmed.2009.306.

7. Cornelius VR, Sauzet O, Williams JE, et al. Adverse event reporting in randomised controlled trials of neuropathic pain: Considerations for future practice. *PAIN* 2013; 154: 213-220. DOI: 10.1016/j.pain.2012.08.012.

8. Maggi CB, Griebeler IH and Dal Pizzol Tda S. Information on adverse events in randomised clinical trials assessing drug interventions published in four medical journals with high impact factors. *Int J Risk Saf Med* 2014; 26: 9-22. 2014/05/07. DOI: 10.3233/JRS-140609.

9. Smith SM, Wang AT, Katz NP, et al. Adverse event assessment, analysis, and reporting in recent published analgesic clinical trials: ACTTION systematic review and recommendations. *PAIN* 2013; 154: 997-1008. DOI: 10.1016/j.pain.2013.03.003.

For peer review only

10. Peron J, Maillet D, Gan HK, et al. Adherence to CONSORT adverse event reporting guidelines in randomized clinical trials evaluating systemic cancer therapy: a systematic review. *J Clin Oncol* 2013; 31: 3957-3963. 2013/09/26. DOI: 10.1200/JCO.2013.49.3981.

11. Favier R and Crépin S. The reporting of harms in publications on randomized controlled trials funded by the "Programme Hospitalier de Recherche Clinique," a French academic funding scheme. *Clinical Trials* 2018; 0: 1740774518760565. DOI: 10.1177/1740774518760565.

12. Hum SW, Golder S and Shaikh N. Inadequate harms reporting in randomized control trials of antibiotics for pediatric acute otitis media: a systematic review. *Drug Safety* 2018 May 08. DOI: 10.1007/s40264-018-0680-0.

13. Ioannidis JA, Evans SW, Gøtzsche PC, et al. Better reporting of harms in randomized trials: An extension of the consort statement. *Annals of Internal Medicine* 2004; 141: 781-788. DOI: 10.7326/0003-4819-141-10-200411160-00009.

14. Lineberry N, Berlin JA, Mansi B, et al. Recommendations to improve adverse event reporting in clinical trial publications: A joint pharmaceutical industry/journal editor perspective. *BMJ (Online)* 2016; 355: i5078.

15. Ma H, Ke C, Jiang Q, et al. Statistical Considerations on the Evaluation of Imbalances of Adverse Events in Randomized Clinical Trials. *Therapeutic Innovation and Regulatory Science* 2015; 49: 957-965. DOI: http://dx.doi.org/10.1177/2168479015587363.

16. Food and Drug Administration. *Attachment B: Clinical Safety Review of an NDA or BLA of the Good Review Practice. Clinical Reveiw Template (MAPP 6010.3 Rev. 1)*. 2010.

17. Siddiqui O. Statistical methods to analyze adverse events data of randomized clinical trials. *Journal of Biopharmaceutical Statistics* 2009; 19: 889-899. DOI: http://dx.doi.org/10.1080/10543400903105463.

18. Tsang R, Colley L and Lynd LD. Inadequate statistical power to detect clinically significant differences in adverse event rates in randomized controlled trials. *Journal of Clinical Epidemiology* 2009; 62: 609-616.

For peer review only

19. StataCorp. Stata Statistical Software: Release 15. College Station, TX: StataCorp LLC, 2017.

20. Litonjua AA, Carey VJ, Laranjo N, et al. Effect of Prenatal Supplementation With Vitamin D on Asthma or Recurrent Wheezing in Offspring by Age 3 Years: The VDAART Randomized Clinical Trial. *JAMA* 2016; 315: 362-370. 2016/01/28. DOI: 10.1001/jama.2015.18589.

21. Miller PD, Hattersley G, Riis BJ, et al. Effect of Abaloparatide vs Placebo on New Vertebral Fractures in Postmenopausal Women With Osteoporosis: A Randomized Clinical Trial. *JAMA* 2016; 316: 722-733. 2016/08/18. DOI: 10.1001/jama.2016.11136.

22. Libman IM, Miller KM, DiMeglio LA, et al. Effect of Metformin Added to Insulin on Glycemic Control Among Overweight/Obese Adolescents With Type 1 Diabetes: A Randomized Clinical Trial. *JAMA* 2015; 314: 2241-2250. 2015/12/02. DOI: 10.1001/jama.2015.16174.

23. Writing Committee for the Diabetic Retinopathy Clinical Research Network, Gross JG, Glassman AR, et al. Panretinal Photocoagulation vs Intravitreous Ranibizumab for Proliferative Diabetic Retinopathy: A Randomized Clinical Trial. *JAMA* 2015; 314: 2137-2146. 2015/11/14. DOI: 10.1001/jama.2015.15217.

24. Aitken E, Jackson A, Kearns R, et al. Effect of regional versus local anaesthesia on outcome after arteriovenous fistula creation: a randomised controlled trial. *The Lancet* 2016; 388: 1067-1074. DOI: 10.1016/s0140-6736(16)30948-5.

25. Kaul U, Bangalore S, Seth A, et al. Paclitaxel-Eluting versus Everolimus-Eluting Coronary Stents in Diabetes. *N Engl J Med* 2015; 373: 1709-1719. 2015/10/16. DOI: 10.1056/NEJMoa1510188.

26. Cohen MS, Chen YQ, McCauley M, et al. Antiretroviral Therapy for the Prevention of HIV-1 Transmission. *N Engl J Med* 2016; 375: 830-839. 2016/07/19. DOI: 10.1056/NEJMoa1600693.

27. Isanaka S, Langendorf C, Berthe F, et al. Routine Amoxicillin for Uncomplicated Severe Acute Malnutrition in Children. *N Engl J Med* 2016; 374: 444-453. 2016/02/04. DOI: 10.1056/NEJMoa1507024.

For peer review only

28. Natalucci G, Latal B, Koller B, et al. Effect of Early Prophylactic High-Dose Recombinant Human Erythropoietin in Very Preterm Infants on Neurodevelopmental Outcome at 2 Years: A Randomized Clinical Trial. *JAMA* 2016; 315: 2079-2085. 2016/05/18. DOI: 10.1001/jama.2016.5504.

29. Sheehan WJ, Mauger DT, Paul IM, et al. Acetaminophen versus Ibuprofen in Young Children with Mild Persistent Asthma. *N Engl J Med* 2016; 375: 619-630. 2016/08/18. DOI: 10.1056/NEJMoa1515990.

30. Lind M, Hirsch IB, Tuomilehto J, et al. Liraglutide in people treated for type 2 diabetes with multiple daily insulin injections: randomised clinical trial (MDI Liraglutide trial). *BMJ* 2015; 351: h5364. 2015/10/30. DOI: 10.1136/bmj.h5364.

31. Lincoff AM, Mehran R, Povsic TJ, et al. Effect of the REG1 anticoagulation system versus bivalirudin on outcomes after percutaneous coronary intervention (REGULATE-PCI): a randomised clinical trial. *The Lancet* 2016; 387: 349-356. DOI: 10.1016/s0140-6736(15)00515-2.

32. Ribrag V, Koscielny S, Bosq J, et al. Rituximab and dose-dense chemotherapy for adults with Burkitt's lymphoma: a randomised, controlled, open-label, phase 3 trial. *The Lancet* 2016; 387: 2402-2411. DOI: 10.1016/s0140-6736(15)01317-3.

33. Nguyen QD, Merrill PT, Jaffe GJ, et al. Adalimumab for prevention of uveitic flare in patients with inactive non-infectious uveitis controlled by corticosteroids (VISUAL II): a multicentre, double-masked, randomised, placebo-controlled phase 3 trial. *The Lancet* 2016; 388: 1183-1192. DOI: 10.1016/s0140-6736(16)31339-3.

34. Rodes-Cabau J, Horlick E, Ibrahim R, et al. Effect of Clopidogrel and Aspirin vs Aspirin Alone on Migraine Headaches After Transcatheter Atrial Septal Defect Closure: The CANOA Randomized Clinical Trial. *JAMA* 2015; 314: 2147-2154. 2015/11/10. DOI: 10.1001/jama.2015.13919.

35. Ruperto N, Pistorio A, Oliveira S, et al. Prednisone versus prednisone plus ciclosporin versus prednisone plus methotrexate in new-onset juvenile dermatomyositis: a randomised trial. *The Lancet* 2016; 387: 671-678. DOI: 10.1016/s0140-6736(15)01021-1.

For peer review only

36. Whitehead KJ, Sautter NB, McWilliams JP, et al. Effect of Topical Intranasal Therapy on Epistaxis Frequency in Patients With Hereditary Hemorrhagic Telangiectasia: A Randomized Clinical Trial. *JAMA* 2016; 316: 943-951. 2016/09/07. DOI: 10.1001/jama.2016.11724.

37. Schulz KF, Altman DG, Moher D, et al. Consort 2010 statement: Updated guidelines for reporting parallel group randomized trials. *Annals of Internal Medicine* 2010; 152: 726-732. DOI: 10.7326/0003-4819-152-11-201006010-00232.

38. Lingvay I, Perez Manghi F, Garcia-Hernandez P, et al. Effect of Insulin Glargine Up-titration vs Insulin Degludec/Liraglutide on Glycated Hemoglobin Levels in Patients With Uncontrolled Type 2 Diabetes: The DUAL V Randomized Clinical Trial. *JAMA* 2016; 315: 898-907. 2016/03/05. DOI: 10.1001/jama.2016.1252.

39. Cornelius VR, Liu K, Peacock J, et al. Variation in adverse drug reactions listed in product information for antidepressants and anticonvulsants, between the USA and Europe: a comparison review of paired regulatory documents. *BMJ Open* 2016; 6. DOI: 10.1136/bmjopen-2015-010599.

40. Stephens MD, Talbot JC and Routledge PA. *The Detection of New Adverse Reactions*. 4 ed. London: Macmillan Reference 1998.

41. Bent S, Padula A and Avins AL. Brief communication: Better ways to question patients about adverse medical events: a randomized, controlled trial. *Annals of Internal Medicine* 2006; 144: 257-261. DOI: 10.7326/0003-4819-144-4-200602210-00007.

42. Davis S, Sun H and Jung K. Best practices for reporting safety data to data monitoring committees. *Trials Conference: 4th International Clinical Trials Methodology Conference , ICTMC and the 38th Annual Meeting of the Society for Clinical Trials United Kingdom* 2017; 18.

43. Furey A and Bechhofer R. Effective graphical analyses of adverse events in DMC reports. *Trials Conference: 4th International Clinical Trials Methodology Conference , ICTMC and the 38th Annual Meeting of the Society for Clinical Trials United Kingdom* 2017; 18.

For peer review only

44. Buhr KA, Downs M, Rhorer J, et al. Reports to Independent Data Monitoring Committees: An Appeal for Clarity, Completeness, and Comprehensibility. *Therapeutic Innovation & Regulatory Science* 2017: 2168479017739268. DOI: 10.1177/2168479017739268.

45. Amit O, Heiberger RM and Lane PW. Graphical approaches to the analysis of safety data from clinical trials. *Pharmaceutical Statistics* 2008; 7: 20-35.

46. Zink RC, Wolfinger RD and Mann G. Summarizing the incidence of adverse events using volcano plots and time intervals. *Clinical Trials* 2013; 10: 398-406.

47. Detry MA and Lewis RJ. The intention-to-treat principle: How to assess the true effect of choosing a medical treatment. *JAMA* 2014; 312: 85-86. DOI: 10.1001/jama.2014.7523.

48. Sample Closed Session DMC Report, https://www.biostat.wisc.edu/sites/default/files/Sample_Report_Closed_20160920.pdf (2016, accessed 26/03/2018).

49. Altman DG and Bland JM. Statistics notes: Absence of evidence is not evidence of absence. *BMJ* 1995; 311: 485. DOI: 10.1136/bmj.311.7003.485.

50. Jonville-Béra A, Giraudeau B and Autret-Leca E. Reporting of drug tolerance in randomized clinical trials: When data conflict with authors' conclusions. *Annals of Internal Medicine* 2006; 144: 306-307. DOI: 10.7326/0003-4819-144-4-200602210-00024.

51. Coovadia A, Abrams EJ, Strehlau R, et al. Efavirenz-Based Antiretroviral Therapy Among Nevirapine-Exposed HIV-Infected Children in South Africa: A Randomized Clinical Trial. *JAMA* 2015; 314: 1808-1817. 2015/11/04. DOI: 10.1001/jama.2015.13631.

For peer review only

Supplementary material

Table A1: Data items extracted from publications

			**Items collected**	**Instructions**
**Study details**		1	Study number	
		2	Journal	
		3	Funding source: public, private, both or unspecified.	Studies will be assumed to be funded by industry only if this is explicitly stated.
**Study characteristics**		4	Control: placebo, active or both	Select placebo if no active treatment is given, else active. Both should be selected for trials with multiple arms where there is at least one group receiving no active treatment and one group receiving an active treatment.
		5	Number of centres	
		6	Number randomised	
		7	Study duration (length of trial follow-up)	
**Methods**	Details of how AE outcomes were defined (coding, attribution) and were collected (mode of collection, timing)	8	Describe the collection method: passive surveillance, patient prompted, clinical examinations (e.g. vital signs or urine samples), and laboratory tests. (Select all that apply)	*Passive*: If authors state that AEs were collected throughout the study with no further information we will assume that collection was passive. *Prompted*: Prompted methods include, but are not limited to: questions about both specific events and AEs in general, questionnaires, or diaries.
		9	Stated the timing of collection.	
		10	Mention dictionary for coding of events: Researcher defined, MedDRA, CTCAE, WHO-ART, COSTART, ICD-10, other or not applicable	
		11	Describe who undertook the assessment of attribution to study drug: blinded assessor, unblinded assessor or not specified.	
**Planned analysis**	Details of any plans for analysing AE outcomes	12	Describe analysis for AE outcomes in the statistical methods.	Reference must be made to harmful events e.g. AEs or a specific harm event, this cannot be simply how binary events will be analysed.
		13	Define a 'safety' population for analysis.	
		14	Specify a planned interim analysis with stopping criteria: based on efficacy, based on safety, based on both efficacy and safety, yes but no other details given, no planned interim analysis or unclear	Criteria for stopping must be set out, it is not enough to say that the DMC reviewed the data.

For peer review only

For peer review only - http://bmjopen.bmj.com/site/about/guidelines.xhtml

**Results**	Details of what was reported and where	15	What was reported in the main paper: summaries of type of AEs (e.g. AE, SAE, AR, ADR), actual AE terms, both, neither or not applicable?	Not applicable is relevant when for example authors explicitly state that there are no events or there is only one event so summaries are inappropriate.
		16	What was reported in the appendix: summaries of type of AEs (e.g. AE, SAE, AR, ADR), actual AE terms, both, neither or not applicable?	Not applicable is relevant when for example authors explicitly state that there are no events or there is only one event so summaries are inappropriate.<br>We will only search the appendix/supplementary material for AE data if the main article makes reference to it.
		17	Who was the AE analysis performed on: all randomised, participants who took at least a single dose, other or not specified?	
		18	How were number of drop-outs/withdrawals reported: By treatment arm, overall, not reported or not applicable?	Not applicable is relevant when there are no drop-outs/withdrawals.<br>This does not include discontinuation of treatment.
		19	Were drop-outs/withdrawals due to AEs reported: Yes, no or not applicable?	Not applicable if drop-outs/withdrawals are not reported or if it is reported that there are no drop-outs/withdrawals.
		20	Were specific AEs causing withdrawals reported: Yes, no or not applicable?	Not applicable if drop-outs/withdrawals due to AEs are not reported or if it is reported that there are no drop-outs/withdrawals due to AEs.
		21	What was the selection criteria for the AEs reported?	Free text response where possibilities can include for example: most frequent, above a severity threshold, SAEs.<br>Include details of what's in the main journal article and what is in the appendix separately.
	Details of how AEs were summarised and presented - binary outcomes	22	What summary information was given: Number of people, number of events, both, unclear, not summarised or not applicable?	Only select 'number of events' if presented for each individual event not just overall number of events.<br>Not applicable is only relevant when report that there are no AEs.
		23	What analysis was performed: frequencies, percentages, differences and 95% confidence intervals, significance tests, other? (Select all that apply)	

For peer review only - http://bmjopen.bmj.com/site/about/guidelines.xhtml

	Details of how AEs were summarised and presented - continuous outcomes	24	Were continuous outcomes dichotomised: Yes for all, yes for some, no or not applicable?	This includes measures that will have been captured as continuous and then dichotomised for example blood levels, blood pressure etc.
		25	If continuous outcomes were analysed as continuous what analysis was performed: differences in measures of central tendency, significance tests, other? (Select all that apply)	
	Details of how AEs were summarised and presented	26	Were signal detection methods used?	
		27	Were any graphical summaries of AEs presented?	
		28	Were severity ratings given: Yes for all, yes for some, no or not applicable?	
		29	Were numbers of serious events presented: Yes by treatment arm, yes overall, no or not applicable?	If death is reported as part of the efficacy outcome it is not enough to constitute reporting serious events.
		30	Were serious events coded as treatment related: Yes for all, yes for some, no or not applicable?	
		31	Provided information on the duration of events?	This refers to the length of the actual AE i.e. how long did it last.
		32	Provided information on the timing of events?	This refers to the time of onset of the AE.
		33	Accounted for multiplicity of statistical tests?	
		34	Referenced CONSORT extension for harms?	

For peer review only - http://bmjopen.bmj.com/site/about/guidelines.xhtml

Table A2: Stopping criteria for safety

Study	Main article text	Appendix text
Myles et al.[53]	"O'Brien–Fleming stopping boundaries were used to assess efficacy, and *a less stringent boundary was used to assess harm*."	
Billings et al.[54]	"The data and safety monitoring board (DSMB) reviewed patient recruitment practices, safety reporting, and data quality after 30 patients completed the study; performed an interim analysis after 277 patients … had completed the study to assess *safety of the intervention*; and performed a second interim analysis after 546 patients … had completed the study to assess the safety, efficacy, and futility of the intervention. The DSMB made recommendations based on qualitative assessments of the safety, efficacy, and futility of the intervention…"	"*Suspend enrolment in any study arm … due to safety concerns based on study intervention. Safety concerns include*:<br>• Increase in in-hospital all-cause mortality in subjects randomized to A or B such that the DSMB deems the increase is excessive compared to A or B.<br>• Increased treatment toxicity in either treatment group deemed excessive. Toxicity is defined as moderate or severe myalgias.<br>• Increased severity of adverse events deemed "Probably Related" or "Possibly Related" to study intervention in either treatment group. Itemized adverse event reports separated by treatment will be provided.<br>• Increased AKI incidence in either treatment group deemed excessive. • Increased incidence of stroke or hemodialysis requirement in either group (secondary endpoints) deemed excessive."
Beardsley et al.[55]	"An independent data and safety monitoring committee oversaw trial safety and analyzed unblinded data after every 50 deaths, according to its charter ..."	"The Haybittle-Peto boundary, requiring p<0.001 at interim analysis to consider stopping for efficacy, will be used as guidance. *A level of significance of 1% will be used as a guide for stopping the trial early because of a detected harm of dexamethasone*. In addition, the DMEC will receive conditional power curves to assess whether it remains realistic that the trial will demonstrate superiority of dexamethasone conditional on the data accrued up to the point of the interim analysis. Importantly, the DMEC recommendations will not be based purely on statistical tables but will also use clinical judgment."
Kor et al.[56]	"In addition to statistical criteria for significance, the study included a priori "go-no-go" definitions for recommending continuation to phase 3 study … Briefly, continuation to phase 3 would occur with a positive primary outcome finding along with an acceptable safety profile. An acceptable safety profile was defined as a serious adverse event profile for aspirin that was not statistically worse than placebo (95% CI for the relative risk of any serious adverse event covers the null value of relative risk = 1.0). The "no-go decision" was defined as early termination by the data and safety monitoring board for safety or unfavorable risk/benefit ratio. An indeterminate case in which there was a non–statistically significant effect but this effect was in a clinically meaningful direction was also defined."	Initiate Phase III Study: Demonstrated efficacy signal in addition to adequate safety profile Criteria: Early termination for benefit at interim analysis or p<0.08885 at final analysis (alpha=0.10 for study). *Serious adverse event profile of ASA not statistically worse than placebo (95% confidence interval for the relative risk of any SAE covers the null value of RR=1.0).*<br>Further Development Potentially Required: Weak efficacy signal Criteria: Primary endpoint did not achieve a priori level of significance but there were at least a general consistency of secondary endpoints indicating propensity for efficacy with a larger sample size and/or more specific primary endpoint.<br>Abandon Treatment Platform: Harm (in efficacy or safety endpoints) Criteria: Study terminated early per recommendation *by DSMB for safety and/or risk/benefit ratio concerns* (i.e., stop for futility, harm, unacceptable risk profile, etc.)

For peer review only - http://bmjopen.bmj.com/site/about/guidelines.xhtml

Nichol et al.[57]	We used a group sequential statistical approach to do two equally spaced pre-planned interim analyses (at 33% and 67% of total recruitment) to assess accumulated safety data (differential proportions of deep venous thrombosis and total mortality). This *approach was chosen to provide for early stopping for probable harm* or strong evidence of benefit. We applied the Haybittle-Peto criterion (|Zk|≥3) for early stopping at these analyses.	

For peer review only

For peer review only - http://bmjopen.bmj.com/site/about/guidelines.xhtml

Table A3: Selection criteria used to select AEs presented in the main journal report

Selection criteria	n	%
All AEs presented	20	10.87
AEs in greater than x% in any group	10	5.43
AEs in greater than x% in treatment group	4	2.17
AEs in greater than x% in all patients	1	0.54
Most common (no criteria specified)	9	4.89
Predefined AEs	26	14.13
SAEs	15	8.15
AEs leading to study drug discontinuation/interruption	3	1.63
Treatment related AEs	5	2.72
Grade 3>= events	9	4.89
AEs in greater than x% in any group & predefined/special interest AEs	4	2.17
AEs in greater than x% in any group & frequency between groups differed by more than y% & predefined/special interest AEs	1	0.54
AEs in greater than x% in all patients & predefined/special interest AEs	3	1.63
AEs in greater than x% in treatment group & AEs of special interest	2	1.09
AEs in greater than x% in any group & all SAEs	2	1.09
AEs in greater than x% in all patients & all SAEs	1	0.54
AEs in greater than x% in any group & SAEs related to treatment	1	0.54
Most common (no criteria specified) & predefined/special interest AEs	3	1.63
Most common (no criteria specified) & all SAEs	4	2.17
Most common (no criteria specified) & all SAEs & AEs leading to study drug discontinuation/interruption	1	0.54
Most common (no criteria specified) & treatment related SAEs	1	0.54
AEs where frequency between groups differed by more than y% & all SAEs	1	0.54
AEs of special interest	6	3.26
Grade >=3 AEs in greater than x% of patients	1	0.54
Grade >=3 AEs in greater than x% in intervention & y% in control	1	0.54
Most common (no criteria specified) grade 3>= AEs	1	0.54
Most common SAEs (no criteria specified)	1	0.54
SAEs & AE of special interest	1	0.54
Treatment related AEs in greater than x% of patients	1	0.54
Treatment related AEs in greater than x% in any group	1	0.54
AEs in greater than x% in treatment group & SAEs	1	0.54
AEs in greater than x% in treatment group & SAEs & predefined AEs	2	1.09
AEs in greater than x% in any group & significantly different & SAEs	1	0.54
AEs in greater than x% in any group & treatment related AEs/SAEs	2	1.09
AEs in greater than x% in treatment group & treatment related AEs & SAEs	1	0.54
AEs in greater than x% in treatment group & treatment related AEs in greater than y% in all patients	1	0.54
AEs in greater than x% in any group & Grade 3>= events	1	0.54
AEs in greater than x% in all patients & Grade 3>= events	1	0.54
AEs in greater than x% in all patients & Grade 2>= treatment related AEs	1	0.54
AEs in greater than x% in any group & Grade 3>= events in greater than y% in any group	1	0.54
AEs in greater than x% in any group & SAEs in treatment group	1	0.54
AEs in greater than x% in any group & AEs of special interest & most common (no criteria specified) AEs leading to treatment discontinuation/interruption & predefined AEs	1	0.54
AEs in greater than x% in any group, AEs of special interest in greater than y% in treatment group & treatment related deaths	1	0.54

For peer review only

AEs in greater than x% in treatment group & SAEs in greater than y% in any group	1	0.54
AEs and SAEs occurring more often in treatment group than control	1	0.54
AEs in greater than x% in treatment group & occurred more often in treatment group than control & predefined/special interest AEs	1	0.54
AEs in greater than x% in any group & frequency between groups differed by more than y%, SAEs in greater than z% in any group & all grade >=3 AEs	1	0.54
AEs in greater than x% patients & more than y% difference between treatment groups & AEs leading to treatment discontinuation/interruption & most common SAEs (no criteria specified) & death	1	0.54
Predefined AEs, AEs leading to hospitalisation/death/study drug discontinuation/interruption & SUSARS	2	1.09
Some form of overall summary	6	3.26
Not specified how selected	6	3.26
Not summarised in main paper	11	5.98

For peer review only

Table A4: Selection criteria used to select AEs presented in the appendix

Selection criteria	n	%
All AEs	18	9.78
SAEs	18	9.78
All AEs & SAEs	4	2.17
AEs in greater than x% in any group	7	3.8
AEs in greater than x% in treatment group	2	1.09
AEs in greater than x% in all patients	1	0.54
AEs in greater than x% in any group & all SAEs	2	1.09
AEs in greater than x% in treatment group & all SAEs	1	0.54
AEs in greater than x% in all patients & all SAEs	3	1.63
AEs in greater than x% in treatment group & all SAEs	1	0.54
AEs in greater than x% in treatment group & greater than in control group & all SAEs	1	0.54
SAEs in greater than x% in any group	1	0.54
AEs in greater than x% in any group & SAEs in greater than y% in any group	1	0.54
AEs in greater than x% in any group & AEs of special interest	2	1.09
Treatment related AEs	5	2.72
Treatment related AEs in greater than x% in any group	2	1.09
Grade 3>= events	2	1.09
Predefined AEs	8	4.35
AEs of special interest	1	0.54
AEs leading to study drug discontinuation/interruption	2	1.09
AEs leading to study drug discontinuation & SAEs	1	0.54
Grade 3>= events leading to study drug discontinuation & grade 3>= laboratory results	1	0.54
Treatment related AEs & AEs leading to study drug discontinuation	1	0.54
AEs in greater than x% in all patients leading to treatment discontinuations, SAEs in greater than x% in any group, serious predefined/special interest AEs and clinically significant laboratory results	1	0.54
AEs in greater than x% in any group, treatment related AEs in greater than x% in any group, treatment related SAEs and select AEs	1	0.54
Clinical laboratory data	1	0.54
Predefined AEs, AEs leading to hospitalisation/death/study drug discontinuation/interruption & SUSARS	3	1.63
Deaths	2	1.09
Some form of overall summary	5	2.72
Not specified how selected	2	1.09
Not summarised in the appendix	84	45.65

For peer review only

Table A5: Population used for AE analysis

**Analysis population**	**n**	**%**
Those that took at least a single dose	75	40.76
All randomised	54	29.35
Randomised and not withdrawn/ineligible	19	10.33
Not specified	17	9.24
Not applicable	3	1.63
Took a single dose and underwent AE/toxicity assessment	3	1.63
Active treatment groups	2	1.09
Completed treatment and assessed for primary outcome	2	1.09
Other	2	1.09
Patients who treatment was at least attempted on	1	0.54
Intention-to-treat population	1	0.54
Randomised and assessed for primary outcome	1	0.54
Randomised and attended at least on follow-up visit	1	0.54
Randomised and remained in follow-up	1	0.54
Randomised and underwent AE/toxicity assessment	1	0.54
Randomised, eligible and received at least a single dose	1	0.54

For peer review only

For peer review only - http://bmjopen.bmj.com/site/about/guidelines.xhtml

Reporting checklist for systematic review and meta-analysis.

Based on the PRISMA guidelines.

Instructions to authors

Complete this checklist by entering the page numbers from your manuscript where readers will find each of the items listed below.

Your article may not currently address all the items on the checklist. Please modify your text to include the missing information. If you are certain that an item does not apply, please write "n/a" and provide a short explanation.

Upload your completed checklist as an extra file when you submit to a journal.

In your methods section, say that you used the PRISMA reporting guidelines, and cite them as:

Moher D, Liberati A, Tetzlaff J, Altman DG, The PRISMA Group. Preferred Reporting Items for Systematic Reviews and Meta-Analyses: The PRISMA Statement

		Reporting Item	Page Number
	#1	Identify the report as a systematic review, meta-analysis, or both.	1
Structured summary	#2	Provide a structured summary including, as applicable: background; objectives; data sources; study eligibility criteria, participants, and interventions; study appraisal and synthesis methods; results; limitations; conclusions and implications of key findings; systematic review registration number	2
Rationale	#3	Describe the rationale for the review in the context of what is already known.	5-6
Objectives	#4	Provide an explicit statement of questions being addressed with reference to participants, interventions, comparisons, outcomes, and study design (PICOS).	6
Protocol and	#5	Indicate if a review protocol exists, if and	n/a - review protocol was not

For peer review only

registration		where it can be accessed (e.g., Web address) and, if available, provide registration information including the registration number.	published
rEEligibility criteria	#6	Specify study characteristics (e.g., PICOS, length of follow-up) and report characteristics (e.g., years considered, language, publication status) used as criteria for eligibility, giving rational	6-7
Information sources	#7	Describe all information sources in the search (e.g., databases with dates of coverage, contact with study authors to identify additional studies) and date last searched.	6
Search	#8	Present full electronic search strategy for at least one database, including any limits used, such that it could be repeated.	n/a - we manually searched the electronic contents table of the journals for reports of original RCTs
Study selection	#9	State the process for selecting studies (i.e., for screening, for determining eligibility, for inclusion in the systematic review, and, if applicable, for inclusion in the meta-analysis).	6
Data collection process	#10	Describe the method of data extraction from reports (e.g., piloted forms, independently by two reviewers) and any processes for obtaining and confirming data from investigators.	7
Data items	#11	List and define all variables for which data were sought (e.g., PICOS, funding sources), and any assumptions and simplifications made.	7
Risk of bias in individual studies	#12	Describe methods used for assessing risk of bias in individual studies (including specification of whether this was done at the study or outcome level, or both), and how this information is to be used in any data synthesis.	n/a – the review was to identify current practice, we did not look at and synthesize the actual results of individual studies and as such this assessment was not relevant

For peer review only

Summary measures	#13	State the principal summary measures (e.g., risk ratio, difference in means).	8
Planned methods of analyis	#14	Describe the methods of handling data and combining results of studies, if done, including measures of consistency (e.g., I2) for each meta-analysis.	8
Risk of bias across studies	#15	Specify any assessment of risk of bias that may affect the cumulative evidence (e.g., publication bias, selective reporting within studies).	n/a – please see item #12
Additional analyses	#16	Describe methods of additional analyses (e.g., sensitivity or subgroup analyses, meta-regression), if done, indicating which were pre-specified.	n/a – no such analysis was performed
Study selection	#17	Give numbers of studies screened, assessed for eligibility, and included in the review, with reasons for exclusions at each stage, ideally with a flow diagram.	n/a – manual search resulted in only eligible articles being downloaded
Study characteristics	#18	For each study, present characteristics for which data were extracted (e.g., study size, PICOS, follow-up period) and provide the citation.	8
Risk of bias within studies	#19	Present data on risk of bias of each study and, if available, any outcome-level assessment (see Item 12).	n/a – please see item #12
Results of individual studies	#20	For all outcomes considered (benefits and harms), present, for each study: (a) simple summary data for each intervention group and (b) effect estimates and confidence intervals, ideally with a forest plot.	n/a – only simple descriptive statistics are presented to describe current practice
Synthesis of results	#21	Present the main results of the review. If meta-analyses are done, include for each, confidence intervals and measures of consistency.	8-12

For peer review only

Risk of bias across studies	#22	Present results of any assessment of risk of bias across studies (see Item 15).	n/a – please see item #12
Additional analysis	#23	Give results of additional analyses, if done (e.g., sensitivity or subgroup analyses, meta-regression [see Item 16]).	n/a – please see item #16
Summary of Evidence	#24	Summarize the main findings, including the strength of evidence for each main outcome; consider their relevance to key groups (e.g., health care providers, users, and policy makers	13-18
Limitations	#25	Discuss limitations at study and outcome level (e.g., risk of bias), and at review level (e.g., incomplete retrieval of identified research, reporting bias).	18
Conclusions	#26	Provide a general interpretation of the results in the context of other evidence, and implications for future research.	18-19
Funding	#27	Describe sources of funding or other support (e.g., supply of data) for the systematic review; role of funders for the systematic review.	4

The PRISMA checklist is distributed under the terms of the Creative Commons Attribution License CC-BY. This checklist was completed on 29. May 2018 using http://www.goodreports.org/, a tool made by the EQUATOR Network in collaboration with Penelope.ai

For peer review only

BMJ Open

Analysis and reporting of adverse events in randomised controlled trials: a review

Journal:	*BMJ Open*
Manuscript ID	bmjopen-2018-024537.R1
Article Type:	Research
Date Submitted by the Author:	17-Oct-2018
Complete List of Authors:	Phillips, Rachel; Imperial College London, Faculty of Medicine, School of Public Health<br>Hazell, Lorna; Drug Safety Research Unit, Clinical Research; University of Portsmouth, Associate Dept. of Pharmacy and Biomedical Sciences<br>Sauzet, Odile; Universität Bielefeld,<br>Cornelius, Victoria; Imperial College London Faculty of Medicine, School of Public Health
**Primary Subject Heading**:	Research methods
Secondary Subject Heading:	Medical publishing and peer review
Keywords:	Randomised controlled trials, Harm data, Adverse drug reactions, Systematic review, Investigational drug, Adverse events < THERAPEUTICS

SCHOLARONE™
Manuscripts

Analysis and reporting of adverse events in randomised controlled trials: a review

Authors:

Rachel Phillips* - Imperial College London, London, United Kingdom

Lorna Hazell – Drug Safety Research Unit, Southampton, United Kingdom

Odile Sauzet – Bielefeld University, Bielefeld, Germany

Victoria Cornelius - Imperial College London, London, United Kingdom

* Corresponding author

Address: Imperial Clinical Trials Unit, Imperial College London, 1st Floor Stadium House, 68 Wood Lane, London, W12 7RH

Email: r.phillips@imperial.ac.uk

Telephone: 020 759 49356

Word Count: 4204

For peer review only

Abstract

Objective

To ascertain current approaches to the collection, reporting and analysis of adverse events (AEs) in randomised controlled trials (RCTs) with a primary efficacy outcome.

Design

A review of clinical trials of drug interventions from four high impact medical journals.

Data sources

Electronic contents table of the BMJ, the Journal of the American Medical Association, the Lancet, and the New England Journal of Medicine were searched for reports of original RCTs published between September 2015 and September 2016.

Methods

A pre-piloted checklist was used and single data extraction was performed by three reviewers with independent check of a randomly sampled subset to verify quality. We extracted data on collection methods, assessment of severity and causality, reporting criteria, analysis methods and presentation of AE data.

Results

For peer review only - http://bmjopen.bmj.com/site/about/guidelines.xhtml

We identified 184 eligible reports (BMJ n=3; JAMA n=38, Lancet n=62; and NEJM n=81). Sixty-two percent reported some form of spontaneous AE collection but only 29% included details of specific prompts used to ascertain AE data. Numbers that withdrew from the trial were well reported (80%), however only 35% of these reported whether withdrawals were due to AEs. Results presented and analysis performed was predominantly on 'patients with at least 1 event' with 84% of studies ignoring repeated events. Despite a lack of power to undertake formal hypothesis testing, 47% performed such tests for binary outcomes.

Conclusions

This review highlighted that the collection, reporting and analysis of AE data in clinical trials is inconsistent and RCTs as a source of safety data are underutilised. Areas to improve include reducing information loss when analysing at patient level and inappropriate practice of underpowered multiple hypothesis testing. Implementation of standard reporting practices could enable a more accurate synthesis of safety data and development of guidance for statistical methodology to assess causality of AEs could facilitate better statistical practice.

Keywords

Randomised controlled trials; adverse events; harm data; adverse drug reactions; review; investigational drug.

For peer review only - http://bmjopen.bmj.com/site/about/guidelines.xhtml

Article Summary

Strengths and Limitations of this study

1. This is the first review to examine and quantify AE analysis practice in RCTs published in high impact journals.
2. This review identifies weakness that need to be addressed as well as good practice that could be adopted.
3. Articles included in this review were published in four of the top ranked general medical journals therefore results are likely to be biased towards better practice.

For peer review only

For peer review only - http://bmjopen.bmj.com/site/about/guidelines.xhtml

INTRODUCTION

The methods to analyse and report outcomes to measure benefit from randomised controlled trials (RCTs) are well developed but this progress has not been matched for adverse event (AE) outcomes. An adverse event is defined as 'any untoward medical occurrence that may present during treatment with a pharmaceutical product but which does not necessarily have a causal relationship with this treatment'.[1] An adverse drug reaction (ADR) is defined as 'a response to a drug which is noxious and unintended …' where a causal relationship is 'at least a reasonable possibility'.[1, 2] RCTs provide an opportunity to compare rates of AEs between arms allowing causality to be evaluated. However current analysis and reporting practices are inadequate.

There are many challenges associated with analysing and reporting AEs in clinical trials. RCTs are typically designed to determine the efficacy of an intervention but are often underpowered to detect important differences in AEs between arms which may suggest an ADR. Often large numbers of AEs are reported during a study, sometimes exceeding the number of patients in the clinical trial. Performing hypothesis tests on these AEs would lead to issues of multiplicity, however any adjustment for multiplicity would make a 'finding untenable'.[3, 4] The use of hypothesis testing may result in the medicinal product being deemed unsafe and a trial being halted too early due to a chance imbalance, or conversely deemed safe and not stopped early enough resulting in more patients than necessary suffering an ADR.[3, 5, 6] Unlike efficacy outcomes which are well defined and restricted in number at the planning stage of a RCT, we collect numerous, undefined AEs in RCTs. Furthermore, AE collection

For peer review only

requires additional information to be obtained on factors such as severity, timing and duration, number of occurrences and outcome, which for our efficacy outcomes would have all been predefined.

Previous studies have examined the methods for AE collection and presentation only, and highlighted the inadequacies in AE reporting in journal articles.[7-16] In 2004 the Consolidated Standards of Reporting Trials (CONSORT) Group produced an extension to their guidelines for reporting trial results to cover the reporting of harms, however implementation of these guidelines has been shown to be poor.[10, 14-17] Recently a joint pharmaceutical/journal collaboration published practical guidance and examples on what should be reported in journal articles and how it should be displayed to ensure transparency and aid clinical interpretation. They promote the use of clinical judgement in reporting rather than mandatory guidance.[18] Whilst this work has been undertaken there remains uncertainty about practice for reporting and presenting AE data, and in addition the analysis practice for AEs remains a neglected area for review.

The aim of this review was to evaluate current practice for collection, reporting and analysis of AEs in RCTs where the primary outcome was efficacy. The aim being to identify and promote any areas of good practice, whilst highlighting any areas for improvement.

METHODS

Search strategy

For peer review only - http://bmjopen.bmj.com/site/about/guidelines.xhtml

The top four general medical journals as ranked by impact factors that publish clinical trials of drug interventions were selected: The BMJ (Impact Factor 20.79), the Journal of the American Medical Association (JAMA, IF 44.41), the Lancet (IF 47.83), and the New England Journal of Medicine (NEJM, IF 72.41). Impact factors quoted are from 2016 to reflect the time period from which the articles were drawn. One reviewer manually searched the electronic contents table of the journals for reports of original RCTs published between September 2015 and September 2016, inclusive. Any queries regarding eligibility were reviewed and discussed with a second reviewer.

Selection criteria

The inclusion criteria were phase II-IV RCTs of drug interventions where the primary outcome was efficacy of the intervention. We did not restrict according to number of treatment arms and included both parallel and cluster RCTs. We excluded cross-over RCTs, RCTs with adaptive randomisation, observational studies, case reports, editorials and letters. We also excluded RCTs where the intervention was not a drug product (i.e. not classified as a clinical trial of an investigational medicinal product (CTIMP)). As the study aimed to assess how authors report and analyse AEs in studies where the primary outcome was efficacy, trials that were specifically designed to investigate safety as a primary outcome were not included.

Data extraction

For peer review only - http://bmjopen.bmj.com/site/about/guidelines.xhtml

Potentially eligible articles were identified based on titles and abstracts and the full text of these studies were retrieved. Supplementary material was also reviewed if readers were referred here from the main article for further results. Supplementary Table A1 lists all data items captured with guidance given to the reviewers for extraction. We focused on the following areas: how AE data was collected (mode of collection, timing) and defined (coding, attribution); how AEs were assessed in terms of severity of the event or relatedness to the medical intervention; if there was any planned AE analysis (final and interim monitoring plans and analysis populations); how events were selected for inclusion in the journal article; how summary event information was presented in the journal article and how AEs were analysed.[11]

The items to be extracted were based on the work by Cornelius et al. and the CONSORT Harms extension with new items added to capture more specific information on analysis practices.[11, 17] A data extraction sheet was piloted and then single data extraction was performed by three reviewers (RP, VC and LH) with 10% independent check of a randomly sampled subset to verify quality. Queries were also informally discussed between reviewers on an ongoing basis. Where specific items were flagged for poor agreement these were re-extracted. Any queries during data extraction were shared and disagreements between reviewers were resolved through discussion.

Data analysis

The proportion of trials reporting each item, 3-4 and 8-34 in supplementary Table A1 were calculated and summary statistics (median and ranges) were calculated for items 5-7. All analyses were performed

For peer review only - http://bmjopen.bmj.com/site/about/guidelines.xhtml

in Stata version 15.[19] A risk of bias assessment was not undertaken as this study aimed to describe best practice and not evaluate outcomes.

Patient and public involvement

This review forms part of a wider research project that was developed with input from a range of patient representatives. There were no study participants directly involved in this review but the original proposal and patient and public involvement (PPI) strategy were reviewed by service user representatives (with experience as clinical trial participants and PPI advisors) who provided advice specifically with regard to communication and dissemination to patient and public groups.

RESULTS

Data extraction

A total of 585 items were extracted twice across all three reviewers to check the quality of the data extraction. A total of 95 discrepancies were identified. This gave agreement of 84%. During this independent check several items were flagged for potential poor agreement. These items were 100% independently extracted by one author and verified. The items were: study duration; the AE collection method; timing of collection; how binary harm outcomes were summarised; whether continuous outcomes were dichotomised; if continuous outcomes were left as continuous how they were analysed.

For peer review only - http://bmjopen.bmj.com/site/about/guidelines.xhtml

Study characteristics

The search identified 184 eligible trial reports (BMJ n=3; JAMA n=38, Lancet n=62; and NEJM n=81) in which a total of 496911 participants were randomised with a median of 556 participants per trial (range 30, 205513; interquartile range (IQR) 281, 1704). The median trial follow-up was 52 weeks (range 48 hours to 10 years; IQR 24, 104 weeks) and 93% were multi-centre trials. Fifty-percent of studies had an active comparator and over 50% of trials received some element of industry funding (Table 1).

Table 1: Characteristics of included studies

**Characteristic**	N=184		
	**Median**	**(IQR)**	**min, max**
**Sample size**	556	(281, 1704)	30, 205513
**Centres**[a]	35	(12, 100)	1, 1368
**Trial duration (weeks)**[b]	52	(24, 104)	0.3, 521
	**n**	**%**	
**Journal**			
**BMJ**	3	1.6	
**JAMA**	81	44.0	
**Lancet**	38	20.7	
**NEJM**	62	33.7	
**Funded by**			
**Public**	70	38.3	
**Industry**	80	43.7	
**Both**	33	18.0	
**Centre**			
**Single-centre**	12	7.0	
**Multi-centre**	161	93.0	
**Control**			
**Placebo**	95	51.6	
**Active**	80	43.5	
**Both**	8	4.4	
**Neither**[c]	1	0.5	

For peer review only - http://bmjopen.bmj.com/site/about/guidelines.xhtml

Abbreviations: *IQR = Inter-quartile range; min = minimum; and max = maximum*

[a]11 reports did not specify the number of centres

[b]2 reports did not specify trial duration

[c]One trial compared interventional drug to behavioural change intervention

Collection and assessment methods

Sixty-two percent (n=114) of reports made reference to some form of passive (e.g. spontaneously reported by patients) AE monitoring or collection methods. Of these only 46.5% (53/114) or 29% of total reports included specific details (prompts e.g. questions about specific events or AEs in general, questionnaires, or diaries) regarding these collection methods (Table 2, examples 1-2).[20, 21] The timing of collection was well documented (91%, 48 out of 53 reports) in the reports that included specific details about the prompts used to collect AEs. Although specific details on clinical examinations (e.g. vital signs and blood pressure) and laboratory tests were not widely reported (only 57% of reports (95 out of 166 reports with clinical examinations and/or laboratory results presented) included details on the timing of such assessments) it was often clear from the results presented that participants had undergone these assessments (83% and 79% of studies reported clinical and laboratory results respectively) (Table 3).

Table 2: Examples of reporting practice in reviewed articles

Example no.	Study	Example practice	Example
1	Litonjua et al.[20]	Description of AE collection method	*"Study staff met with pregnant women monthly to administer a brief health questionnaire, assess medication use, and monitor for complications (via the questionnaire and medical record review)… After delivery, children were monitored by telephone every 3 months and in-person annually for 3 years, during which time infants' health, respiratory symptoms, and medications were assessed"*

For peer review only

For peer review only - http://bmjopen.bmj.com/site/about/guidelines.xhtml

2	Miller et al.[21]	Description of AE collection method	*"Safety evaluations included physical examinations, assessment of vital signs, clinical laboratory tests, and reporting of adverse events at each study visit"*
3	Libman et al.[22]	Description of planned AE analysis	*"The proportions of participants experiencing any adverse event, any related adverse event, any gastrointestinal event, any event other than a gastrointestinal event, at least 1 severe hypoglycaemic event, and at least 1 diabetic ketoacidosis event in each treatment group were compared using the Fisher exact test. The number of adverse events, new adverse events, serious adverse events, and non-serious adverse events were compared between groups using a Wilcoxon rank sumtest."*
4	Gross et al.[23]	Description of planned AE analysis	*"Safety analyses and secondary efficacy analyses used binomial regression, analysis of covariance, or the marginal Cox proportional hazards model as appropriate"*

For peer review only

For peer review only - http://bmjopen.bmj.com/site/about/guidelines.xhtml

Table 3: Collection, assessment and analysis methods reported by studies

Section	Component	Data item	N=184	
**Collection**			n	%
	**How was AE/harm information collected?**			
		Passive collection	114	62.0
		Prompted collection (n=114)	53	46.5
		No method of collection reported	70	38.0
	**Did they undertake proactive screening?**			
		Clinical examinations	153	83.2
		Laboratory tests	146	79.4
	**Timing of prompted collection specified** (n=53)		48	90.6
	**Timing of active collection specified** (n=166)		95	57.2
	**Which, if any, dictionary was used to code AE data?**			
		CTCAE	18	9.8
		MedDRA	43	23.4
		CTCAE and MedDRA	1	0.5
		DAIDS	2	1.1
		ICD-10	1	0.5
		Researcher defined	2	1.1
		Other	3	1.6
		No dictionary reported	114	62.0
**Assessment**				
	**Who assigned attribution to study drug?**			
		Blinded assessor	9	4.9
		Unblinded assessor	7	3.8
		Both	1	0.5
		Not specified	164	89.1
		Not applicable[a]	3	1.6
**Analysis**				
	**Was any analysis for AEs specified in the methods section?**			
		Yes	57	31.0
	**Was a population for AE analysis specified?**			
		Yes	82	44.6
	**Was there a planned interim analysis with stopping criteria?**			
		No	138	75.0
		Yes for efficacy	24	13.0
		Yes for efficacy & futility	11	6.0
		Yes for efficacy & safety	3	1.6
		Yes for efficacy, futility & safety	2	1.1
		Yes but no other details given	6	3.3

Abbreviations: *AE = Adverse event; CTCAE = Common Terminology Criteria for Adverse Events; MedDRA = Medical Dictionary for Regulatory Activities; DAIDS = The Division of AIDS; and ICD-10 = International Classification of Diseases 10th revision.*

For peer review only

For peer review only - http://bmjopen.bmj.com/site/about/guidelines.xhtml

NOTE: Denominator specified in item column if it differs from total sample

[a]3 reports made no reference to AE data throughout the article

Prespecified analysis

Thirty-one percent of reports provided information on the planned analysis for AEs in the statistical analysis section of the paper and 45% pre-specified a safety population (Table 2, examples 3-4 and Table 3).[22, 23] A quarter of trials reported planned interim analysis with stopping criteria (Table 3), five (2.7%) of which included specific criteria on stopping for a harmful event (Supplement Table A2 [24-28]).

Selection of AEs and reporting practices

Two reports only made generic statements regarding AE data: "*there were no significant adverse events related to the procedure*" and "*no excess in mortality or major adverse events were found…*". Three reports made no mention of AEs throughout the manuscript.[29-33]

Twenty-four (13%) trials only provided a summary of the number of AEs or serious AEs rather than listing the actual AEs that occurred. For example "*Six serious adverse events occurred in the acetaminophen group and 12 in the ibuprofen group.*"[34] Of these 24 trials, 10 did provide specific details of the types of events in an appendix. This means 8% of trials either did not report AEs or only included a summary (Table 4).

For peer review only - http://bmjopen.bmj.com/site/about/guidelines.xhtml

Table 4: Summaries of results presented by studies

Component	Data item	N=184 n	%
**What was reported in the manuscript?**			
	Actual AE terms	73	39.7
	Summaries of AE type (e.g. AE, SAE)	24	13.0
	Both	80	43.5
	Neither	7	3.8
**What was reported in the appendix?**			
	Actual AE terms	76	41.3
	Summaries of AE type (e.g. AE, SAE)	7	3.8
	Both	22	12.0
	Neither	3	1.6
	Not applicable[a]	76	41.3
**Which population was the AE analysis performed on?**			
	All randomised	54	29.4
	Those that took at least a single dose	75	40.8
	Other	35	19.0
	Not specified	17	9.2
	Not applicable[b]	3	1.6
**Were drop-outs/withdrawals reported?**			
	No	33	17.9
	Yes by treatment arm	144	78.3
	Yes overall	2	1.1
	Not applicable[c]	5	2.7
**Were withdrawals due to AEs reported?** (n=146)			
	No	89	61.0
	Yes	51	34.9
	Not applicable[d]	6	4.1
**Were specific AEs causing withdrawals reported?** (n=51)			
	No	39	76.5
	Yes	12	23.5
**How were binary AE outcomes summarised by arm?**			
	Not summarised[e]	6	3.3
	Number of people with an event	154	83.7
	Number of events	11	6.0
	Both	12	6.5
	Unclear	1	0.5
**Were frequencies of AEs reported by arm?**			
	No	5	2.7
	Yes for some	13	7.1
	Yes for all	160	87.0
	Not applicable[e]	6	3.3

For peer review only - http://bmjopen.bmj.com/site/about/guidelines.xhtml

**Were percentages of AEs reported by arm?**			
	No	18	9.8
	Yes for some	25	13.6
	Yes for all	135	73.4
	Not applicable[e]	6	3.3
**Were between arm differences and 95% CI of AEs reported?**			
	No	141	76.6
	Yes for some	18	9.8
	Yes for all	19	10.3
	Not applicable[e]	6	3.3
**Were statistical significance tests between arms on AEs reported?**			
	No	92	50.0
	Yes for some	31	16.9
	Yes for all	55	29.9
	Not applicable[e]	6	3.3
**Were continuous AEs outcomes dichotomised for summaries?**			
	No	10	5.4
	Yes for some	28	15.2
	Yes for all	108	58.7
	Not applicable	38	20.7
**If continuous outcomes were left as continuous what between arm analyses was performed?** (n=38)			
**Differences in measures of central tendency estimated with 95% CI**			
	No	23	60.5
	Yes for some	1	2.6
	Yes for all	14	36.8
**Between arm hypothesis tests performed**			
	No	12	31.6
	Yes for some	2	5.3
	Yes for all	24	63.2
**Were any 'signal detection' approaches used?**			
	No	184	100.0
	Yes	0	0.0
**Were there any graphical presentations of AE outcomes?**			
	No	162	88.0
	Yes	22	12.0
**Were summaries of severity rating of AEs reported?**			
	No	103	56.0
	Yes for some	41	22.3
	Yes for all	35	19.0
	Not applicable[f]	5	2.7
**Were number of serious AEs reported?**			
	No	44	23.9
	Yes overall	2	1.1

For peer review only - http://bmjopen.bmj.com/site/about/guidelines.xhtml

	Yes by treatment arm	132	71.7
	Not applicable[g]	6	3.3
**For serious AEs was relatedness given?** (n=134)			
	No	77	57.5
	Yes for some	18	13.4
	Yes for all	38	28.4
	Yes overall	1	0.8
**Were there any AEs where information on duration of events was reported?**			
	No	175	95.1
	Yes	9	4.9
**Were there any AEs where information on the time of occurrence of events was reported?**			
	No	132	71.7
	Yes	52	28.3
**If any significance tests were performed on AEs was multiplicity of events accounted for?**			
	No	81	44.0
	Yes	3	1.6
	Not applicable	100	54.4
**Did the report reference the CONSORT extension to harms**			
	No	184	100.0
	Yes	0	0.0

Abbreviations: *AE = Adverse Event; SAE = Serious Adverse Event; CI = Confidence Interval; and CONSORT = Consolidated Standards for Reporting Trials.*

[a] Make no reference to the appendix

[b] 3 reports made no reference to AE data throughout the article

[c] 5 reports indicate no withdrawals

[d] 6 reports specify the number of withdrawals and reasons but none of the reasons are related to AEs

[e] This includes 3 reports with no AE data (as per footnote [b]), 2 reports that provide generic statements regarding AE data and 1 report that only reported continuous outcomes

[f] This includes 3 reports with no AE data and 2 reports that provide generic statements regarding AE data (as per footnote [e])

[g] 6 papers specifically state that no serious adverse events occurred

Eighty-nine percent of trials reported a subset of all the AEs they collected. How AEs are 'selected' for inclusion in the article was not consistent or clear, and in 3% of studies it was impossible to discern how the authors had selected the AEs they presented for inclusion. Twenty-six percent of reports selected events based on a frequency threshold e.g. events experienced by greater than x% in any group; 9% of reports used a measure of severity to select events e.g. AEs of grade 3 or higher; 23% of reports included events based on seriousness; and 8% included AEs based on relatedness to treatment (percentages are

For peer review only - http://bmjopen.bmj.com/site/about/guidelines.xhtml

For peer review only

not independent as the majority of reports used several different criteria for selection). Supplementary Tables A3 and A4 provide full details of selection criteria used.

We found that 41% of trials analysed AEs in participants that received at least one dose, 29% of trials used all randomised participants and 9% did not specify the analysis population (Table 4). Further details on analysis populations used are given in supplementary Table A5.

Nearly 80% of trials reported the number of participants who withdrew from the trial; of these 35% (51 of 146 reports) reported whether the withdrawals were due to AEs and of these 24% (12 of 51 reports) reported the actual events that caused withdrawals. Results presented and analysis performed was predominantly on 'patients with at least 1 event' with 84% of reports providing no information on the number of events occurring. An example of how to incorporate information on number of events is presented in [35]. Forty-one percent of trials reported information on the severity of AEs. Five percent of trials include a report of at least one event with duration, but presenting such data is limited in the main report. The trials that did present this information did so in a variety of ways. For example incorporating the information into the AE table with summary statistics such as the mean duration of certain events or presenting it for a subgroup of events in the footnotes of AE tables e.g. "*One event of non-serious squamous cell carcinoma (day 210, resolved on day 215; adalimumab treatment was not interrupted).*"[36-38] Twenty-eight percent of reports included information on the timing of AEs (Table 4).

Serious adverse events were typically well documented (73%) and six reports (3%) explicitly stated that no serious events had occurred. However for forty-four reports (24%) it was not possible to discern if no

For peer review only - http://bmjopen.bmj.com/site/about/guidelines.xhtml

serious events had occurred or whether they were simply omitted from the report. Forty-two percent (57 of 134 reports) of reports included details on whether the events had been classified as related to the intervention (Table 4).

Analysis of AE outcomes

Binary

The majority of trials summarised binary outcomes using frequencies (94%) and percentages (87%). Despite a lack of power to undertake formal hypothesis testing, 47% reported p-values for binary outcomes. For example "*There were no between-group differences in the rate of patients with at least 1 adverse event (16.7% [14 patients] in the clopidogrel group vs 21.8% [19 patients] in the placebo group; difference, −5.2% [95% CI, −17% to 6.6%]; P = .44).*" However with a total safety population of 171 such a test would have only had 13% power to detect such a difference and was therefore substantially underpowered. The conclusion that "*No significant increase in adverse events was observed*" makes no reference to the 95% confidence interval presented which indicates that the findings were in fact compatible with a 17% decrease in experiencing at least on AE as well as a near 7% increase.[39]

Continuous

There was a pervasive practise (59%) of categorising continuous clinical and laboratory outcomes. Of the trials that did not dichotomise continuous AE data nearly 70% performed some form of statistical significance testing (Table 4). Whilst continuous outcomes do not suffer to the same degree regarding

For peer review only - http://bmjopen.bmj.com/site/about/guidelines.xhtml

lack of power, multiple testing is still a problem, however no multiplicity corrections for continuous outcomes were performed.

Of the trials that performed statistical significance testing on AE data, only three made an adjustment for multiplicity of tests (all three on dichotomised outcomes).[36, 40, 41] Two of which used a Bonferroni correction and adjusted for the number of pairwise comparisons between each of the treatment groups for each individual event rather than the total number of significance tests performed. As such both analyses would have still been effected by issues of multiple testing.

Twelve percent of reports used graphs to illustrate AE data (Table 4). The CONSORT extension highlighted the value of graphs for summarising such data, especially for conveying information on time-to-event outcomes.[42] An example of such a plot is included in the supplement of reference [43] (eFigure 2).

We assessed any reference to the CONSORT Harm extension and found that none of the included studies mentioned it. Of the four journals included in the review, the Lancet was the only journal that made specific reference to the harm extension in their guidelines to authors.

DISCUSSION

The safety profile of a medicinal product is established through evidence collected from several sources including clinical trials, observational studies and spontaneous reports.[44] The advantage of clinical trial

For peer review only - http://bmjopen.bmj.com/site/about/guidelines.xhtml

data is that these provide a controlled comparison of the rate of AEs allowing causality to be evaluated but have the disadvantage that the sample size is often not large enough to detect rare ADRs.

To ensure that a useful and comprehensive picture of the safety profile is provided to all relevant parties clear reporting of AEs from clinical trials is required. Current research has shown the quality of reporting is substandard.[7-16] The aim of this study was to review current practice across four leading medical journals for AE collection, analysis and reporting practices, highlighting any areas for improvement and examples of good practice.

Collection and assessment methods

The CONSORT extension to harm was developed with the aim to improve reporting of safety data in RCTs.[42] None of the included studies referenced the CONSTORT HARM extension and of the items in our review that are covered in CONSORT many were not well reported.[17] This suggests that the CONSORT extension is not being routinely adopted by authors to aid their reporting. Most journals now request that authors include a completed CONSORT checklist when they submit their article but we are not aware of any journal that request the CONSORT harm extension to also be submitted. Of the four journals in this review the Lancet is the only journal that makes specific reference to the harm extension in their guidelines to authors. The CONSORT statement contains a single item related to safety, item 19: 'all important harms or unintended effects in each group' should be reported.[42] This may explain why some items listed on the CONSORT extension for harm were reported by so few trials. The adoption of CONSORT harms by journals may support better reporting.

For peer review only - http://bmjopen.bmj.com/site/about/guidelines.xhtml

We found that the method of AE collection was poorly reported. This has important implications for the type and frequency of AEs reported with "passive collection resulting in fewer recorded AEs".[45, 46] Where the method was given the timing of collection was typically also reported and we would recommend continuation of this practice. The frequency of AE collection has further important implications on the number of events reported. More frequent assessment and longer follow-up will result in more AEs reported.[17] It is important to consider these factors when making conclusions about the safety profile.

The method of attribution between drug and AE was another area where reporting practice was inadequate. However the joint pharmaceutical/journal collaboration indicate that such attribution has 'limited value' given the 'inherent subjectivity in such attribution'.[18]

Prespecified analysis

We found that formal assessments of AEs regarding stopping for emerging ADRs utilising statistical rules was rare. Subjective assessments of overwhelming amounts of data could easily lead to potential signals of harm being missed. There could be benefits to incorporating more objective methods alongside clinical review to monitor AE information, both for interim analysis by data monitoring committees (DMCs) and final trial analysis to help better identify drug harm relationships. Graphical displays have gone some way towards aiding interpretation.[47-51]

Selection of AEs and reporting practices

For peer review only - http://bmjopen.bmj.com/site/about/guidelines.xhtml

Due to space constraints in journal reports AE information is often included in the appendix. Whilst we encourage use of appendices and supplementary material for including additional detail on AEs, we caution authors against depositing all AE data into such documents without attempting to present a summary of the AE profile in the main article. It is important that the main report strikes a balance between efficacy and harm therefore allowing a risk-benefit assessment to be made solely from the article.

The failure to report any information on AEs restricts interpretation and prevents a risk-benefit assessment. We identified two reports that made generic summaries of the overall safety profile and it was clear in both that there had been harmful effects however the authors did not include any further information. Three reports contained no information leaving readers uninformed as to any additional information these studies may provide on the safety profile. Ambiguous reporting prevents building an accurate picture of the safety profile. As such profiles are developed on accumulating evidence, it is important that each study report to the same standard and information is not wasted.

We found that the selection criteria used by authors to decide what AEs to include in the report were arbitrary and inconsistent. This will have important implications when synthesising data across studies to construct safety profiles. Authors would benefit from guidance to facilitate consistency but currently research in this area is lacking. Lineberry et al. recommended clinically relevant events that should always be reported (deaths, SAEs and events leading to discontinuation of intervention) and criteria that should be considered when deciding what other AEs to report e.g. interest based on the disease(s) under investigation, comorbidities of the study population, intervention mechanism, trial duration.[18]

For peer review only - http://bmjopen.bmj.com/site/about/guidelines.xhtml

For peer review only

Standard outcomes for a drug class would be one potential solution to avoid issues of inconsistency suggested by Cornelius et al.[11]

CONSORT recommend that AE analyses should be performed on the intention-to-treat (ITT) population to maintain the random assignment.[17] However it is clear from our review that this population label is not always appropriately and consistently applied. There is a tendency for studies to make modifications to the ITT population. Using the ITT or modified-ITT population is likely to underestimate the risk by inflating the denominator with participants who may have never received the study drug.[52] Such estimates are appropriate for health economic evaluations where estimates of the cost-effectiveness will inform policy level decisions regarding how to treat the population. However a more appropriate population for AE analysis to inform prescriber and patient decisions may be those that receive at least one dose. It is important that authors are clear about what they are estimating and how this affects their conclusions.

Proxy outcomes can be used as a measure of the impact of AEs on patients. Examples include the number of withdrawal due to any reason, withdrawals due to AEs, the number of events an individual experiences, the severity of the AE and the duration. A high proportion of trials reported withdrawal for any reason and this is likely to be as a result of the CONSORT recommendations.[42] The other outcomes were not frequently reported and increasing this could facilitate interpretation.[17] This information would permit better evaluation of the impact of AEs and the tolerability of the intervention to inform patients' and clinicians' treatment decisions. Reporting numbers that experience at least one event only and not providing information on repeated events masks valuable information that may be important to the patient and the cost-effectiveness evaluation. For example, chronic, repeated headaches over an

For peer review only - http://bmjopen.bmj.com/site/about/guidelines.xhtml

extended duration will have an important impact for patients compared to a single headache or headaches over a short duration but it is not possible to distinguish between these two scenarios when reported as ‘at least one event’.[18] Severity of events was also an important aspect that was often not differentiated. For example there would be different impact on patients’ quality-of-life with mild compared to severe nausea and which could lead to changes in dosing regimens. Displaying such information for all AEs in tables would soon become overwhelming and make interpretation difficult. Graphical approaches have been suggested as a solution to aid review. Examples of such a plots can be found in [53]. Online appendices and supplementary material provide more opportunity to include this important information.

For serious adverse events information on the time of likely onset can be useful information to inform patient monitoring plans. For example the documented risk of suicide and suicidal ideation within the first few weeks of starting anti-depressant allows patients and prescribers to remain alert and monitor closely for this period. Nearly a third of reports included such information and we would encourage authors to adopt this practice.

Analysis of AE outcomes

The majority of trials in this review included a balanced report of AEs alongside benefit. However many included generic statements regarding the safety profile such as ‘the intervention was well tolerated’ or ‘the intervention exhibited a good safety profile’ and these were frequently based on post-hoc statistical tests. Guidelines caution against such tests.[18] The results of which are difficult to interpret as a lack of

For peer review only - http://bmjopen.bmj.com/site/about/guidelines.xhtml

For peer review only

significance does not indicate that the intervention is safe and conversely multiple testing without adjustment will increase the number of significant differences due to chance.[54, 55]

Graphs are an efficient method to convey and interpret large amounts of data and can make it easier to flag potential safety signals.[50, 51, 53] Twenty-two studies included in the review used graphs to present AE data and an example of one such report is given in the supplementary eTable of [56].

Limitations of trials

Trials are a valuable source for high quality adverse event data but compared to observational studies have smaller sample size, follow-up periods and generalisability which restrict the ability to detect rare ADRs, ADRs with long latency and drug interactions in complex populations. The typical duration of a trial means there is often insufficient follow-up to fully characterise the safety profile as it provides limited information on long-term exposure. Stringent inclusion criteria restrict the population the intervention is assessed in and so limited information on drug-interactions is obtained.[5]

Limitations of this study

Articles included in this review were published in four of the top ranked medical journals therefore results are likely to be biased towards better findings than we would expect if we included all RCTs and are only for year 2015-2016. We also acknowledge that only completing 10% independent check of

For peer review only - http://bmjopen.bmj.com/site/about/guidelines.xhtml

extracted data would not have removed subjectivity from the data extraction but are happy that ongoing discussion between authors to clarify any queries would have kept this to a minimum. However this review characterises what those leading the field are doing and provides some examples of good practice that could be adopted.

Conclusions and recommendations for future work

RCTs are a valuable source of information establishing the safety profile of medicinal products. Our review has demonstrated that data is not currently being fully utilised. Analysis of AE data is frequently inappropriate and reports often provide insufficient and inconsistent information to allow a comprehensive summary of the safety profile to be established. RCTs that have been published over a recent period in examples of high impact general medicine journals are deficient.

This research has identified two areas that would benefit from future research. i) Improving the consistency of reporting important AE outcomes across trials to facilitate comparison and synthesis. This is in line with work from the COMET Initiative group (http://www.comet-initiative.org/). The development of CORE safety outcomes by drug class could be considered.[7] ii) Evaluation of methods to analyse AEs in RCTs.

For peer review only - http://bmjopen.bmj.com/site/about/guidelines.xhtml

COMPETING INTERESTS

None declared.

FUNDING

This research was supported by the NIHR grant number DRF-2017-10-131.

DATA SHARING STATEMENT

No additional data are available.

AUTHOR STATEMENT

RP conceived the idea for this review, conducted the search, carried out data extraction and analysis, and wrote the manuscript. VC conceived the idea for the review, performed data extraction and critical revision of the manuscript. LH performed data extraction and critical revision of the manuscript. OS performed critical revision of the manuscript.

For peer review only

REFERENCES

1. Edwards IR and Biriell C. Harmonisation in pharmacovigilance. *Drug safety* 1994; 10: 93-102. DOI: 10.2165/00002018-199410020-00001.
2. The International Council for Harmonisation of Technical Requirements for Pharmaceuticals for Human Use (ICH). ICH Harmonised Tripartite Guideline. E2A Clinical Safety Data Management: Definitions and Standards for Expedited Reporting. 1994.
3. Ma H, Ke C, Jiang Q, et al. Statistical Considerations on the Evaluation of Imbalances of Adverse Events in Randomized Clinical Trials. *Therapeutic Innovation and Regulatory Science* 2015; 49: 957-965. DOI: http://dx.doi.org/10.1177/2168479015587363.
4. Food and Drug Administration. *Attachment B: Clinical Safety Review of an NDA or BLA of the Good Review Practice. Clinical Reveiw Template (MAPP 6010.3 Rev. 1).* 2010.
5. Siddiqui O. Statistical methods to analyze adverse events data of randomized clinical trials. *Journal of Biopharmaceutical Statistics* 2009; 19: 889-899. DOI: http://dx.doi.org/10.1080/10543400903105463.
6. Tsang R, Colley L and Lynd LD. Inadequate statistical power to detect clinically significant differences in adverse event rates in randomized controlled trials. *Journal of Clinical Epidemiology* 2009; 62: 609-616.
7. Ioannidis JPA and Contopoulos-Ioannidis DG. Reporting of safety data from randomised trials. *The Lancet* 1998; 352: 1752-1753. DOI: 10.1016/S0140-6736(05)79825-1.
8. Edwards JE, McQuay HJ, Moore RA, et al. Reporting of Adverse Effects in Clinical Trials Should Be Improved. *Journal of Pain and Symptom Management* 1999; 18: 427-437. DOI: 10.1016/S0885-3924(99)00093-7.
9. Ioannidis JA and Lau J. Completeness of safety reporting in randomized trials: An evaluation of 7 medical areas. *JAMA* 2001; 285: 437-443. DOI: 10.1001/jama.285.4.437.
10. Pitrou I, Boutron I, Ahmad N, et al. Reporting of safety results in published reports of randomized controlled trials. *Archives of Internal Medicine* 2009; 169: 1756-1761. DOI: 10.1001/archinternmed.2009.306.
11. Cornelius VR, Sauzet O, Williams JE, et al. Adverse event reporting in randomised controlled trials of neuropathic pain: Considerations for future practice. *PAIN* 2013; 154: 213-220. DOI: 10.1016/j.pain.2012.08.012.
12. Maggi CB, Griebeler IH and Dal Pizzol Tda S. Information on adverse events in randomised clinical trials assessing drug interventions published in four medical journals with high impact factors. *Int J Risk Saf Med* 2014; 26: 9-22. 2014/05/07. DOI: 10.3233/JRS-140609.
13. Smith SM, Wang AT, Katz NP, et al. Adverse event assessment, analysis, and reporting in recent published analgesic clinical trials: ACTTION systematic review and recommendations. *PAIN* 2013; 154: 997-1008. DOI: 10.1016/j.pain.2013.03.003.
14. Peron J, Maillet D, Gan HK, et al. Adherence to CONSORT adverse event reporting guidelines in randomized clinical trials evaluating systemic cancer therapy: a systematic review. *J Clin Oncol* 2013; 31: 3957-3963. 2013/09/26. DOI: 10.1200/JCO.2013.49.3981.
15. Favier R and Crépin S. The reporting of harms in publications on randomized controlled trials funded by the “Programme Hospitalier de Recherche Clinique,” a French academic funding scheme. *Clinical Trials* 2018; 0: 1740774518760565. DOI: 10.1177/1740774518760565.
16. Hum SW, Golder S and Shaikh N. Inadequate harms reporting in randomized control trials of antibiotics for pediatric acute otitis media: a systematic review. *Drug Safety* 2018 May 08. DOI: 10.1007/s40264-018-0680-0.

For peer review only

17. Ioannidis JA, Evans SW, Gøtzsche PC, et al. Better reporting of harms in randomized trials: An extension of the consort statement. *Annals of Internal Medicine* 2004; 141: 781-788. DOI: 10.7326/0003-4819-141-10-200411160-00009.
18. Lineberry N, Berlin JA, Mansi B, et al. Recommendations to improve adverse event reporting in clinical trial publications: A joint pharmaceutical industry/journal editor perspective. *BMJ (Online)* 2016; 355: i5078.
19. StataCorp. Stata Statistical Software: Release 15. College Station, TX: StataCorp LLC, 2017.
20. Litonjua AA, Carey VJ, Laranjo N, et al. Effect of Prenatal Supplementation With Vitamin D on Asthma or Recurrent Wheezing in Offspring by Age 3 Years: The VDAART Randomized Clinical Trial. *JAMA* 2016; 315: 362-370. 2016/01/28. DOI: 10.1001/jama.2015.18589.
21. Miller PD, Hattersley G, Riis BJ, et al. Effect of Abaloparatide vs Placebo on New Vertebral Fractures in Postmenopausal Women With Osteoporosis: A Randomized Clinical Trial. *JAMA* 2016; 316: 722-733. 2016/08/18. DOI: 10.1001/jama.2016.11136.
22. Libman IM, Miller KM, DiMeglio LA, et al. Effect of Metformin Added to Insulin on Glycemic Control Among Overweight/Obese Adolescents With Type 1 Diabetes: A Randomized Clinical Trial. *JAMA* 2015; 314: 2241-2250. 2015/12/02. DOI: 10.1001/jama.2015.16174.
23. Writing Committee for the Diabetic Retinopathy Clinical Research Network, Gross JG, Glassman AR, et al. Panretinal Photocoagulation vs Intravitreous Ranibizumab for Proliferative Diabetic Retinopathy: A Randomized Clinical Trial. *JAMA* 2015; 314: 2137-2146. 2015/11/14. DOI: 10.1001/jama.2015.15217.
24. Myles PS, Smith JA, Forbes A, et al. Stopping vs. Continuing Aspirin before Coronary Artery Surgery. *N Engl J Med* 2016; 374: 728-737. 2016/03/05. DOI: 10.1056/NEJMoa1507688.
25. Billings FTt, Hendricks PA, Schildcrout JS, et al. High-Dose Perioperative Atorvastatin and Acute Kidney Injury Following Cardiac Surgery: A Randomized Clinical Trial. *JAMA* 2016; 315: 877-888. 2016/02/26. DOI: 10.1001/jama.2016.0548.
26. Beardsley J, Wolbers M, Kibengo FM, et al. Adjunctive Dexamethasone in HIV-Associated Cryptococcal Meningitis. *N Engl J Med* 2016; 374: 542-554. 2016/02/11. DOI: 10.1056/NEJMoa1509024.
27. Kor DJ, Carter RE, Park PK, et al. Effect of Aspirin on Development of ARDS in At-Risk Patients Presenting to the Emergency Department: The LIPS-A Randomized Clinical Trial. *JAMA* 2016; 315: 2406-2414. 2016/05/18. DOI: 10.1001/jama.2016.6330.
28. Nichol A, French C, Little L, et al. Erythropoietin in traumatic brain injury (EPO-TBI): a double-blind randomised controlled trial. *The Lancet* 2015; 386: 2499-2506. DOI: 10.1016/s0140-6736(15)00386-4.
29. Aitken E, Jackson A, Kearns R, et al. Effect of regional versus local anaesthesia on outcome after arteriovenous fistula creation: a randomised controlled trial. *The Lancet* 2016; 388: 1067-1074. DOI: 10.1016/s0140-6736(16)30948-5.
30. Kaul U, Bangalore S, Seth A, et al. Paclitaxel-Eluting versus Everolimus-Eluting Coronary Stents in Diabetes. *N Engl J Med* 2015; 373: 1709-1719. 2015/10/16. DOI: 10.1056/NEJMoa1510188.
31. Cohen MS, Chen YQ, McCauley M, et al. Antiretroviral Therapy for the Prevention of HIV-1 Transmission. *N Engl J Med* 2016; 375: 830-839. 2016/07/19. DOI: 10.1056/NEJMoa1600693.
32. Isanaka S, Langendorf C, Berthe F, et al. Routine Amoxicillin for Uncomplicated Severe Acute Malnutrition in Children. *N Engl J Med* 2016; 374: 444-453. 2016/02/04. DOI: 10.1056/NEJMoa1507024.
33. Natalucci G, Latal B, Koller B, et al. Effect of Early Prophylactic High-Dose Recombinant Human Erythropoietin in Very Preterm Infants on Neurodevelopmental Outcome at 2 Years: A Randomized Clinical Trial. *JAMA* 2016; 315: 2079-2085. 2016/05/18. DOI: 10.1001/jama.2016.5504.
34. Sheehan WJ, Mauger DT, Paul IM, et al. Acetaminophen versus Ibuprofen in Young Children with Mild Persistent Asthma. *N Engl J Med* 2016; 375: 619-630. 2016/08/18. DOI: 10.1056/NEJMoa1515990.
35. Lind M, Hirsch IB, Tuomilehto J, et al. Liraglutide in people treated for type 2 diabetes with multiple daily insulin injections: randomised clinical trial (MDI Liraglutide trial). *BMJ* 2015; 351: h5364. 2015/10/30. DOI: 10.1136/bmj.h5364.

For peer review only - http://bmjopen.bmj.com/site/about/guidelines.xhtml

36. Lincoff AM, Mehran R, Povsic TJ, et al. Effect of the REG1 anticoagulation system versus bivalirudin on outcomes after percutaneous coronary intervention (REGULATE-PCI): a randomised clinical trial. *The Lancet* 2016; 387: 349-356. DOI: 10.1016/s0140-6736(15)00515-2.
37. Ribrag V, Koscielny S, Bosq J, et al. Rituximab and dose-dense chemotherapy for adults with Burkitt's lymphoma: a randomised, controlled, open-label, phase 3 trial. *The Lancet* 2016; 387: 2402-2411. DOI: 10.1016/s0140-6736(15)01317-3.
38. Nguyen QD, Merrill PT, Jaffe GJ, et al. Adalimumab for prevention of uveitic flare in patients with inactive non-infectious uveitis controlled by corticosteroids (VISUAL II): a multicentre, double-masked, randomised, placebo-controlled phase 3 trial. *The Lancet* 2016; 388: 1183-1192. DOI: 10.1016/s0140-6736(16)31339-3.
39. Rodes-Cabau J, Horlick E, Ibrahim R, et al. Effect of Clopidogrel and Aspirin vs Aspirin Alone on Migraine Headaches After Transcatheter Atrial Septal Defect Closure: The CANOA Randomized Clinical Trial. *JAMA* 2015; 314: 2147-2154. 2015/11/10. DOI: 10.1001/jama.2015.13919.
40. Ruperto N, Pistorio A, Oliveira S, et al. Prednisone versus prednisone plus ciclosporin versus prednisone plus methotrexate in new-onset juvenile dermatomyositis: a randomised trial. *The Lancet* 2016; 387: 671-678. DOI: 10.1016/s0140-6736(15)01021-1.
41. Whitehead KJ, Sautter NB, McWilliams JP, et al. Effect of Topical Intranasal Therapy on Epistaxis Frequency in Patients With Hereditary Hemorrhagic Telangiectasia: A Randomized Clinical Trial. *JAMA* 2016; 316: 943-951. 2016/09/07. DOI: 10.1001/jama.2016.11724.
42. Schulz KF, Altman DG, Moher D, et al. Consort 2010 statement: Updated guidelines for reporting parallel group randomized trials. *Annals of Internal Medicine* 2010; 152: 726-732. DOI: 10.7326/0003-4819-152-11-201006010-00232.
43. Lingvay I, Perez Manghi F, Garcia-Hernandez P, et al. Effect of Insulin Glargine Up-titration vs Insulin Degludec/Liraglutide on Glycated Hemoglobin Levels in Patients With Uncontrolled Type 2 Diabetes: The DUAL V Randomized Clinical Trial. *JAMA* 2016; 315: 898-907. 2016/03/05. DOI: 10.1001/jama.2016.1252.
44. Cornelius VR, Liu K, Peacock J, et al. Variation in adverse drug reactions listed in product information for antidepressants and anticonvulsants, between the USA and Europe: a comparison review of paired regulatory documents. *BMJ Open* 2016; 6. DOI: 10.1136/bmjopen-2015-010599.
45. Stephens MD, Talbot JC and Routledge PA. *The Detection of New Adverse Reactions*. 4 ed. London: Macmillan Reference 1998.
46. Bent S, Padula A and Avins AL. Brief communication: Better ways to question patients about adverse medical events: a randomized, controlled trial. *Annals of Internal Medicine* 2006; 144: 257-261. DOI: 10.7326/0003-4819-144-4-200602210-00007.
47. Davis S, Sun H and Jung K. Best practices for reporting safety data to data monitoring committees. *Trials Conference: 4th International Clinical Trials Methodology Conference , ICTMC and the 38th Annual Meeting of the Society for Clinical Trials United Kingdom* 2017; 18.
48. Furey A and Bechhofer R. Effective graphical analyses of adverse events in DMC reports. *Trials Conference: 4th International Clinical Trials Methodology Conference , ICTMC and the 38th Annual Meeting of the Society for Clinical Trials United Kingdom* 2017; 18.
49. Buhr KA, Downs M, Rhorer J, et al. Reports to Independent Data Monitoring Committees: An Appeal for Clarity, Completeness, and Comprehensibility. *Therapeutic Innovation & Regulatory Science* 2017: 2168479017739268. DOI: 10.1177/2168479017739268.
50. Amit O, Heiberger RM and Lane PW. Graphical approaches to the analysis of safety data from clinical trials. *Pharmaceutical Statistics* 2008; 7: 20-35.
51. Zink RC, Wolfinger RD and Mann G. Summarizing the incidence of adverse events using volcano plots and time intervals. *Clinical Trials* 2013; 10: 398-406.

For peer review only

52. Detry MA and Lewis RJ. The intention-to-treat principle: How to assess the true effect of choosing a medical treatment. *JAMA* 2014; 312: 85-86. DOI: 10.1001/jama.2014.7523.
53. Sample Closed Session DMC Report, https://www.biostat.wisc.edu/sites/default/files/Sample_Report_Closed_20160920.pdf (2016, accessed 26/03/2018).
54. Altman DG and Bland JM. Statistics notes: Absence of evidence is not evidence of absence. *BMJ* 1995; 311: 485. DOI: 10.1136/bmj.311.7003.485.
55. Jonville-Béra A, Giraudeau B and Autret-Leca E. Reporting of drug tolerance in randomized clinical trials: When data conflict with authors' conclusions. *Annals of Internal Medicine* 2006; 144: 306-307. DOI: 10.7326/0003-4819-144-4-200602210-00024.
56. Coovadia A, Abrams EJ, Strehlau R, et al. Efavirenz-Based Antiretroviral Therapy Among Nevirapine-Exposed HIV-Infected Children in South Africa: A Randomized Clinical Trial. *JAMA* 2015; 314: 1808-1817. 2015/11/04. DOI: 10.1001/jama.2015.13631.

For peer review only

Supplementary material

Table A1: Data items extracted from publications

			Items collected	Instructions
**Study details**		1	Study number	
		2	Journal	
		3	Funding source: public, private, both or unspecified.	Studies will be assumed to be funded by industry only if this is explicitly stated.
**Study characteristics**		4	Control: placebo, active or both	Select placebo if no active treatment is given, else active. Both should be selected for trials with multiple arms where there is at least one group receiving no active treatment and one group receiving an active treatment.
		5	Number of centres	
		6	Number randomised	
		7	Study duration (length of trial follow-up)	
**Methods**	Details of how AE outcomes were defined (coding, attribution) and were collected (mode of collection, timing)	8	Describe the collection method: passive surveillance, patient prompted, clinical examinations (e.g. vital signs or urine samples), and laboratory tests. (Select all that apply)	*Passive*: If authors state that AEs were collected throughout the study with no further information we will assume that collection was passive. *Prompted*: Prompted methods include, but are not limited to: questions about both specific events and AEs in general, questionnaires, or diaries.
		9	Stated the timing of collection.	
		10	Mention dictionary for coding of events: Researcher defined, MedDRA, CTCAE, WHO-ART, COSTART, ICD-10, other or not applicable	
		11	Describe who undertook the assessment of attribution to study drug: blinded assessor, unblinded assessor or not specified.	
**Planned analysis**	Details of any plans for analysing AE outcomes	12	Describe analysis for AE outcomes in the statistical methods.	Reference must be made to harmful events e.g. AEs or a specific harm event, this cannot be simply how binary events will be analysed.
		13	Define a 'safety' population for analysis.	
		14	Specify a planned interim analysis with stopping criteria: based on efficacy, based on safety, based on both efficacy and safety, yes but no other details given, no planned interim analysis or unclear	Criteria for stopping must be set out, it is not enough to say that the DMC reviewed the data.

For peer review only - http://bmjopen.bmj.com/site/about/guidelines.xhtml

**Results**	Details of what was reported and where	15	What was reported in the main paper: summaries of type of AEs (e.g. AE, SAE, AR, ADR), actual AE terms, both, neither or not applicable?	Not applicable is relevant when for example authors explicitly state that there are no events or there is only one event so summaries are inappropriate.
		16	What was reported in the appendix: summaries of type of AEs (e.g. AE, SAE, AR, ADR), actual AE terms, both, neither or not applicable?	Not applicable is relevant when for example authors explicitly state that there are no events or there is only one event so summaries are inappropriate.<br>We will only search the appendix/supplementary material for AE data if the main article makes reference to it.
		17	Who was the AE analysis performed on: all randomised, participants who took at least a single dose, other or not specified?	
		18	How were number of drop-outs/withdrawals reported: By treatment arm, overall, not reported or not applicable?	Not applicable is relevant when there are no drop-outs/withdrawals.<br>This does not include discontinuation of treatment.
		19	Were drop-outs/withdrawals due to AEs reported: Yes, no or not applicable?	Not applicable if drop-outs/withdrawals are not reported or if it is reported that there are no drop-outs/withdrawals.
		20	Were specific AEs causing withdrawals reported: Yes, no or not applicable?	Not applicable if drop-outs/withdrawals due to AEs are not reported or if it is reported that there are no drop-outs/withdrawals due to AEs.
		21	What was the selection criteria for the AEs reported?	Free text response where possibilities can include for example**:** most frequent, above a severity threshold, SAEs.<br>Include details of what's in the main journal article and what is in the appendix separately.
	Details of how AEs were summarised and presented - binary outcomes	22	What summary information was given: Number of people, number of events, both, unclear, not summarised or not applicable?	Only select 'number of events' if presented for each individual event not just overall number of events.<br>Not applicable is only relevant when report that there are no AEs.
		23	What analysis was performed: frequencies, percentages, differences and 95% confidence intervals, significance tests, other? (Select all that apply)	

For peer review only

	Details of how AEs were summarised and presented - continuous outcomes	24	Were continuous outcomes dichotomised: Yes for all, yes for some, no or not applicable?	This includes measures that will have been captured as continuous and then dichotomised for example blood levels, blood pressure etc.
		25	If continuous outcomes were analysed as continuous what analysis was performed: differences in measures of central tendency, significance tests, other? (Select all that apply)	
	Details of how AEs were summarised and presented	26	Were signal detection methods used?	
		27	Were any graphical summaries of AEs presented?	
		28	Were severity ratings given: Yes for all, yes for some, no or not applicable?	
		29	Were numbers of serious events presented: Yes by treatment arm, yes overall, no or not applicable?	If death is reported as part of the efficacy outcome it is not enough to constitute reporting serious events.
		30	Were serious events coded as treatment related: Yes for all, yes for some, no or not applicable?	
		31	Provided information on the duration of events?	This refers to the length of the actual AE i.e. how long did it last.
		32	Provided information on the timing of events?	This refers to the time of onset of the AE.
		33	Accounted for multiplicity of statistical tests?	
		34	Referenced CONSORT extension for harms?	

For peer review only - http://bmjopen.bmj.com/site/about/guidelines.xhtml

Table A2: Stopping criteria for safety

Study	Main article text	Appendix text
Myles et al.[24]	"O'Brien–Fleming stopping boundaries were used to assess efficacy, and *a less stringent boundary was used to assess harm*."	
Billings et al.[25]	"The data and safety monitoring board (DSMB) reviewed patient recruitment practices, safety reporting, and data quality after 30 patients completed the study; performed an interim analysis after 277 patients … had completed the study to assess *safety of the intervention*; and performed a second interim analysis after 546 patients … had completed the study to assess the safety, efficacy, and futility of the intervention. The DSMB made recommendations based on qualitative assessments of the safety, efficacy, and futility of the intervention…"	"*Suspend enrolment in any study arm … due to safety concerns based on study intervention. Safety concerns include*: • Increase in in-hospital all-cause mortality in subjects randomized to A or B such that the DSMB deems the increase is excessive compared to A or B. • Increased treatment toxicity in either treatment group deemed excessive. Toxicity is defined as moderate or severe myalgias. • Increased severity of adverse events deemed "Probably Related" or "Possibly Related" to study intervention in either treatment group. Itemized adverse event reports separated by treatment will be provided. • Increased AKI incidence in either treatment group deemed excessive. • Increased incidence of stroke or hemodialysis requirement in either group (secondary endpoints) deemed excessive."
Beardsley et al.[26]	"An independent data and safety monitoring committee oversaw trial safety and analyzed unblinded data after every 50 deaths, according to its charter ..."	"The Haybittle-Peto boundary, requiring p<0.001 at interim analysis to consider stopping for efficacy, will be used as guidance. *A level of significance of 1% will be used as a guide for stopping the trial early because of a detected harm of dexamethasone*. In addition, the DMEC will receive conditional power curves to assess whether it remains realistic that the trial will demonstrate superiority of dexamethasone conditional on the data accrued up to the point of the interim analysis. Importantly, the DMEC recommendations will not be based purely on statistical tables but will also use clinical judgment."
Kor et al.[27]	"In addition to statistical criteria for significance, the study included a priori "go-no-go" definitions for recommending continuation to phase 3 study … Briefly, continuation to phase 3 would occur with a positive primary outcome finding along with an acceptable safety profile. An acceptable safety profile was defined as a serious adverse event profile for aspirin that was not statistically worse than placebo (95% CI for the relative risk of any serious adverse event covers the null value of relative risk = 1.0). The "no-go decision" was defined as early termination by the data and safety monitoring board for safety or unfavorable risk/benefit ratio. An indeterminate case in which there was a non–statistically significant effect but this effect was in a clinically meaningful direction was also defined."	Initiate Phase III Study: Demonstrated efficacy signal in addition to adequate safety profile Criteria: Early termination for benefit at interim analysis or p<0.08885 at final analysis (alpha=0.10 for study). *Serious adverse event profile of ASA not statistically worse than placebo (95% confidence interval for the relative risk of any SAE covers the null value of RR=1.0).* Further Development Potentially Required: Weak efficacy signal Criteria: Primary endpoint did not achieve a priori level of significance but there were at least a general consistency of secondary endpoints indicating propensity for efficacy with a larger sample size and/or more specific primary endpoint. Abandon Treatment Platform: Harm (in efficacy or safety endpoints) Criteria: Study terminated early per recommendation *by DSMB for safety and/or risk/benefit ratio concerns* (i.e., stop for futility, harm, unacceptable risk profile, etc.)

For peer review only - http://bmjopen.bmj.com/site/about/guidelines.xhtml

Nichol et al.[28]	We used a group sequential statistical approach to do two equally spaced pre-planned interim analyses (at 33% and 67% of total recruitment) to assess accumulated safety data (differential proportions of deep venous thrombosis and total mortality). This ***approach was chosen to provide for early stopping for probable harm*** or strong evidence of benefit. We applied the Haybittle-Peto criterion ($\|Zk\| \geq 3$) for early stopping at these analyses.	

For peer review only

For peer review only - http://bmjopen.bmj.com/site/about/guidelines.xhtml

Table A3: Selection criteria used to select AEs presented in the main journal report

Selection criteria	n	%
All AEs presented	20	10.87
AEs in greater than x% in any group	10	5.43
AEs in greater than x% in treatment group	4	2.17
AEs in greater than x% in all patients	1	0.54
Most common (no criteria specified)	9	4.89
Predefined AEs	26	14.13
SAEs	15	8.15
AEs leading to study drug discontinuation/interruption	3	1.63
Treatment related AEs	5	2.72
Grade 3>= events	9	4.89
AEs in greater than x% in any group & predefined/special interest AEs	4	2.17
AEs in greater than x% in any group & frequency between groups differed by more than y% & predefined/special interest AEs	1	0.54
AEs in greater than x% in all patients & predefined/special interest AEs	3	1.63
AEs in greater than x% in treatment group & AEs of special interest	2	1.09
AEs in greater than x% in any group & all SAEs	2	1.09
AEs in greater than x% in all patients & all SAEs	1	0.54
AEs in greater than x% in any group & SAEs related to treatment	1	0.54
Most common (no criteria specified) & predefined/special interest AEs	3	1.63
Most common (no criteria specified) & all SAEs	4	2.17
Most common (no criteria specified) & all SAEs & AEs leading to study drug discontinuation/interruption	1	0.54
Most common (no criteria specified) & treatment related SAEs	1	0.54
AEs where frequency between groups differed by more than y% & all SAEs	1	0.54
AEs of special interest	6	3.26
Grade >=3 AEs in greater than x% of patients	1	0.54
Grade >=3 AEs in greater than x% in intervention & y% in control	1	0.54
Most common (no criteria specified) grade 3>= AEs	1	0.54
Most common SAEs (no criteria specified)	1	0.54
SAEs & AE of special interest	1	0.54
Treatment related AEs in greater than x% of patients	1	0.54
Treatment related AEs in greater than x% in any group	1	0.54
AEs in greater than x% in treatment group & SAEs	1	0.54
AEs in greater than x% in treatment group & SAEs & predefined AEs	2	1.09
AEs in greater than x% in any group & significantly different & SAEs	1	0.54
AEs in greater than x% in any group & treatment related AEs/SAEs	2	1.09
AEs in greater than x% in treatment group & treatment related AEs & SAEs	1	0.54
AEs in greater than x% in treatment group & treatment related AEs in greater than y% in all patients	1	0.54
AEs in greater than x% in any group & Grade 3>= events	1	0.54
AEs in greater than x% in all patients & Grade 3>= events	1	0.54
AEs in greater than x% in all patients & Grade 2>= treatment related AEs	1	0.54
AEs in greater than x% in any group & Grade 3>= events in greater than y% in any group	1	0.54
AEs in greater than x% in any group & SAEs in treatment group	1	0.54
AEs in greater than x% in any group & AEs of special interest & most common (no criteria specified) AEs leading to treatment discontinuation/interruption & predefined AEs	1	0.54
AEs in greater than x% in any group, AEs of special interest in greater than y% in treatment group & treatment related deaths	1	0.54

For peer review only

AEs in greater than x% in treatment group & SAEs in greater than y% in any group	1	0.54
AEs and SAEs occurring more often in treatment group than control	1	0.54
AEs in greater than x% in treatment group & occurred more often in treatment group than control & predefined/special interest AEs	1	0.54
AEs in greater than x% in any group & frequency between groups differed by more than y%, SAEs in greater than z% in any group & all grade >=3 AEs	1	0.54
AEs in greater than x% patients & more than y% difference between treatment groups & AEs leading to treatment discontinuation/interruption & most common SAEs (no criteria specified) & death	1	0.54
Predefined AEs, AEs leading to hospitalisation/death/study drug discontinuation/interruption & SUSARS	2	1.09
Some form of overall summary	6	3.26
Not specified how selected	6	3.26
Not summarised in main paper	11	5.98

For peer review only

Table A4: Selection criteria used to select AEs presented in the appendix

Selection criteria	n	%
All AEs	18	9.78
SAEs	18	9.78
All AEs & SAEs	4	2.17
AEs in greater than x% in any group	7	3.8
AEs in greater than x% in treatment group	2	1.09
AEs in greater than x% in all patients	1	0.54
AEs in greater than x% in any group & all SAEs	2	1.09
AEs in greater than x% in treatment group & all SAEs	1	0.54
AEs in greater than x% in all patients & all SAEs	3	1.63
AEs in greater than x% in treatment group & all SAEs	1	0.54
AEs in greater than x% in treatment group & greater than in control group & all SAEs	1	0.54
SAEs in greater than x% in any group	1	0.54
AEs in greater than x% in any group & SAEs in greater than y% in any group	1	0.54
AEs in greater than x% in any group & AEs of special interest	2	1.09
Treatment related AEs	5	2.72
Treatment related AEs in greater than x% in any group	2	1.09
Grade 3>= events	2	1.09
Predefined AEs	8	4.35
AEs of special interest	1	0.54
AEs leading to study drug discontinuation/interruption	2	1.09
AEs leading to study drug discontinuation & SAEs	1	0.54
Grade 3>= events leading to study drug discontinuation & grade 3>= laboratory results	1	0.54
Treatment related AEs & AEs leading to study drug discontinuation	1	0.54
AEs in greater than x% in all patients leading to treatment discontinuations, SAEs in greater than x% in any group, serious predefined/special interest AEs and clinically significant laboratory results	1	0.54
AEs in greater than x% in any group, treatment related AEs in greater than x% in any group, treatment related SAEs and select AEs	1	0.54
Clinical laboratory data	1	0.54
Predefined AEs, AEs leading to hospitalisation/death/study drug discontinuation/interruption & SUSARS	3	1.63
Deaths	2	1.09
Some form of overall summary	5	2.72
Not specified how selected	2	1.09
Not summarised in the appendix	84	45.65

For peer review only

For peer review only - http://bmjopen.bmj.com/site/about/guidelines.xhtml

Table A5: Population used for AE analysis

Analysis population	n	%
Those that took at least a single dose	75	40.76
All randomised	54	29.35
Randomised and not withdrawn/ineligible	19	10.33
Not specified	17	9.24
Not applicable	3	1.63
Took a single dose and underwent AE/toxicity assessment	3	1.63
Active treatment groups	2	1.09
Completed treatment and assessed for primary outcome	2	1.09
Other	2	1.09
Patients who treatment was at least attempted on	1	0.54
Intention-to-treat population	1	0.54
Randomised and assessed for primary outcome	1	0.54
Randomised and attended at least on follow-up visit	1	0.54
Randomised and remained in follow-up	1	0.54
Randomised and underwent AE/toxicity assessment	1	0.54
Randomised, eligible and received at least a single dose	1	0.54

For peer review only

For peer review only - http://bmjopen.bmj.com/site/about/guidelines.xhtml

Reporting checklist for systematic review and meta-analysis.

Based on the PRISMA guidelines.

Instructions to authors

Complete this checklist by entering the page numbers from your manuscript where readers will find each of the items listed below.

Your article may not currently address all the items on the checklist. Please modify your text to include the missing information. If you are certain that an item does not apply, please write "n/a" and provide a short explanation.

Upload your completed checklist as an extra file when you submit to a journal.

In your methods section, say that you used the PRISMA reporting guidelines, and cite them as:

Moher D, Liberati A, Tetzlaff J, Altman DG, The PRISMA Group. Preferred Reporting Items for Systematic Reviews and Meta-Analyses: The PRISMA Statement

		Reporting Item	Page Number
	#1	Identify the report as a systematic review, meta-analysis, or both.	1
Structured summary	#2	Provide a structured summary including, as applicable: background; objectives; data sources; study eligibility criteria, participants, and interventions; study appraisal and synthesis methods; results; limitations; conclusions and implications of key findings; systematic review registration number	2
Rationale	#3	Describe the rationale for the review in the context of what is already known.	5-6
Objectives	#4	Provide an explicit statement of questions being addressed with reference to participants, interventions, comparisons, outcomes, and study design (PICOS).	6

For peer review only

Protocol and registration	#5	Indicate if a review protocol exists, if and where it can be accessed (e.g., Web address) and, if available, provide registration information including the registration number.	n/a - review protocol was not published
rEEligibility criteria	#6	Specify study characteristics (e.g., PICOS, length of follow-up) and report characteristics (e.g., years considered, language, publication status) used as criteria for eligibility, giving rational	6-7
Information sources	#7	Describe all information sources in the search (e.g., databases with dates of coverage, contact with study authors to identify additional studies) and date last searched.	6
Search	#8	Present full electronic search strategy for at least one database, including any limits used, such that it could be repeated.	n/a - we manually searched the electronic contents table of the journals for reports of original RCTs
Study selection	#9	State the process for selecting studies (i.e., for screening, for determining eligibility, for inclusion in the systematic review, and, if applicable, for inclusion in the meta-analysis).	7
Data collection process	#10	Describe the method of data extraction from reports (e.g., piloted forms, independently by two reviewers) and any processes for obtaining and confirming data from investigators.	7
Data items	#11	List and define all variables for which data were sought (e.g., PICOS, funding sources), and any assumptions and simplifications made.	7

For peer review only

Risk of bias in individual studies	#12	Describe methods used for assessing risk of bias in individual studies (including specification of whether this was done at the study or outcome level, or both), and how this information is to be used in any data synthesis.	n/a – the review was to identify current practice, we did not look at and synthesize the actual results of individual studies and as such this assessment was not relevant
Summary measures	#13	State the principal summary measures (e.g., risk ratio, difference in means).	8
Planned methods of analyis	#14	Describe the methods of handling data and combining results of studies, if done, including measures of consistency (e.g., I2) for each meta-analysis.	8
Risk of bias across studies	#15	Specify any assessment of risk of bias that may affect the cumulative evidence (e.g., publication bias, selective reporting within studies).	n/a – please see item #12
Additional analyses	#16	Describe methods of additional analyses (e.g., sensitivity or subgroup analyses, meta-regression), if done, indicating which were pre-specified.	n/a – no such analysis was performed
Study selection	#17	Give numbers of studies screened, assessed for eligibility, and included in the review, with reasons for exclusions at each stage, ideally with a flow diagram.	n/a – manual search resulted in only eligible articles being downloaded
Study characteristics	#18	For each study, present characteristics for which data were extracted (e.g., study size, PICOS, follow-up period) and provide the citation.	9
Risk of bias within studies	#19	Present data on risk of bias of each study and, if available, any outcome-level assessment (see Item 12).	n/a – please see item #12
Results of individual studies	#20	For all outcomes considered (benefits and harms), present, for each study: (a) simple summary data for each intervention group	n/a – only simple descriptive statistics are presented to describe current practice

For peer review only

		and (b) effect estimates and confidence intervals, ideally with a forest plot.	
Synthesis of results	#21	Present the main results of the review. If meta-analyses are done, include for each, confidence intervals and measures of consistency.	9-13
Risk of bias across studies	#22	Present results of any assessment of risk of bias across studies (see Item 15).	n/a – please see item #12
Additional analysis	#23	Give results of additional analyses, if done (e.g., sensitivity or subgroup analyses, meta-regression [see Item 16]).	n/a – please see item #16
Summary of Evidence	#24	Summarize the main findings, including the strength of evidence for each main outcome; consider their relevance to key groups (e.g., health care providers, users, and policy makers	13-21
Limitations	#25	Discuss limitations at study and outcome level (e.g., risk of bias), and at review level (e.g., incomplete retrieval of identified research, reporting bias).	26/27
Conclusions	#26	Provide a general interpretation of the results in the context of other evidence, and implications for future research.	27
Funding	#27	Describe sources of funding or other support (e.g., supply of data) for the systematic review; role of funders for the systematic review.	28

The PRISMA checklist is distributed under the terms of the Creative Commons Attribution License CC-BY. This checklist was completed on 29. May 2018 using http://www.goodreports.org/, a tool made by the EQUATOR Network in collaboration with Penelope.ai

For peer review only

BMJ Open

Analysis and reporting of adverse events in randomised controlled trials: a review

Journal:	*BMJ Open*
Manuscript ID	bmjopen-2018-024537.R2
Article Type:	Research
Date Submitted by the Author:	21-Dec-2018
Complete List of Authors:	Phillips, Rachel; Imperial College London, Faculty of Medicine, School of Public Health<br>Hazell, Lorna; Drug Safety Research Unit, Clinical Research; University of Portsmouth, Associate Dept. of Pharmacy and Biomedical Sciences<br>Sauzet, Odile; Universität Bielefeld,<br>Cornelius, Victoria; Imperial College London Faculty of Medicine, School of Public Health
**Primary Subject Heading**:	Research methods
Secondary Subject Heading:	Medical publishing and peer review
Keywords:	Randomised controlled trials, Harm data, Adverse drug reactions, Systematic review, Investigational drug, Adverse events < THERAPEUTICS

SCHOLARONE™
Manuscripts

Analysis and reporting of adverse events in randomised controlled trials: a review

Authors:

Rachel Phillips* - Imperial College London, London, United Kingdom

Lorna Hazell – Drug Safety Research Unit, Southampton, United Kingdom

Odile Sauzet – Bielefeld University, Bielefeld, Germany

Victoria Cornelius - Imperial College London, London, United Kingdom

* Corresponding author

Address: Imperial Clinical Trials Unit, Imperial College London, 1st Floor Stadium House, 68 Wood Lane, London, W12 7RH

Email: r.phillips@imperial.ac.uk

Telephone: 020 759 49356

Word Count: 4319

For peer review only

For peer review only - http://bmjopen.bmj.com/site/about/guidelines.xhtml

Abstract

Objective

To ascertain current approaches to the collection, reporting and analysis of adverse events (AEs) in randomised controlled trials (RCTs) with a primary efficacy outcome.

Design

A review of clinical trials of drug interventions from four high impact medical journals.

Data sources

Electronic contents table of the BMJ, the Journal of the American Medical Association, the Lancet, and the New England Journal of Medicine were searched for reports of original RCTs published between September 2015 and September 2016.

Methods

A pre-piloted checklist was used and single data extraction was performed by three reviewers with independent check of a randomly sampled subset to verify quality. We extracted data on collection methods, assessment of severity and causality, reporting criteria, analysis methods and presentation of AE data.

Results

We identified 184 eligible reports (BMJ n=3; JAMA n=38, Lancet n=62; and NEJM n=81). Sixty-two percent reported some form of spontaneous AE collection but only 29% included details of specific prompts used to ascertain AE data. Numbers that withdrew from the trial were well reported (80%),

For peer review only - http://bmjopen.bmj.com/site/about/guidelines.xhtml

however only 35% of these reported whether withdrawals were due to AEs. Results presented and analysis performed was predominantly on 'patients with at least 1 event' with 84% of studies ignoring repeated events. Despite a lack of power to undertake formal hypothesis testing, 47% performed such tests for binary outcomes.

Conclusions

This review highlighted that the collection, reporting and analysis of AE data in clinical trials is inconsistent and RCTs as a source of safety data are underutilised. Areas to improve include reducing information loss when analysing at patient level and inappropriate practice of underpowered multiple hypothesis testing. Implementation of standard reporting practices could enable a more accurate synthesis of safety data and development of guidance for statistical methodology to assess causality of AEs could facilitate better statistical practice.

Keywords

Randomised controlled trials; adverse events; harm data; adverse drug reactions; review; investigational drug.

For peer review only

Article Summary

Strengths and Limitations of this study

1. This is the first review to examine and quantify AE analysis practice in RCTs published in high impact journals.
2. This review identifies weakness that need to be addressed as well as good practice that could be adopted.
3. Articles included in this review were published in four of the top ranked general medical journals therefore results are likely to be biased towards better practice and are only for year 2015-2016 and as such may not reflect the most current practice.

For peer review only

INTRODUCTION

The methods to analyse and report outcomes to measure benefit from randomised controlled trials (RCTs) are well developed but this progress has not been matched for adverse event (AE) outcomes. An adverse event is defined as 'any untoward medical occurrence that may present during treatment with a pharmaceutical product but which does not necessarily have a causal relationship with this treatment'.[1] An adverse drug reaction (ADR) is defined as 'a response to a drug which is noxious and unintended …' where a causal relationship is 'at least a reasonable possibility'.[1, 2] RCTs provide an opportunity to compare rates of AEs between arms allowing causality to be evaluated. However, current analysis and reporting practices are inadequate.

There are many challenges associated with analysing and reporting AEs in clinical trials. RCTs are typically designed to determine the efficacy of an intervention but are often underpowered to detect important differences in AEs between arms which may suggest an ADR. Often large numbers of AEs are reported during a study, sometimes exceeding the number of patients in the clinical trial. Performing hypothesis tests on these AEs would lead to issues of multiplicity, however any adjustment for multiplicity would make a 'finding untenable'.[3, 4] The use of hypothesis testing may result in the medicinal product being deemed unsafe and a trial being halted too early due to a chance imbalance, or conversely deemed safe and not stopped early enough resulting in more patients than necessary suffering an ADR.[3, 5, 6] Unlike efficacy outcomes which are well defined and restricted in number at the planning stage of a RCT, we collect numerous, undefined AEs in RCTs. Furthermore, AE collection requires additional information to be obtained on factors such as severity, timing and duration, number of occurrences and outcome, which for our efficacy outcomes would have all been predefined.

For peer review only

For peer review only - http://bmjopen.bmj.com/site/about/guidelines.xhtml

Previous studies have examined the methods for AE collection and presentation only, and highlighted the inadequacies in AE reporting in journal articles.[7-16] In 2004 the Consolidated Standards of Reporting Trials (CONSORT) Group produced an extension to their guidelines for reporting trial results to cover the reporting of harms, however implementation of these guidelines has been shown to be poor.[10, 14-17] Recently a joint pharmaceutical/journal collaboration published practical guidance and examples on what should be reported in journal articles and how it should be displayed to ensure transparency and aid clinical interpretation. They promote the use of clinical judgement in reporting rather than mandatory guidance.[18] Whilst this work has been undertaken there remains uncertainty about practice for reporting and presenting AE data, and in addition the analysis practice for AEs remains a neglected area for review.

The aim of this review was to evaluate current practice for collection, reporting and analysis of AEs in RCTs where the primary outcome was efficacy. The aim being to identify and promote any areas of good practice, whilst highlighting any areas for improvement.

METHODS

Search strategy

The top four general medical journals as ranked by impact factors that publish clinical trials of drug interventions were selected: The BMJ (Impact Factor 20.79), the Journal of the American Medical Association (JAMA, IF 44.41), the Lancet (IF 47.83), and the New England Journal of Medicine (NEJM, IF 72.41). Impact factors quoted are from 2016 to reflect the time period from which the articles were drawn. High impact journals were chosen as we would expect practice in these journals to be of high standard as they include statistical and methodological review. We limited the search to four

For peer review only - http://bmjopen.bmj.com/site/about/guidelines.xhtml

journals after an initial scoping review revealed around 100 studies would be eligible for inclusion, which was a feasible number to review given the time and resources available and would provide a sufficient number to evaluate practice. One reviewer manually searched the electronic contents table of the journals for reports of original RCTs published between September 2015 and September 2016, inclusive. Any queries regarding eligibility were reviewed and discussed with a second reviewer.

Selection criteria

The inclusion criteria were phase II-IV RCTs of drug interventions where the primary outcome was efficacy of the intervention. We did not restrict according to number of treatment arms and included both parallel and cluster RCTs. We excluded cross-over RCTs, RCTs with adaptive randomisation, observational studies, case reports, editorials and letters. We also excluded RCTs where the intervention was not a drug product (i.e. not classified as a clinical trial of an investigational medicinal product (CTIMP)). As the study aimed to assess how authors report and analyse AEs in studies where the primary outcome was efficacy, trials that were specifically designed to investigate safety as a primary outcome were not included.

Data extraction

Potentially eligible articles were identified based on titles and abstracts and the full text of these studies were retrieved. Supplementary material was also reviewed if readers were referred here from the main article for further results. Supplementary Table A1 lists all data items captured with guidance given to the reviewers for extraction. The items to be extracted were based on the work by

For peer review only - http://bmjopen.bmj.com/site/about/guidelines.xhtml

Cornelius et al. and the CONSORT harm extension with additional items added to capture more specific information on analysis practices.[11, 17] Specifically we focused on the following areas: how AE data was collected (mode of collection, timing) and defined (coding, attribution); how AEs were assessed in terms of severity of the event or relatedness to the medical intervention; if there was any planned AE analysis (final and interim monitoring plans and analysis populations); how events were selected for inclusion in the journal article; how summary event information was presented in the journal article and how AEs were analysed.[11] A more detailed rationale for the choice of items extracted is provided in the supplementary material (Table A2).

A data extraction sheet was piloted and then single data extraction was performed by three reviewers (RP, VC and LH) with 10% independent check of a randomly sampled subset to verify quality. Queries were also informally discussed between reviewers on an ongoing basis. Where specific items were flagged for poor agreement these were re-extracted. Any queries during data extraction were shared and disagreements between reviewers were resolved through discussion.

Data analysis

The proportion of trials reporting each item, 3-4 and 8-34 in supplementary Table A1 were calculated and summary statistics (median and ranges) were calculated for items 5-7. All analyses were performed in Stata version 15.[19] A risk of bias assessment was not undertaken as this study aimed to describe best practice and not evaluate outcomes.

Patient and public involvement

This review forms part of a wider research project that was developed with input from a range of patient representatives. There were no study participants directly involved in this review but the

For peer review only - http://bmjopen.bmj.com/site/about/guidelines.xhtml

original proposal and patient and public involvement (PPI) strategy were reviewed by service user representatives (with experience as clinical trial participants and PPI advisors) who provided advice specifically with regard to communication and dissemination to patient and public groups.

RESULTS

Data extraction

A total of 585 items were extracted twice across all three reviewers to check the quality of the data extraction. A total of 95 discrepancies were identified. This gave agreement of 84%. During this independent check several items were flagged for potential poor agreement. These items were 100% independently extracted by one author and verified. The items were: study duration; the AE collection method; timing of collection; how binary harm outcomes were summarised; whether continuous outcomes were dichotomised; if continuous outcomes were left as continuous how they were analysed.

Study characteristics

The search identified 184 eligible trial reports (BMJ n=3; JAMA n=38, Lancet n=62; and NEJM n=81) in which a total of 496911 participants were randomised with a median of 556 participants per trial (range 30, 205513; interquartile range (IQR) 281, 1704). The median trial follow-up was 52 weeks (range 48 hours to 10 years; IQR 24, 104 weeks) and 93% were multi-centre trials. Fifty-percent of

For peer review only - http://bmjopen.bmj.com/site/about/guidelines.xhtml

studies had an active comparator and over 50% of trials received some element of industry funding (Table 1).

Table 1: Characteristics of included studies

**Characteristic**	N=184		
	**Median**	**(IQR)**	**min, max**
**Sample size**	556	(281, 1704)	30, 205513
**Centres**[a]	35	(12, 100)	1, 1368
**Trial duration (weeks)**[b]	52	(24, 104)	0.3, 521
	**n**	**%**	
**Journal**			
**BMJ**	3	1.6	
**JAMA**	81	44.0	
**Lancet**	38	20.7	
**NEJM**	62	33.7	
**Funded by**			
**Public**	70	38.3	
**Industry**	80	43.7	
**Both**	33	18.0	
**Centre**			
**Single-centre**	12	7.0	
**Multi-centre**	161	93.0	
**Control**			
**Placebo**	95	51.6	
**Active**	80	43.5	
**Both**	8	4.4	
**Neither**[c]	1	0.5	

Abbreviations: *IQR = Inter-quartile range; min = minimum; and max = maximum*

[a]11 reports did not specify the number of centres

[b]2 reports did not specify trial duration

[c]One trial compared interventional drug to behavioural change intervention

Collection and assessment methods

For peer review only

For peer review only - http://bmjopen.bmj.com/site/about/guidelines.xhtml

Sixty-two percent (n=114) of reports made reference to some form of passive (e.g. spontaneously reported by patients) AE monitoring or collection methods. Of these only 46.5% (53/114) or 29% of total reports included specific details (prompts e.g. questions about specific events or AEs in general, questionnaires, or diaries) regarding these collection methods (Supplementary Table A3, examples 1-2).[20, 21] The timing of collection was well documented (91%, 48 out of 53 reports) in the reports that included specific details about the prompts used to collect AEs. Although specific details on clinical examinations (e.g. vital signs and blood pressure) and laboratory tests were not widely reported (only 57% of reports (95 out of 166 reports with clinical examinations and/or laboratory results presented) included details on the timing of such assessments) it was often clear from the results presented that participants had undergone these assessments (83% and 79% of studies reported clinical and laboratory results respectively) (Table 2).

For peer review only - http://bmjopen.bmj.com/site/about/guidelines.xhtml

Table 2: Collection, assessment and analysis methods reported by studies

Section	Component	Data item	N=184	
**Collection**			n	%
	**How was AE/harm information collected?**			
		Passive collection	114	62.0
		Prompted collection (n=114)	53	46.5
		No method of collection reported	70	38.0
	**Did they undertake proactive screening?**			
		Clinical examinations	153	83.2
		Laboratory tests	146	79.4
	**Timing of prompted collection specified** (n=53)		48	90.6
	**Timing of active collection specified** (n=166)		95	57.2
	**Which, if any, dictionary was used to code AE data?**			
		CTCAE	18	9.8
		MedDRA	43	23.4
		CTCAE and MedDRA	1	0.5
		DAIDS	2	1.1
		ICD-10	1	0.5
		Researcher defined	2	1.1
		Other	3	1.6
		No dictionary reported	114	62.0
**Assessment**				
	**Who assigned attribution to study drug?**			
		Blinded assessor	9	4.9
		Unblinded assessor	7	3.8
		Both	1	0.5
		Not specified	164	89.1
		Not applicable[a]	3	1.6
**Analysis**				
	**Was any analysis for AEs specified in the methods section?**			
		Yes	57	31.0
	**Was a population for AE analysis specified?**			
		Yes	82	44.6
	**Was there a planned interim analysis with stopping criteria?**			
		No	138	75.0
		Yes for efficacy	24	13.0
		Yes for efficacy & futility	11	6.0
		Yes for efficacy & safety	3	1.6
		Yes for efficacy, futility & safety	2	1.1
		Yes but no other details given	6	3.3

Abbreviations: *AE = Adverse event; CTCAE = Common Terminology Criteria for Adverse Events; MedDRA = Medical Dictionary for Regulatory Activities; DAIDS = The Division of AIDS; and ICD-10 = International Classification of Diseases 10th revision.*

NOTE: Denominator specified in item column if it differs from total sample

[a]3 reports made no reference to AE data throughout the article

For peer review only - http://bmjopen.bmj.com/site/about/guidelines.xhtml

Prespecified analysis

Thirty-one percent of reports provided information on the planned analysis for AEs in the statistical analysis section of the paper and 45% pre-specified a safety population (Supplementary Table A3, examples 3-4 and Table 2).[22, 23] A quarter of trials reported planned interim analysis with stopping criteria (Table 2), five (2.7%) of which included specific criteria on stopping for a harmful event (Supplement Table A4[24-28]).

Selection of AEs and reporting practices

Two reports only made generic statements regarding AE data: "*there were no significant adverse events related to the procedure*" and "*no excess in mortality or major adverse events were found…*". Three reports made no mention of AEs throughout the manuscript.[29-33]

Twenty-four (13%) trials only provided a summary of the number of AEs or serious AEs rather than listing the actual AEs that occurred. For example "*Six serious adverse events occurred in the acetaminophen group and 12 in the ibuprofen group.*"[34] Of these 24 trials, 10 did provide specific details of the types of events in an appendix. This means 8% of trials either did not report AEs or only included a summary (Table 3).

For peer review only - http://bmjopen.bmj.com/site/about/guidelines.xhtml

Table 3: Summaries of results presented by studies

Component	Data item	N=184	
		n	%
**What was reported in the manuscript?**			
	Actual AE terms	73	39.7
	Summaries of AE type (e.g. AE, SAE)	24	13.0
	Both	80	43.5
	Neither	7	3.8
**What was reported in the appendix?**			
	Actual AE terms	76	41.3
	Summaries of AE type (e.g. AE, SAE)	7	3.8
	Both	22	12.0
	Neither	3	1.6
	Not applicable[a]	76	41.3
**Which population was the AE analysis performed on?**			
	All randomised	54	29.4
	Those that took at least a single dose	75	40.8
	Other	35	19.0
	Not specified	17	9.2
	Not applicable[b]	3	1.6
**Were drop-outs/withdrawals reported?**			
	No	33	17.9
	Yes by treatment arm	144	78.3
	Yes overall	2	1.1
	Not applicable[c]	5	2.7
**Were withdrawals due to AEs reported?** (n=146)			
	No	89	61.0
	Yes	51	34.9
	Not applicable[d]	6	4.1
**Were specific AEs causing withdrawals reported?** (n=51)			
	No	39	76.5
	Yes	12	23.5
**How were binary AE outcomes summarised by arm?**			
	Not summarised[e]	6	3.3
	Number of people with an event	154	83.7
	Number of events	11	6.0
	Both	12	6.5
	Unclear	1	0.5
**Were frequencies of AEs reported by arm?**			
	No	5	2.7
	Yes for some	13	7.1
	Yes for all	160	87.0
	Not applicable[e]	6	3.3
**Were percentages of AEs reported by arm?**			
	No	18	9.8
	Yes for some	25	13.6

For peer review only - http://bmjopen.bmj.com/site/about/guidelines.xhtml

	Yes for all	135	73.4
	Not applicable[e]	6	3.3
**Were between arm differences and 95% CI of AEs reported?**			
	No	141	76.6
	Yes for some	18	9.8
	Yes for all	19	10.3
	Not applicable[e]	6	3.3
**Were statistical significance tests between arms on AEs reported?**			
	No	92	50.0
	Yes for some	31	16.9
	Yes for all	55	29.9
	Not applicable[e]	6	3.3
**Were continuous AEs outcomes dichotomised for summaries?**			
	No	10	5.4
	Yes for some	28	15.2
	Yes for all	108	58.7
	Not applicable	38	20.7
**If continuous outcomes were left as continuous what between arm analyses was performed?** (n=38)			
**Differences in measures of central tendency estimated with 95% CI**			
	No	23	60.5
	Yes for some	1	2.6
	Yes for all	14	36.8
**Between arm hypothesis tests performed**			
	No	12	31.6
	Yes for some	2	5.3
	Yes for all	24	63.2
**Were any 'signal detection' approaches used?**			
	No	184	100.0
	Yes	0	0.0
**Were there any graphical presentations of AE outcomes?**			
	No	162	88.0
	Yes	22	12.0
**Were summaries of severity rating of AEs reported?**			
	No	103	56.0
	Yes for some	41	22.3
	Yes for all	35	19.0
	Not applicable[f]	5	2.7
**Were number of serious AEs reported?**			
	No	44	23.9
	Yes overall	2	1.1
	Yes by treatment arm	132	71.7
	Not applicable[g]	6	3.3
**For serious AEs was relatedness given?** (n=134)			
	No	77	57.5
	Yes for some	18	13.4
	Yes for all	38	28.4

For peer review only - http://bmjopen.bmj.com/site/about/guidelines.xhtml

	Yes overall	1	0.8
**Were there any AEs where information on duration of events was reported?**			
	No	175	95.1
	Yes	9	4.9
**Were there any AEs where information on the time of occurrence of events was reported?**			
	No	132	71.7
	Yes	52	28.3
**If any significance tests were performed on AEs was multiplicity of events accounted for?**			
	No	81	44.0
	Yes	3	1.6
	Not applicable	100	54.4
**Did the report reference the CONSORT extension to harms**			
	No	184	100.0
	Yes	0	0.0

Abbreviations: *AE = Adverse Event; SAE = Serious Adverse Event; CI = Confidence Interval; and CONSORT = Consolidated Standards for Reporting Trials.*

[a] Make no reference to the appendix

[b] 3 reports made no reference to AE data throughout the article

[c] 5 reports indicate no withdrawals

[d] 6 reports specify the number of withdrawals and reasons but none of the reasons are related to AEs

[e] This includes 3 reports with no AE data (as per footnote [b]), 2 reports that provide generic statements regarding AE data and 1 report that only reported continuous outcomes

[f] This includes 3 reports with no AE data and 2 reports that provide generic statements regarding AE data (as per footnote [e])

[g] 6 papers specifically state that no serious adverse events occurred

Eighty-nine percent of trials reported a subset of all the AEs they collected. How AEs are 'selected' for inclusion in the article was not consistent or clear, and in 3% of studies it was impossible to discern how the authors had selected the AEs they presented for inclusion. Twenty-six percent of reports selected events based on a frequency threshold e.g. events experienced by greater than x% in any group; 9% of reports used a measure of severity to select events e.g. AEs of grade 3 or higher; 23% of reports included events based on seriousness; and 8% included AEs based on relatedness to treatment (percentages are not independent as the majority of reports used several different criteria for selection). Supplementary Tables A5 and A6 provide full details of selection criteria used.

For peer review only

For peer review only - http://bmjopen.bmj.com/site/about/guidelines.xhtml

We found that 41% of trials analysed AEs in participants that received at least one dose, 29% of trials used all randomised participants and 9% did not specify the analysis population (Table 3). Further details on analysis populations used are given in supplementary Table A7.

Nearly 80% of trials reported the number of participants who withdrew from the trial; of these 35% (51 of 146 reports) reported whether the withdrawals were due to AEs and of these 24% (12 of 51 reports) reported the actual events that caused withdrawals. Results presented and analysis performed was predominantly on 'patients with at least 1 event' with 84% of reports providing no information on the number of events occurring. An example of how to incorporate information on number of events is presented in reference [35]. Forty-one percent of trials reported information on the severity of AEs. Five percent of trials include a report of at least one event with duration, but presenting such data is limited in the main report. The trials that did present this information did so in a variety of ways. For example incorporating the information into the AE table with summary statistics such as the mean duration of certain events or presenting it for a subgroup of events in the footnotes of AE tables e.g. "*One event of non-serious squamous cell carcinoma (day 210, resolved on day 215; adalimumab treatment was not interrupted).*"[36-38] Twenty-eight percent of reports included information on the timing of AEs (Table 3).

Serious adverse events were typically well documented (73%) and six reports (3%) explicitly stated that no serious events had occurred. However, for forty-four reports (24%) it was not possible to discern if no serious events had occurred or whether they were simply omitted from the report. Forty-two percent (57 of 134 reports) of reports included details on whether the events had been classified as related to the intervention (Table 3).

For peer review only - http://bmjopen.bmj.com/site/about/guidelines.xhtml

Analysis of AE outcomes

Binary

The majority of trials summarised binary outcomes using frequencies (94%) and percentages (87%). Despite a lack of power to undertake formal hypothesis testing, 47% reported p-values for binary outcomes. For example "*There were no between-group differences in the rate of patients with at least 1 adverse event (16.7% [14 patients] in the clopidogrel group vs 21.8% [19 patients] in the placebo group; difference, −5.2% [95% CI, −17% to 6.6%]; P = .44)*." However, with a total safety population of 171 such a test would have only had 13% power to detect such a difference and was therefore substantially underpowered. The conclusion that "*No significant increase in adverse events was observed*" makes no reference to the 95% confidence interval presented which indicates that the findings were in fact compatible with a 17% decrease in experiencing at least on AE as well as a near 7% increase.[39]

Continuous

There was a pervasive practise (59%) of categorising continuous clinical and laboratory outcomes. Of the trials that did not dichotomise continuous AE data nearly 70% performed some form of statistical significance testing (Table 3). Whilst continuous outcomes do not suffer to the same degree regarding lack of power, multiple testing is still a problem, however no multiplicity corrections for continuous outcomes were performed.

Of the trials that performed statistical significance testing on AE data, only three made an adjustment for multiplicity of tests (all three on dichotomised outcomes).[36, 40, 41] Two of which used a Bonferroni correction and adjusted for the number of pairwise comparisons between each of the

For peer review only - http://bmjopen.bmj.com/site/about/guidelines.xhtml

treatment groups for each individual event rather than the total number of significance tests performed. As such both analyses would have still been effected by issues of multiple testing.

Twelve percent of reports used graphs to illustrate AE data (Table 3). The CONSORT extension highlighted the value of graphs for summarising such data, especially for conveying information on time-to-event outcomes.[42] An example of such a plot is included in the supplement of reference [43] (eFigure2).

We assessed any reference to the CONSORT harm extension and found that none of the included studies mentioned it. Of the four journals included in the review, the Lancet was the only journal that made specific reference to the harm extension in their guidelines to authors.

DISCUSSION

The safety profile of a medicinal product is established through evidence collected from several sources including clinical trials, observational studies and spontaneous reports.[44] The advantage of clinical trial data is that these provide a controlled comparison of the rate of AEs allowing causality to be evaluated but have the disadvantage that the sample size is often not large enough to detect rare ADRs.

To ensure that a useful and comprehensive picture of the safety profile is provided to all relevant parties clear reporting of AEs from clinical trials is required. Current research has shown the quality of reporting is substandard.[7-16] The aim of this study was to review current practice across four

For peer review only - http://bmjopen.bmj.com/site/about/guidelines.xhtml

leading medical journals for AE collection, analysis and reporting practices, highlighting any areas for improvement and examples of good practice.

Collection and assessment methods

The CONSORT extension to harm was developed with the aim to improve reporting of safety data in RCTs.[42] None of the included studies referenced the CONSTORT harm extension and of the items in our review that are covered in CONSORT many were not well reported.[17] This suggests that the CONSORT extension is not being routinely adopted by authors to aid their reporting. Most journals now request that authors include a completed CONSORT checklist when they submit their article but we are not aware of any journal that request the CONSORT harm extension to also be submitted. Of the four journals in this review the Lancet is the only journal that makes specific reference to the harm extension in their guidelines to authors. The CONSORT statement contains a single item related to safety, item 19: 'all important harms or unintended effects in each group' should be reported.[42] This may explain why some items listed on the CONSORT extension for harm were reported by so few trials. The mandatory submission of CONSORT harms by journals may support better reporting.

We found that the method of AE collection was poorly reported. This has important implications for the type and frequency of AEs reported with "passive collection resulting in fewer recorded AEs".[45, 46] Where the method was given the timing of collection was typically also reported and we would recommend continuation of this practice. The frequency of AE collection has further important implications on the number of events reported. More frequent assessment and longer follow-up will result in more AEs reported.[17] It is important to consider these factors when making conclusions about the safety profile.

For peer review only - http://bmjopen.bmj.com/site/about/guidelines.xhtml

The method of attribution between drug and AE was another area where reporting practice was inadequate. However, the joint pharmaceutical/journal collaboration indicate that such attribution has 'limited value' given the 'inherent subjectivity in such attribution'.[18]

Prespecified analysis

We found that formal assessments of AEs regarding stopping for emerging ADRs utilising statistical rules was rare. Subjective assessments of overwhelming amounts of data could easily lead to potential signals of harm being missed. There could be benefits to incorporating more objective statistical methods alongside clinical review to assist the evaluation of AE information to help better identify drug harm relationships. Graphical displays have gone some way towards aiding interpretation.[47-51]

Selection of AEs and reporting practices

Due to space constraints in journal reports AE information is often included in the appendix. Whilst we encourage use of appendices and supplementary material for including additional detail on AEs, we caution authors against depositing all AE data into such documents without attempting to present a summary of the AE profile in the main article. It is important that the main report strikes a balance between efficacy and harm therefore allowing a risk-benefit assessment to be made solely from the article.

For peer review only

For peer review only - http://bmjopen.bmj.com/site/about/guidelines.xhtml

The failure to report any information on AEs restricts interpretation and prevents a risk-benefit assessment. We identified two reports that made generic summaries of the overall safety profile and it was clear in both that there had been harmful effects. However, the authors did not include any further information. Three reports contained no information leaving readers uninformed as to any additional information these studies may provide on the safety profile. Ambiguous reporting prevents building an accurate picture of the safety profile. As such profiles are developed on accumulating evidence, it is important that each study report to the same standard and information is not wasted.

We found that the selection criteria used by authors to decide what AEs to include in the report were arbitrary and inconsistent. This will have important implications when synthesising data across studies to construct safety profiles. Authors would benefit from guidance to facilitate consistency but currently research in this area is lacking. Lineberry et al. recommended clinically relevant events that should always be reported (deaths, SAEs and events leading to discontinuation of intervention) and criteria that should be considered when deciding what other AEs to report e.g. interest based on the disease(s) under investigation, comorbidities of the study population, intervention mechanism, trial duration.[18] Standard outcomes for a drug class would be one potential solution to avoid issues of inconsistency suggested by Cornelius et al.[11]

CONSORT recommend that AE analyses should be performed on the intention-to-treat (ITT) population to maintain the random assignment.[17] However it is clear from our review that this population label is not always appropriately and consistently applied. There is a tendency for studies to make modifications to the ITT population. Using the ITT or modified-ITT population is likely to underestimate the risk by inflating the denominator with participants who may have never received the study drug.[52] Such estimates are appropriate for health economic evaluations where estimates

For peer review only - http://bmjopen.bmj.com/site/about/guidelines.xhtml

of the cost-effectiveness will inform policy level decisions regarding how to treat the population. However, a more appropriate population for AE analysis to inform prescriber and patient decisions may be those that receive at least one dose. It is important that authors clearly define and specify a suitable safety analysis population and consider how this affects their conclusions.

Proxy outcomes can be used as a measure of the impact of AEs on patients. Examples include the number of withdrawals due to any reason, withdrawals due to AEs, the number of events an individual experiences, the severity of the AE and the duration. A high proportion of trials reported withdrawal for any reason and this is likely to be as a result of the CONSORT recommendations.[42] The other outcomes were not frequently reported and increasing this could facilitate interpretation.[17] This information would permit better evaluation of the impact of AEs and the tolerability of the intervention to inform patients' and clinicians' treatment decisions. Reporting numbers that experience at least one event only and not providing information on repeated events masks valuable information that may be important to the patient and the cost-effectiveness evaluation. For example, chronic, repeated headaches over an extended duration will have an important impact for patients compared to a single headache or headaches over a short duration but it is not possible to distinguish between these two scenarios when reported as 'at least one event'.[18] Severity of events was also an important aspect that was often not differentiated. For example, there would be a different impact on patients' quality-of-life with mild compared to severe nausea, which could lead to changes in dosing regimens. Displaying such information for all AEs in tables would soon become overwhelming and make interpretation difficult. Graphical approaches have been suggested as a solution to aid review. Examples of such a plots can be found in reference [53]. Online appendices and supplementary material provide more opportunity to include this important information.

For peer review only - http://bmjopen.bmj.com/site/about/guidelines.xhtml

For serious adverse events information on the time of likely onset can be useful information to inform patient monitoring plans. For example, the documented risk of suicide and suicidal ideation within the first few weeks of starting anti-depressant allows patients and prescribers to remain alert and monitor closely for this period. Nearly a third of reports included such information and we would encourage authors to adopt this practice.

Analysis of AE outcomes

The majority of trials in this review included a balanced report of AEs alongside benefit. However many included generic statements regarding the safety profile such as 'the intervention was well tolerated' or 'the intervention exhibited a good safety profile' and these were frequently based on post-hoc statistical tests. Guidelines caution against such tests.[18] The results of which are difficult to interpret as a lack of significance does not indicate that the intervention is safe and conversely multiple testing without adjustment will increase the number of significant differences due to chance.[54, 55]

Graphs are an efficient method to convey and interpret large amounts of data and can make it easier to flag potential safety signals.[50, 51, 53] Twelve percent of studies included in the review used graphs to present AE data and an example of one such report is given in the supplementary eTable of reference [56].

Recommendations for consideration for immediate adoption by the clinical trial community are summarised in Table 4.

For peer review only - http://bmjopen.bmj.com/site/about/guidelines.xhtml

Table 4: Recommendations to improve adverse event analysis and reporting in clinical trial report publications

	Recommendation
**Analysis**	Incorporate objective statistical methods to assist the evaluation of adverse event information.
	Consider avoiding dichotomising continuous data.
	When count outcomes are available (such as repeated events within participants) use appropriate statistical methods.
	Clearly define exposure and specify a suitable safety analysis population.
	Use graphical approaches to help summarise large amounts of data.
**Reporting**	Report adverse event data according to the CONSORT harm checklist.
	Increase the uptake of mandatory submission of CONSORT harm by journals.
	Include a relevant summary of the adverse event profile in the main article. Resist depositing all adverse event data into appendices without summarising.

For peer review only

Limitations of trials

Trials are a valuable source for high quality adverse event data but compared to observational studies have smaller sample size, follow-up periods and generalisability, which restrict the ability to detect rare ADRs, ADRs with long latency and drug interactions in complex populations. The typical duration of a trial means there is often insufficient follow-up to fully characterise the safety profile as it provides limited information on long-term exposure. Stringent inclusion criteria restrict the population the intervention is assessed in and so limited information on drug-interactions is obtained.[5]

Limitations of this study

For peer review only - http://bmjopen.bmj.com/site/about/guidelines.xhtml

Articles included in this review were published in four of the top ranked medical journals therefore results are likely to be biased towards better findings than we would expect if we included all RCTs. Articles are only for year 2015-2016 and as such may not reflect the most current practice. We also acknowledge that only completing 10% independent check of extracted data would not have removed subjectivity from the data extraction but are happy that ongoing discussion between authors to clarify any queries would have kept this to a minimum. Despite these limitations, this review characterises what those leading the field are doing and provides some examples of good practice that could be adopted.

Conclusions and recommendations for future work

RCTs are a valuable source of information establishing the safety profile of medicinal products. Our review has demonstrated that data is not currently being fully utilised. Analysis of AE data is frequently inappropriate and reports often provide insufficient and inconsistent information to allow a comprehensive summary of the safety profile to be established. RCTs that have been published over a recent period in examples of high impact general medicine journals are deficient.

This research has identified two areas that would benefit from future research. i) Improving the consistency of reporting important AE outcomes across trials to facilitate comparison and synthesis. This is in line with work from the COMET Initiative group (http://www.comet-initiative.org/). The development of CORE safety outcomes by drug class could be considered.[7] ii) Evaluation of methods to analyse AEs in RCTs.

For peer review only - http://bmjopen.bmj.com/site/about/guidelines.xhtml

COMPETING INTERESTS

None declared.

FUNDING

This research was supported by the NIHR grant number DRF-2017-10-131.

DATA SHARING STATEMENT

No additional data are available.

AUTHOR STATEMENT

RP conceived the idea for this review, conducted the search, carried out data extraction and analysis, and wrote the manuscript. VC conceived the idea for the review, performed data extraction, critical revision of the manuscript and supervised the project. LH performed data extraction and critical revision of the manuscript. OS performed critical revision of the manuscript.

For peer review only

For peer review only - http://bmjopen.bmj.com/site/about/guidelines.xhtml

REFERENCES

1. Edwards IR and Biriell C. Harmonisation in pharmacovigilance. *Drug safety* 1994; 10: 93-102. DOI: 10.2165/00002018-199410020-00001.

2. The International Council for Harmonisation of Technical Requirements for Pharmaceuticals for Human Use (ICH). ICH Harmonised Tripartite Guideline. E2A Clinical Safety Data Management: Definitions and Standards for Expedited Reporting. 1994.

3. Ma H, Ke C, Jiang Q, et al. Statistical Considerations on the Evaluation of Imbalances of Adverse Events in Randomized Clinical Trials. *Therapeutic Innovation and Regulatory Science* 2015; 49: 957-965. DOI: http://dx.doi.org/10.1177/2168479015587363.

4. Food and Drug Administration. *Attachment B: Clinical Safety Review of an NDA or BLA of the Good Review Practice. Clinical Reveiw Template (MAPP 6010.3 Rev. 1)*. 2010.

5. Siddiqui O. Statistical methods to analyze adverse events data of randomized clinical trials. *Journal of Biopharmaceutical Statistics* 2009; 19: 889-899. DOI: http://dx.doi.org/10.1080/10543400903105463.

6. Tsang R, Colley L and Lynd LD. Inadequate statistical power to detect clinically significant differences in adverse event rates in randomized controlled trials. *Journal of Clinical Epidemiology* 2009; 62: 609-616.

7. Ioannidis JPA and Contopoulos-Ioannidis DG. Reporting of safety data from randomised trials. *The Lancet* 1998; 352: 1752-1753. DOI: 10.1016/S0140-6736(05)79825-1.

8. Edwards JE, McQuay HJ, Moore RA, et al. Reporting of Adverse Effects in Clinical Trials Should Be Improved. *Journal of Pain and Symptom Management* 1999; 18: 427-437. DOI: 10.1016/S0885-3924(99)00093-7.

9. Ioannidis JA and Lau J. Completeness of safety reporting in randomized trials: An evaluation of 7 medical areas. *JAMA* 2001; 285: 437-443. DOI: 10.1001/jama.285.4.437.

10. Pitrou I, Boutron I, Ahmad N, et al. Reporting of safety results in published reports of randomized controlled trials. *Archives of Internal Medicine* 2009; 169: 1756-1761. DOI: 10.1001/archinternmed.2009.306.

11. Cornelius VR, Sauzet O, Williams JE, et al. Adverse event reporting in randomised controlled trials of neuropathic pain: Considerations for future practice. *PAIN* 2013; 154: 213-220. DOI: 10.1016/j.pain.2012.08.012.

12. Maggi CB, Griebeler IH and Dal Pizzol Tda S. Information on adverse events in randomised clinical trials assessing drug interventions published in four medical journals with high impact factors. *Int J Risk Saf Med* 2014; 26: 9-22. 2014/05/07. DOI: 10.3233/JRS-140609.

13. Smith SM, Wang AT, Katz NP, et al. Adverse event assessment, analysis, and reporting in recent published analgesic clinical trials: ACTTION systematic review and recommendations. *PAIN* 2013; 154: 997-1008. DOI: 10.1016/j.pain.2013.03.003.

14. Peron J, Maillet D, Gan HK, et al. Adherence to CONSORT adverse event reporting guidelines in randomized clinical trials evaluating systemic cancer therapy: a systematic review. *J Clin Oncol* 2013; 31: 3957-3963. 2013/09/26. DOI: 10.1200/JCO.2013.49.3981.

15. Favier R and Crépin S. The reporting of harms in publications on randomized controlled trials funded by the "Programme Hospitalier de Recherche Clinique," a French academic funding scheme. *Clinical Trials* 2018; 0: 1740774518760565. DOI: 10.1177/1740774518760565.

16. Hum SW, Golder S and Shaikh N. Inadequate harms reporting in randomized control trials of antibiotics for pediatric acute otitis media: a systematic review. *Drug Safety* 2018 May 08. DOI: 10.1007/s40264-018-0680-0.

17. Ioannidis JA, Evans SW, Gøtzsche PC, et al. Better reporting of harms in randomized trials: An extension of the consort statement. *Annals of Internal Medicine* 2004; 141: 781-788. DOI: 10.7326/0003-4819-141-10-200411160-00009.

18. Lineberry N, Berlin JA, Mansi B, et al. Recommendations to improve adverse event reporting in clinical trial publications: A joint pharmaceutical industry/journal editor perspective. *BMJ (Online)* 2016; 355: i5078.
19. StataCorp. Stata Statistical Software: Release 15. College Station, TX: StataCorp LLC, 2017.
20. Litonjua AA, Carey VJ, Laranjo N, et al. Effect of Prenatal Supplementation With Vitamin D on Asthma or Recurrent Wheezing in Offspring by Age 3 Years: The VDAART Randomized Clinical Trial. *JAMA* 2016; 315: 362-370. 2016/01/28. DOI: 10.1001/jama.2015.18589.
21. Miller PD, Hattersley G, Riis BJ, et al. Effect of Abaloparatide vs Placebo on New Vertebral Fractures in Postmenopausal Women With Osteoporosis: A Randomized Clinical Trial. *JAMA* 2016; 316: 722-733. 2016/08/18. DOI: 10.1001/jama.2016.11136.
22. Libman IM, Miller KM, DiMeglio LA, et al. Effect of Metformin Added to Insulin on Glycemic Control Among Overweight/Obese Adolescents With Type 1 Diabetes: A Randomized Clinical Trial. *JAMA* 2015; 314: 2241-2250. 2015/12/02. DOI: 10.1001/jama.2015.16174.
23. Writing Committee for the Diabetic Retinopathy Clinical Research Network, Gross JG, Glassman AR, et al. Panretinal Photocoagulation vs Intravitreous Ranibizumab for Proliferative Diabetic Retinopathy: A Randomized Clinical Trial. *JAMA* 2015; 314: 2137-2146. 2015/11/14. DOI: 10.1001/jama.2015.15217.
24. Myles PS, Smith JA, Forbes A, et al. Stopping vs. Continuing Aspirin before Coronary Artery Surgery. *N Engl J Med* 2016; 374: 728-737. 2016/03/05. DOI: 10.1056/NEJMoa1507688.
25. Billings FTt, Hendricks PA, Schildcrout JS, et al. High-Dose Perioperative Atorvastatin and Acute Kidney Injury Following Cardiac Surgery: A Randomized Clinical Trial. *JAMA* 2016; 315: 877-888. 2016/02/26. DOI: 10.1001/jama.2016.0548.
26. Beardsley J, Wolbers M, Kibengo FM, et al. Adjunctive Dexamethasone in HIV-Associated Cryptococcal Meningitis. *N Engl J Med* 2016; 374: 542-554. 2016/02/11. DOI: 10.1056/NEJMoa1509024.
27. Kor DJ, Carter RE, Park PK, et al. Effect of Aspirin on Development of ARDS in At-Risk Patients Presenting to the Emergency Department: The LIPS-A Randomized Clinical Trial. *JAMA* 2016; 315: 2406-2414. 2016/05/18. DOI: 10.1001/jama.2016.6330.
28. Nichol A, French C, Little L, et al. Erythropoietin in traumatic brain injury (EPO-TBI): a double-blind randomised controlled trial. *The Lancet* 2015; 386: 2499-2506. DOI: 10.1016/s0140-6736(15)00386-4.
29. Aitken E, Jackson A, Kearns R, et al. Effect of regional versus local anaesthesia on outcome after arteriovenous fistula creation: a randomised controlled trial. *The Lancet* 2016; 388: 1067-1074. DOI: 10.1016/s0140-6736(16)30948-5.
30. Kaul U, Bangalore S, Seth A, et al. Paclitaxel-Eluting versus Everolimus-Eluting Coronary Stents in Diabetes. *N Engl J Med* 2015; 373: 1709-1719. 2015/10/16. DOI: 10.1056/NEJMoa1510188.
31. Cohen MS, Chen YQ, McCauley M, et al. Antiretroviral Therapy for the Prevention of HIV-1 Transmission. *N Engl J Med* 2016; 375: 830-839. 2016/07/19. DOI: 10.1056/NEJMoa1600693.
32. Isanaka S, Langendorf C, Berthe F, et al. Routine Amoxicillin for Uncomplicated Severe Acute Malnutrition in Children. *N Engl J Med* 2016; 374: 444-453. 2016/02/04. DOI: 10.1056/NEJMoa1507024.
33. Natalucci G, Latal B, Koller B, et al. Effect of Early Prophylactic High-Dose Recombinant Human Erythropoietin in Very Preterm Infants on Neurodevelopmental Outcome at 2 Years: A Randomized Clinical Trial. *JAMA* 2016; 315: 2079-2085. 2016/05/18. DOI: 10.1001/jama.2016.5504.
34. Sheehan WJ, Mauger DT, Paul IM, et al. Acetaminophen versus Ibuprofen in Young Children with Mild Persistent Asthma. *N Engl J Med* 2016; 375: 619-630. 2016/08/18. DOI: 10.1056/NEJMoa1515990.
35. Lind M, Hirsch IB, Tuomilehto J, et al. Liraglutide in people treated for type 2 diabetes with multiple daily insulin injections: randomised clinical trial (MDI Liraglutide trial). *BMJ* 2015; 351: h5364. 2015/10/30. DOI: 10.1136/bmj.h5364.

For peer review only

36. Lincoff AM, Mehran R, Povsic TJ, et al. Effect of the REG1 anticoagulation system versus bivalirudin on outcomes after percutaneous coronary intervention (REGULATE-PCI): a randomised clinical trial. *The Lancet* 2016; 387: 349-356. DOI: 10.1016/s0140-6736(15)00515-2.
37. Ribrag V, Koscielny S, Bosq J, et al. Rituximab and dose-dense chemotherapy for adults with Burkitt's lymphoma: a randomised, controlled, open-label, phase 3 trial. *The Lancet* 2016; 387: 2402-2411. DOI: 10.1016/s0140-6736(15)01317-3.
38. Nguyen QD, Merrill PT, Jaffe GJ, et al. Adalimumab for prevention of uveitic flare in patients with inactive non-infectious uveitis controlled by corticosteroids (VISUAL II): a multicentre, double-masked, randomised, placebo-controlled phase 3 trial. *The Lancet* 2016; 388: 1183-1192. DOI: 10.1016/s0140-6736(16)31339-3.
39. Rodes-Cabau J, Horlick E, Ibrahim R, et al. Effect of Clopidogrel and Aspirin vs Aspirin Alone on Migraine Headaches After Transcatheter Atrial Septal Defect Closure: The CANOA Randomized Clinical Trial. *JAMA* 2015; 314: 2147-2154. 2015/11/10. DOI: 10.1001/jama.2015.13919.
40. Ruperto N, Pistorio A, Oliveira S, et al. Prednisone versus prednisone plus ciclosporin versus prednisone plus methotrexate in new-onset juvenile dermatomyositis: a randomised trial. *The Lancet* 2016; 387: 671-678. DOI: 10.1016/s0140-6736(15)01021-1.
41. Whitehead KJ, Sautter NB, McWilliams JP, et al. Effect of Topical Intranasal Therapy on Epistaxis Frequency in Patients With Hereditary Hemorrhagic Telangiectasia: A Randomized Clinical Trial. *JAMA* 2016; 316: 943-951. 2016/09/07. DOI: 10.1001/jama.2016.11724.
42. Schulz KF, Altman DG, Moher D, et al. Consort 2010 statement: Updated guidelines for reporting parallel group randomized trials. *Annals of Internal Medicine* 2010; 152: 726-732. DOI: 10.7326/0003-4819-152-11-201006010-00232.
43. Lingvay I, Perez Manghi F, Garcia-Hernandez P, et al. Effect of Insulin Glargine Up-titration vs Insulin Degludec/Liraglutide on Glycated Hemoglobin Levels in Patients With Uncontrolled Type 2 Diabetes: The DUAL V Randomized Clinical Trial. *JAMA* 2016; 315: 898-907. 2016/03/05. DOI: 10.1001/jama.2016.1252.
44. Cornelius VR, Liu K, Peacock J, et al. Variation in adverse drug reactions listed in product information for antidepressants and anticonvulsants, between the USA and Europe: a comparison review of paired regulatory documents. *BMJ Open* 2016; 6. DOI: 10.1136/bmjopen-2015-010599.
45. Stephens MD, Talbot JC and Routledge PA. *The Detection of New Adverse Reactions*. 4 ed. London: Macmillan Reference 1998.
46. Bent S, Padula A and Avins AL. Brief communication: Better ways to question patients about adverse medical events: a randomized, controlled trial. *Annals of Internal Medicine* 2006; 144: 257-261. DOI: 10.7326/0003-4819-144-4-200602210-00007.
47. Davis S, Sun H and Jung K. Best practices for reporting safety data to data monitoring committees. *Trials Conference: 4th International Clinical Trials Methodology Conference , ICTMC and the 38th Annual Meeting of the Society for Clinical Trials United Kingdom* 2017; 18.
48. Furey A and Bechhofer R. Effective graphical analyses of adverse events in DMC reports. *Trials Conference: 4th International Clinical Trials Methodology Conference , ICTMC and the 38th Annual Meeting of the Society for Clinical Trials United Kingdom* 2017; 18.
49. Buhr KA, Downs M, Rhorer J, et al. Reports to Independent Data Monitoring Committees: An Appeal for Clarity, Completeness, and Comprehensibility. *Therapeutic Innovation & Regulatory Science* 2017: 2168479017739268. DOI: 10.1177/2168479017739268.
50. Amit O, Heiberger RM and Lane PW. Graphical approaches to the analysis of safety data from clinical trials. *Pharmaceutical Statistics* 2008; 7: 20-35.
51. Zink RC, Wolfinger RD and Mann G. Summarizing the incidence of adverse events using volcano plots and time intervals. *Clinical Trials* 2013; 10: 398-406.
52. Detry MA and Lewis RJ. The intention-to-treat principle: How to assess the true effect of choosing a medical treatment. *JAMA* 2014; 312: 85-86. DOI: 10.1001/jama.2014.7523.

53. Sample Closed Session DMC Report, https://www.biostat.wisc.edu/sites/default/files/Sample_Report_Closed_20160920.pdf (2016, accessed 26/03/2018).
54. Altman DG and Bland JM. Statistics notes: Absence of evidence is not evidence of absence. *BMJ* 1995; 311: 485. DOI: 10.1136/bmj.311.7003.485.
55. Jonville-Béra A, Giraudeau B and Autret-Leca E. Reporting of drug tolerance in randomized clinical trials: When data conflict with authors' conclusions. *Annals of Internal Medicine* 2006; 144: 306-307. DOI: 10.7326/0003-4819-144-4-200602210-00024.
56. Coovadia A, Abrams EJ, Strehlau R, et al. Efavirenz-Based Antiretroviral Therapy Among Nevirapine-Exposed HIV-Infected Children in South Africa: A Randomized Clinical Trial. *JAMA* 2015; 314: 1808-1817. 2015/11/04. DOI: 10.1001/jama.2015.13631.

For peer review only

Supplementary material

Table A1: Data items extracted from publications

			**Items collected**	**Instructions**
**Study details**		1	Study number	
		2	Journal	
		3	Funding source: public, private, both or unspecified.	Studies will be assumed to be funded by industry only if this is explicitly stated.
**Study characteristics**		4	Control: placebo, active or both	Select placebo if no active treatment is given, else active. Both should be selected for trials with multiple arms where there is at least one group receiving no active treatment and one group receiving an active treatment.
		5	Number of centres	
		6	Number randomised	
		7	Study duration (length of trial follow-up)	
**Methods**	Details of how AE outcomes were defined (coding, attribution) and were collected (mode of collection, timing)	8	Describe the collection method: passive surveillance, patient prompted, clinical examinations (e.g. vital signs or urine samples), and laboratory tests. (Select all that apply)	*Passive*: If authors state that AEs were collected throughout the study with no further information we will assume that collection was passive. *Prompted*: Prompted methods include, but are not limited to: questions about both specific events and AEs in general, questionnaires, or diaries.
		9	Stated the timing of collection.	
		10	Mention dictionary for coding of events: Researcher defined, MedDRA, CTCAE, WHO-ART, COSTART, ICD-10, other or not applicable	
		11	Describe who undertook the assessment of attribution to study drug: blinded assessor, unblinded assessor or not specified.	
**Planned analysis**	Details of any plans for analysing AE outcomes	12	Describe analysis for AE outcomes in the statistical methods.	Reference must be made to harmful events e.g. AEs or a specific harm event, this cannot be simply how binary events will be analysed.
		13	Define a 'safety' population for analysis.	
		14	Specify a planned interim analysis with stopping criteria: based on efficacy, based on safety, based on both efficacy and safety, yes but no other details given, no planned interim analysis or unclear	Criteria for stopping must be set out, it is not enough to say that the DMC reviewed the data.

For peer review only

Results	Details of what was reported and where	15	What was reported in the main paper: summaries of type of AEs (e.g. AE, SAE, AR, ADR), actual AE terms, both, neither or not applicable?	Not applicable is relevant when for example authors explicitly state that there are no events or there is only one event so summaries are inappropriate.
		16	What was reported in the appendix: summaries of type of AEs (e.g. AE, SAE, AR, ADR), actual AE terms, both, neither or not applicable?	Not applicable is relevant when for example authors explicitly state that there are no events or there is only one event so summaries are inappropriate.<br>We will only search the appendix/supplementary material for AE data if the main article makes reference to it.
		17	Who was the AE analysis performed on: all randomised, participants who took at least a single dose, other or not specified?	
		18	How were number of drop-outs/withdrawals reported: By treatment arm, overall, not reported or not applicable?	Not applicable is relevant when there are no drop-outs/withdrawals.<br>This does not include discontinuation of treatment.
		19	Were drop-outs/withdrawals due to AEs reported: Yes, no or not applicable?	Not applicable if drop-outs/withdrawals are not reported or if it is reported that there are no drop-outs/withdrawals.
		20	Were specific AEs causing withdrawals reported: Yes, no or not applicable?	Not applicable if drop-outs/withdrawals due to AEs are not reported or if it is reported that there are no drop-outs/withdrawals due to AEs.
		21	What was the selection criteria for the AEs reported?	Free text response where possibilities can include for example: most frequent, above a severity threshold, SAEs.<br>Include details of what's in the main journal article and what is in the appendix separately.
	Details of how AEs were summarised and presented - binary outcomes	22	What summary information was given: Number of people, number of events, both, unclear, not summarised or not applicable?	Only select 'number of events' if presented for each individual event not just overall number of events.<br>Not applicable is only relevant when report that there are no AEs.
		23	What analysis was performed: frequencies, percentages, differences and 95% confidence intervals, significance tests, other? (Select all that apply)	

For peer review only - http://bmjopen.bmj.com/site/about/guidelines.xhtml

	Details of how AEs were summarised and presented - continuous outcomes	24	Were continuous outcomes dichotomised: Yes for all, yes for some, no or not applicable?	This includes measures that will have been captured as continuous and then dichotomised for example blood levels, blood pressure etc.
		25	If continuous outcomes were analysed as continuous what analysis was performed: differences in measures of central tendency, significance tests, other? (Select all that apply)	
	Details of how AEs were summarised and presented	26	Were signal detection methods used?	
		27	Were any graphical summaries of AEs presented?	
		28	Were severity ratings given: Yes for all, yes for some, no or not applicable?	
		29	Were numbers of serious events presented: Yes by treatment arm, yes overall, no or not applicable?	If death is reported as part of the efficacy outcome it is not enough to constitute reporting serious events.
		30	Were serious events coded as treatment related: Yes for all, yes for some, no or not applicable?	
		31	Provided information on the duration of events?	This refers to the length of the actual AE i.e. how long did it last.
		32	Provided information on the timing of events?	This refers to the time of onset of the AE.
		33	Accounted for multiplicity of statistical tests?	
		34	Referenced CONSORT extension for harms?	

For peer review only - http://bmjopen.bmj.com/site/about/guidelines.xhtml

Table A2: Rationale for items extracted

Item	Rationale
How AE data was collected (mode of collection, timing) and defined (coding, attribution) during the study.	Variation in the collection and definition of events could explain differences in the incidence of observed events.[13, 14] For example specifically asking participants about an event of interest in one treatment group whilst relying on patient report in another is likely to lead to a disparity in incidence of events unlikely to be related to the medicinal product.
Assessment practices of severity of the event or relatedness to the medicinal product.	Attribution of causality by an unblinded assessor allows for subjectivity and bias (even if subconscious) to enter into their decision which can have important implications on the risk-benefit assessment.
Planned AE analysis (final and interim monitoring plans and analysis populations).	For example, the intention-to-treat population is likely to underestimate the AE risk by inflating the denominator. Therefore, this needs to be considered when making conclusions about a drug's safety profile.
How events were selected for inclusion in the journal article.	Due to the space constraints in journal articles it is not always feasible to report all AEs experienced by participants. Therefore, articles often only report a subset of AEs and how these are selected for inclusion has important implications for the safety evaluation. Arbitrary selection criteria can lead to inconsistencies in what is presented across trials for the same disease and/or drug. This prevents an accurate overview of the AEs experienced and invalidates any potential systematic review of events.
How and what summary event information was presented in the journal article.	For example, the number of events and duration of events provides insight into the impact of AEs, with repeated or longer events potentially having far wider clinical implications than a single, shorter event for both patients and prescribers.
How AEs were analysed.	There are many challenges to be considered when analysing AEs in clinical trials. For example, inappropriate statistical testing can lead to misleading conclusions e.g. failure to find a statistically significant result leading authors to conclude that the medicinal product is safe or chance imbalance could lead the authors to erroneously stopping a trial too early.[3-6]

For peer review only

For peer review only - http://bmjopen.bmj.com/site/about/guidelines.xhtml

Table A3: Examples of reporting practice in reviewed articles

Example no.	Study	Example practice	Example
1	Litonjua et al.[20]	Description of AE collection method	*"Study staff met with pregnant women monthly to administer a brief health questionnaire, assess medication use, and monitor for complications (via the questionnaire and medical record review)… After delivery, children were monitored by telephone every 3 months and in-person annually for 3 years, during which time infants' health, respiratory symptoms, and medications were assessed"*
2	Miller et al.[21]	Description of AE collection method	*"Safety evaluations included physical examinations, assessment of vital signs, clinical laboratory tests, and reporting of adverse events at each study visit"*
3	Libman et al.[22]	Description of planned AE analysis	*"The proportions of participants experiencing any adverse event, any related adverse event, any gastrointestinal event, any event other than a gastrointestinal event, at least 1 severe hypoglycaemic event, and at least 1 diabetic ketoacidosis event in each treatment group were compared using the Fisher exact test. The number of adverse events, new adverse events, serious adverse events, and non-serious adverse events were compared between groups using a Wilcoxon rank sumtest."*
4	Gross et al.[23]	Description of planned AE analysis	*"Safety analyses and secondary efficacy analyses used binomial regression, analysis of covariance, or the marginal Cox proportional hazards model as appropriate"*

For peer review only

Table A4: Stopping criteria for safety

Study	Main article text	Appendix text
Myles et al.[24]	"O'Brien–Fleming stopping boundaries were used to assess efficacy, and *a less stringent boundary was used to assess harm*."	
Billings et al.[25]	"The data and safety monitoring board (DSMB) reviewed patient recruitment practices, safety reporting, and data quality after 30 patients completed the study; performed an interim analysis after 277 patients … had completed the study to assess *safety of the intervention*; and performed a second interim analysis after 546 patients … had completed the study to assess the safety, efficacy, and futility of the intervention. The DSMB made recommendations based on qualitative assessments of the safety, efficacy, and futility of the intervention…"	"*Suspend enrolment in any study arm … due to safety concerns based on study intervention. Safety concerns include*: • Increase in in-hospital all-cause mortality in subjects randomized to A or B such that the DSMB deems the increase is excessive compared to A or B. • Increased treatment toxicity in either treatment group deemed excessive. Toxicity is defined as moderate or severe myalgias. • Increased severity of adverse events deemed "Probably Related" or "Possibly Related" to study intervention in either treatment group. Itemized adverse event reports separated by treatment will be provided. • Increased AKI incidence in either treatment group deemed excessive. • Increased incidence of stroke or hemodialysis requirement in either group (secondary endpoints) deemed excessive."
Beardsley et al.[26]	"An independent data and safety monitoring committee oversaw trial safety and analyzed unblinded data after every 50 deaths, according to its charter ..."	"The Haybittle-Peto boundary, requiring $p<0.001$ at interim analysis to consider stopping for efficacy, will be used as guidance. *A level of significance of 1% will be used as a guide for stopping the trial early because of a detected harm of dexamethasone*. In addition, the DMEC will receive conditional power curves to assess whether it remains realistic that the trial will demonstrate superiority of dexamethasone conditional on the data accrued up to the point of the interim analysis. Importantly, the DMEC recommendations will not be based purely on statistical tables but will also use clinical judgment."
Kor et al.[27]	"In addition to statistical criteria for significance, the study included a priori "go-no-go" definitions for recommending continuation to phase 3 study … Briefly, continuation to phase 3 would occur with a positive primary outcome finding along with an acceptable safety profile. An acceptable safety profile was defined as a serious adverse event profile for aspirin that was not statistically worse than placebo (95% CI for the relative risk of any serious adverse event covers the null value of relative risk = 1.0). The "no-go decision" was defined as early termination by the data and safety monitoring board for safety or unfavorable risk/benefit ratio. An indeterminate case in which there was a non–statistically significant effect but this effect was in a clinically meaningful direction was also defined."	Initiate Phase III Study: Demonstrated efficacy signal in addition to adequate safety profile Criteria: Early termination for benefit at interim analysis or $p<0.08885$ at final analysis (alpha=0.10 for study). *Serious adverse event profile of ASA not statistically worse than placebo (95% confidence interval for the relative risk of any SAE covers the null value of RR=1.0).* Further Development Potentially Required: Weak efficacy signal Criteria: Primary endpoint did not achieve a priori level of significance but there were at least a general consistency of secondary endpoints indicating propensity for efficacy with a larger sample size and/or more specific primary endpoint. Abandon Treatment Platform: Harm (in efficacy or safety endpoints) Criteria: Study terminated early per recommendation *by DSMB for safety and/or risk/benefit ratio concerns* (i.e., stop for futility, harm, unacceptable risk profile, etc.)

For peer review only - http://bmjopen.bmj.com/site/about/guidelines.xhtml

Nichol et al.[28]	We used a group sequential statistical approach to do two equally spaced pre-planned interim analyses (at 33% and 67% of total recruitment) to assess accumulated safety data (differential proportions of deep venous thrombosis and total mortality). This *approach was chosen to provide for early stopping for probable harm* or strong evidence of benefit. We applied the Haybittle-Peto criterion ($\|Zk\| \geq 3$) for early stopping at these analyses.	

For peer review only

For peer review only - http://bmjopen.bmj.com/site/about/guidelines.xhtml

Table A5: Selection criteria used to select AEs presented in the main journal report

Selection criteria	n	%
All AEs presented	20	10.87
AEs in greater than x% in any group	10	5.43
AEs in greater than x% in treatment group	4	2.17
AEs in greater than x% in all patients	1	0.54
Most common (no criteria specified)	9	4.89
Predefined AEs	26	14.13
SAEs	15	8.15
AEs leading to study drug discontinuation/interruption	3	1.63
Treatment related AEs	5	2.72
Grade 3>= events	9	4.89
AEs in greater than x% in any group & predefined/special interest AEs	4	2.17
AEs in greater than x% in any group & frequency between groups differed by more than y% & predefined/special interest AEs	1	0.54
AEs in greater than x% in all patients & predefined/special interest AEs	3	1.63
AEs in greater than x% in treatment group & AEs of special interest	2	1.09
AEs in greater than x% in any group & all SAEs	2	1.09
AEs in greater than x% in all patients & all SAEs	1	0.54
AEs in greater than x% in any group & SAEs related to treatment	1	0.54
Most common (no criteria specified) & predefined/special interest AEs	3	1.63
Most common (no criteria specified) & all SAEs	4	2.17
Most common (no criteria specified) & all SAEs & AEs leading to study drug discontinuation/interruption	1	0.54
Most common (no criteria specified) & treatment related SAEs	1	0.54
AEs where frequency between groups differed by more than y% & all SAEs	1	0.54
AEs of special interest	6	3.26
Grade >=3 AEs in greater than x% of patients	1	0.54
Grade >=3 AEs in greater than x% in intervention & y% in control	1	0.54
Most common (no criteria specified) grade 3>= AEs	1	0.54
Most common SAEs (no criteria specified)	1	0.54
SAEs & AE of special interest	1	0.54
Treatment related AEs in greater than x% of patients	1	0.54
Treatment related AEs in greater than x% in any group	1	0.54
AEs in greater than x% in treatment group & SAEs	1	0.54
AEs in greater than x% in treatment group & SAEs & predefined AEs	2	1.09
AEs in greater than x% in any group & significantly different & SAEs	1	0.54
AEs in greater than x% in any group & treatment related AEs/SAEs	2	1.09
AEs in greater than x% in treatment group & treatment related AEs & SAEs	1	0.54
AEs in greater than x% in treatment group & treatment related AEs in greater than y% in all patients	1	0.54
AEs in greater than x% in any group & Grade 3>= events	1	0.54
AEs in greater than x% in all patients & Grade 3>= events	1	0.54
AEs in greater than x% in all patients & Grade 2>= treatment related AEs	1	0.54
AEs in greater than x% in any group & Grade 3>= events in greater than y% in any group	1	0.54
AEs in greater than x% in any group & SAEs in treatment group	1	0.54
AEs in greater than x% in any group & AEs of special interest & most common (no criteria specified) AEs leading to treatment discontinuation/interruption & predefined AEs	1	0.54
AEs in greater than x% in any group, AEs of special interest in greater than y% in treatment group & treatment related deaths	1	0.54

For peer review only - http://bmjopen.bmj.com/site/about/guidelines.xhtml

AEs in greater than x% in treatment group & SAEs in greater than y% in any group	1	0.54
AEs and SAEs occurring more often in treatment group than control	1	0.54
AEs in greater than x% in treatment group & occurred more often in treatment group than control & predefined/special interest AEs	1	0.54
AEs in greater than x% in any group & frequency between groups differed by more than y%, SAEs in greater than z% in any group & all grade >=3 AEs	1	0.54
AEs in greater than x% patients & more than y% difference between treatment groups & AEs leading to treatment discontinuation/interruption & most common SAEs (no criteria specified) & death	1	0.54
Predefined AEs, AEs leading to hospitalisation/death/study drug discontinuation/interruption & SUSARS	2	1.09
Some form of overall summary	6	3.26
Not specified how selected	6	3.26
Not summarised in main paper	11	5.98

For peer review only

For peer review only - http://bmjopen.bmj.com/site/about/guidelines.xhtml

Table A6: Selection criteria used to select AEs presented in the appendix

Selection criteria	n	%
All AEs	18	9.78
SAEs	18	9.78
All AEs & SAEs	4	2.17
AEs in greater than x% in any group	7	3.8
AEs in greater than x% in treatment group	2	1.09
AEs in greater than x% in all patients	1	0.54
AEs in greater than x% in any group & all SAEs	2	1.09
AEs in greater than x% in treatment group & all SAEs	1	0.54
AEs in greater than x% in all patients & all SAEs	3	1.63
AEs in greater than x% in treatment group & all SAEs	1	0.54
AEs in greater than x% in treatment group & greater than in control group & all SAEs	1	0.54
SAEs in greater than x% in any group	1	0.54
AEs in greater than x% in any group & SAEs in greater than y% in any group	1	0.54
AEs in greater than x% in any group & AEs of special interest	2	1.09
Treatment related AEs	5	2.72
Treatment related AEs in greater than x% in any group	2	1.09
Grade 3>= events	2	1.09
Predefined AEs	8	4.35
AEs of special interest	1	0.54
AEs leading to study drug discontinuation/interruption	2	1.09
AEs leading to study drug discontinuation & SAEs	1	0.54
Grade 3>= events leading to study drug discontinuation & grade 3>= laboratory results	1	0.54
Treatment related AEs & AEs leading to study drug discontinuation	1	0.54
AEs in greater than x% in all patients leading to treatment discontinuations, SAEs in greater than x% in any group, serious predefined/special interest AEs and clinically significant laboratory results	1	0.54
AEs in greater than x% in any group, treatment related AEs in greater than x% in any group, treatment related SAEs and select AEs	1	0.54
Clinical laboratory data	1	0.54
Predefined AEs, AEs leading to hospitalisation/death/study drug discontinuation/interruption & SUSARS	3	1.63
Deaths	2	1.09
Some form of overall summary	5	2.72
Not specified how selected	2	1.09
Not summarised in the appendix	84	45.65

For peer review only - http://bmjopen.bmj.com/site/about/guidelines.xhtml

Table A7: Population used for AE analysis

Analysis population	n	%
Those that took at least a single dose	75	40.76
All randomised	54	29.35
Randomised and not withdrawn/ineligible	19	10.33
Not specified	17	9.24
Not applicable	3	1.63
Took a single dose and underwent AE/toxicity assessment	3	1.63
Active treatment groups	2	1.09
Completed treatment and assessed for primary outcome	2	1.09
Other	2	1.09
Patients who treatment was at least attempted on	1	0.54
Intention-to-treat population	1	0.54
Randomised and assessed for primary outcome	1	0.54
Randomised and attended at least on follow-up visit	1	0.54
Randomised and remained in follow-up	1	0.54
Randomised and underwent AE/toxicity assessment	1	0.54
Randomised, eligible and received at least a single dose	1	0.54

For peer review only

For peer review only - http://bmjopen.bmj.com/site/about/guidelines.xhtml

Reporting checklist for systematic review and meta-analysis.

Based on the PRISMA guidelines.

Instructions to authors

Complete this checklist by entering the page numbers from your manuscript where readers will find each of the items listed below.

Your article may not currently address all the items on the checklist. Please modify your text to include the missing information. If you are certain that an item does not apply, please write "n/a" and provide a short explanation.

Upload your completed checklist as an extra file when you submit to a journal.

In your methods section, say that you used the PRISMA reporting guidelines, and cite them as:

Moher D, Liberati A, Tetzlaff J, Altman DG, The PRISMA Group. Preferred Reporting Items for Systematic Reviews and Meta-Analyses: The PRISMA Statement

		Reporting Item	Page Number
	#1	Identify the report as a systematic review, meta-analysis, or both.	1
Structured summary	#2	Provide a structured summary including, as applicable: background; objectives; data sources; study eligibility criteria, participants, and interventions; study appraisal and synthesis methods; results; limitations; conclusions and implications of key findings; systematic review registration number	2
Rationale	#3	Describe the rationale for the review in the context of what is already known.	5-6
Objectives	#4	Provide an explicit statement of questions being addressed with reference to participants, interventions, comparisons, outcomes, and study design (PICOS).	6

For peer review only

Protocol and registration	#5	Indicate if a review protocol exists, if and where it can be accessed (e.g., Web address) and, if available, provide registration information including the registration number.	n/a - review protocol was not published
rEEligibility criteria	#6	Specify study characteristics (e.g., PICOS, length of follow-up) and report characteristics (e.g., years considered, language, publication status) used as criteria for eligibility, giving rational	6-7
Information sources	#7	Describe all information sources in the search (e.g., databases with dates of coverage, contact with study authors to identify additional studies) and date last searched.	6
Search	#8	Present full electronic search strategy for at least one database, including any limits used, such that it could be repeated.	n/a - we manually searched the electronic contents table of the journals for reports of original RCTs
Study selection	#9	State the process for selecting studies (i.e., for screening, for determining eligibility, for inclusion in the systematic review, and, if applicable, for inclusion in the meta-analysis).	7
Data collection process	#10	Describe the method of data extraction from reports (e.g., piloted forms, independently by two reviewers) and any processes for obtaining and confirming data from investigators.	7
Data items	#11	List and define all variables for which data were sought (e.g., PICOS, funding sources), and any assumptions and simplifications made.	7

For peer review only

Risk of bias in individual studies	#12	Describe methods used for assessing risk of bias in individual studies (including specification of whether this was done at the study or outcome level, or both), and how this information is to be used in any data synthesis.	n/a – the review was to identify current practice, we did not look at and synthesize the actual results of individual studies and as such this assessment was not relevant
Summary measures	#13	State the principal summary measures (e.g., risk ratio, difference in means).	8
Planned methods of analyis	#14	Describe the methods of handling data and combining results of studies, if done, including measures of consistency (e.g., I2) for each meta-analysis.	8
Risk of bias across studies	#15	Specify any assessment of risk of bias that may affect the cumulative evidence (e.g., publication bias, selective reporting within studies).	n/a – please see item #12
Additional analyses	#16	Describe methods of additional analyses (e.g., sensitivity or subgroup analyses, meta-regression), if done, indicating which were pre-specified.	n/a – no such analysis was performed
Study selection	#17	Give numbers of studies screened, assessed for eligibility, and included in the review, with reasons for exclusions at each stage, ideally with a flow diagram.	n/a – manual search resulted in only eligible articles being downloaded
Study characteristics	#18	For each study, present characteristics for which data were extracted (e.g., study size, PICOS, follow-up period) and provide the citation.	9
Risk of bias within studies	#19	Present data on risk of bias of each study and, if available, any outcome-level assessment (see Item 12).	n/a – please see item #12
Results of individual studies	#20	For all outcomes considered (benefits and harms), present, for each study: (a) simple summary data for each intervention group	n/a – only simple descriptive statistics are presented to describe current practice

For peer review only

		and (b) effect estimates and confidence intervals, ideally with a forest plot.	
Synthesis of results	#21	Present the main results of the review. If meta-analyses are done, include for each, confidence intervals and measures of consistency.	9-13
Risk of bias across studies	#22	Present results of any assessment of risk of bias across studies (see Item 15).	n/a – please see item #12
Additional analysis	#23	Give results of additional analyses, if done (e.g., sensitivity or subgroup analyses, meta-regression [see Item 16]).	n/a – please see item #16
Summary of Evidence	#24	Summarize the main findings, including the strength of evidence for each main outcome; consider their relevance to key groups (e.g., health care providers, users, and policy makers	13-21
Limitations	#25	Discuss limitations at study and outcome level (e.g., risk of bias), and at review level (e.g., incomplete retrieval of identified research, reporting bias).	26/27
Conclusions	#26	Provide a general interpretation of the results in the context of other evidence, and implications for future research.	27
Funding	#27	Describe sources of funding or other support (e.g., supply of data) for the systematic review; role of funders for the systematic review.	28

The PRISMA checklist is distributed under the terms of the Creative Commons Attribution License CC-BY. This checklist was completed on 29. May 2018 using http://www.goodreports.org/, a tool made by the EQUATOR Network in collaboration with Penelope.ai

For peer review only

BMJ Open

Analysis and reporting of adverse events in randomised controlled trials: a review

Journal:	*BMJ Open*
Manuscript ID	bmjopen-2018-024537.R3
Article Type:	Research
Date Submitted by the Author:	15-Jan-2019
Complete List of Authors:	Phillips, Rachel; Imperial College London, Faculty of Medicine, School of Public Health<br>Hazell, Lorna; Drug Safety Research Unit, Clinical Research; University of Portsmouth, Associate Dept. of Pharmacy and Biomedical Sciences<br>Sauzet, Odile; Universität Bielefeld,<br>Cornelius, Victoria; Imperial College London Faculty of Medicine, School of Public Health
**Primary Subject Heading**:	Research methods
Secondary Subject Heading:	Medical publishing and peer review
Keywords:	Randomised controlled trials, Harm data, Adverse drug reactions, Systematic review, Investigational drug, Adverse events < THERAPEUTICS

SCHOLARONE™
Manuscripts

Analysis and reporting of adverse events in randomised controlled trials: a review

Authors:

Rachel Phillips* - Imperial College London, London, United Kingdom

Lorna Hazell – Drug Safety Research Unit, Southampton, United Kingdom

Odile Sauzet – Bielefeld University, Bielefeld, Germany

Victoria Cornelius - Imperial College London, London, United Kingdom

* Corresponding author

Address: Imperial Clinical Trials Unit, Imperial College London, 1st Floor Stadium House, 68 Wood Lane, London, W12 7RH

Email: r.phillips@imperial.ac.uk

Telephone: 020 759 49356

Word Count: 4362

For peer review only

For peer review only - http://bmjopen.bmj.com/site/about/guidelines.xhtml

Abstract

Objective

To ascertain contemporary approaches to the collection, reporting and analysis of adverse events (AEs) in randomised controlled trials (RCTs) with a primary efficacy outcome.

Design

A review of clinical trials of drug interventions from four high impact medical journals.

Data sources

Electronic contents table of the BMJ, the Journal of the American Medical Association, the Lancet, and the New England Journal of Medicine were searched for reports of original RCTs published between September 2015 and September 2016.

Methods

A pre-piloted checklist was used and single data extraction was performed by three reviewers with independent check of a randomly sampled subset to verify quality. We extracted data on collection methods, assessment of severity and causality, reporting criteria, analysis methods and presentation of AE data.

Results

We identified 184 eligible reports (BMJ n=3; JAMA n=38, Lancet n=62; and NEJM n=81). Sixty-two percent reported some form of spontaneous AE collection but only 29% included details of specific prompts used to ascertain AE data. Numbers that withdrew from the trial were well reported (80%),

For peer review only - http://bmjopen.bmj.com/site/about/guidelines.xhtml

however only 35% of these reported whether withdrawals were due to AEs. Results presented and analysis performed was predominantly on 'patients with at least 1 event' with 84% of studies ignoring repeated events. Despite a lack of power to undertake formal hypothesis testing, 47% performed such tests for binary outcomes.

Conclusions

This review highlighted that the collection, reporting and analysis of AE data in clinical trials is inconsistent and RCTs as a source of safety data are underutilised. Areas to improve include reducing information loss when analysing at patient level and inappropriate practice of underpowered multiple hypothesis testing. Implementation of standard reporting practices could enable a more accurate synthesis of safety data and development of guidance for statistical methodology to assess causality of AEs could facilitate better statistical practice.

Keywords

Randomised controlled trials; adverse events; harm data; adverse drug reactions; review; investigational drug.

For peer review only

For peer review only - http://bmjopen.bmj.com/site/about/guidelines.xhtml

Article Summary

Strengths and Limitations of this study

1. This is the first review to examine and quantify the methods used for AE analysis in RCTs published in high impact general medical journals.
2. This review identifies methodological weakness that need to be addressed as well as good practice that could be adopted.
3. Articles included in this review were published in four of the top ranked general medical journals therefore results are likely to be biased towards better practice.
4. Included articles are only for year 2015-2016 and as such may not reflect current practice.

For peer review only

For peer review only - http://bmjopen.bmj.com/site/about/guidelines.xhtml

INTRODUCTION

The methods to analyse and report outcomes to measure benefit from randomised controlled trials (RCTs) are well developed but this progress has not been matched for adverse event (AE) outcomes. An adverse event is defined as 'any untoward medical occurrence that may present during treatment with a pharmaceutical product but which does not necessarily have a causal relationship with this treatment'.[1] An adverse drug reaction (ADR) is defined as 'a response to a drug which is noxious and unintended …' where a causal relationship is 'at least a reasonable possibility'.[1, 2] RCTs provide an opportunity to compare rates of AEs between arms allowing causality to be evaluated. However, contemporary analysis and reporting practices are inadequate.

There are many challenges associated with analysing and reporting AEs in clinical trials. RCTs are typically designed to determine the efficacy of an intervention but are often underpowered to detect important differences in AEs between arms which may suggest an ADR. Often large numbers of AEs are reported during a study, sometimes exceeding the number of patients in the clinical trial. Performing hypothesis tests on these AEs would lead to issues of multiplicity, however any adjustment for multiplicity would make a 'finding untenable'.[3, 4] The use of hypothesis testing may result in the medicinal product being deemed unsafe and a trial being halted too early due to a chance imbalance, or conversely deemed safe and not stopped early enough resulting in more patients than necessary suffering an ADR.[3, 5, 6] Unlike efficacy outcomes which are well defined and restricted in number at the planning stage of a RCT, we collect numerous, undefined AEs in RCTs. Furthermore, AE collection requires additional information to be obtained on factors such as severity, timing and duration, number of occurrences and outcome, which for our efficacy outcomes would have all been predefined.

For peer review only - http://bmjopen.bmj.com/site/about/guidelines.xhtml

Previous studies have examined the methods for AE collection and presentation only, and highlighted the inadequacies in AE reporting in journal articles.[7-16] In 2004 the Consolidated Standards of Reporting Trials (CONSORT) Group produced an extension to their guidelines for reporting trial results to cover the reporting of harms, however implementation of these guidelines has been shown to be poor.[10, 14-17] Recently a joint pharmaceutical/journal collaboration published practical guidance and examples on what should be reported in journal articles and how it should be displayed to ensure transparency and aid clinical interpretation. They promote the use of clinical judgement in reporting rather than mandatory guidance.[18] Whilst this work has been undertaken there remains uncertainty about practice for reporting and presenting AE data, and in addition the analysis practice for AEs remains a neglected area for review.

The aim of this review was to evaluate contemporary practice for collection, reporting and analysis of AEs in RCTs where the primary outcome was efficacy. The aim being to identify and promote any areas of good practice, whilst highlighting any areas for improvement.

METHODS

Search strategy

The top four general medical journals as ranked by impact factors that publish clinical trials of drug interventions were selected: The BMJ (Impact Factor 20.79), the Journal of the American Medical Association (JAMA, IF 44.41), the Lancet (IF 47.83), and the New England Journal of Medicine (NEJM, IF 72.41). Impact factors quoted are from 2016 to reflect the time period from which the articles were drawn. High impact journals were chosen as we would expect practice in these journals to be of high standard as they include statistical and methodological review. We limited the search to four

For peer review only - http://bmjopen.bmj.com/site/about/guidelines.xhtml

journals after an initial scoping review revealed around 100 studies would be eligible for inclusion, which was a feasible number to review given the time and resources available and would provide a sufficient number to evaluate practice. One reviewer manually searched the electronic contents table of the journals for reports of original RCTs published between September 2015 and September 2016, inclusive. Any queries regarding eligibility were reviewed and discussed with a second reviewer.

Selection criteria

The inclusion criteria were phase II-IV RCTs of drug interventions where the primary outcome was efficacy of the intervention. We did not restrict according to number of treatment arms and included both parallel and cluster RCTs. We excluded cross-over RCTs, RCTs with adaptive randomisation, observational studies, case reports, editorials and letters. We also excluded RCTs where the intervention was not a drug product (i.e. not classified as a clinical trial of an investigational medicinal product (CTIMP)). As the study aimed to assess how authors report and analyse AEs in studies where the primary outcome was efficacy, trials that were specifically designed to investigate safety as a primary outcome were not included.

Data extraction

Potentially eligible articles were identified based on titles and abstracts and the full text of these studies were retrieved. Supplementary material was also reviewed if readers were referred here from the main article for further results. Supplementary Table A1 lists all data items captured with guidance given to the reviewers for extraction. The items to be extracted were based on the work by

For peer review only - http://bmjopen.bmj.com/site/about/guidelines.xhtml

Cornelius et al. and the CONSORT harm extension with additional items added to capture more specific information on analysis practices.[11, 17] Specifically we focused on the following areas: how AE data was collected (mode of collection, timing) and defined (coding, attribution); how AEs were assessed in terms of severity of the event or relatedness to the medical intervention; if there was any planned AE analysis (final and interim monitoring plans and analysis populations); how events were selected for inclusion in the journal article; how summary event information was presented in the journal article and how AEs were analysed.[11] A more detailed rationale for the choice of items extracted is provided in the supplementary material (Table A2).

A data extraction sheet was piloted and then single data extraction was performed by three reviewers (RP, VC and LH) with 10% independent check of a randomly sampled subset to verify quality. Queries were also informally discussed between reviewers on an ongoing basis. Where specific items were flagged for poor agreement these were re-extracted. Any queries during data extraction were shared and disagreements between reviewers were resolved through discussion.

Data analysis

The proportion of trials reporting each item, 3-4 and 8-34 in supplementary Table A1 were calculated and summary statistics (median and ranges) were calculated for items 5-7. All analyses were performed in Stata version 15.[19] A risk of bias assessment was not undertaken as this study aimed to describe best practice and not evaluate outcomes.

Patient and public involvement

This review forms part of a wider research project that was developed with input from a range of patient representatives. There were no study participants directly involved in this review but the

For peer review only - http://bmjopen.bmj.com/site/about/guidelines.xhtml

original proposal and patient and public involvement (PPI) strategy were reviewed by service user representatives (with experience as clinical trial participants and PPI advisors) who provided advice specifically with regard to communication and dissemination to patient and public groups.

RESULTS

Data extraction

A total of 585 items were extracted twice across all three reviewers to check the quality of the data extraction. A total of 95 discrepancies were identified. This gave agreement of 84%. During this independent check several items were flagged for potential poor agreement. These items were 100% independently extracted by one author and verified. The items were: study duration; the AE collection method; timing of collection; how binary harm outcomes were summarised; whether continuous outcomes were dichotomised; if continuous outcomes were left as continuous how they were analysed.

Study characteristics

The search identified 184 eligible trial reports (BMJ n=3; JAMA n=38, Lancet n=62; and NEJM n=81) in which a total of 496911 participants were randomised with a median of 556 participants per trial (range 30, 205513; interquartile range (IQR) 281, 1704). The median trial follow-up was 52 weeks (range 48 hours to 10 years; IQR 24, 104 weeks) and 93% were multi-centre trials. Fifty-percent of

For peer review only - http://bmjopen.bmj.com/site/about/guidelines.xhtml

studies had an active comparator and over 50% of trials received some element of industry funding (Table 1).

Table 1: Characteristics of included studies

Characteristic	N=184		
	**Median**	**(IQR)**	**min, max**
**Sample size**	556	(281, 1704)	30, 205513
**Centres**[a]	35	(12, 100)	1, 1368
**Trial duration (weeks)**[b]	52	(24, 104)	0.3, 521
	**n**	**%**	
**Journal**			
**BMJ**	3	1.6	
**JAMA**	81	44.0	
**Lancet**	38	20.7	
**NEJM**	62	33.7	
**Funded by**			
**Public**	70	38.3	
**Industry**	80	43.7	
**Both**	33	18.0	
**Centre**			
**Single-centre**	12	7.0	
**Multi-centre**	161	93.0	
**Control**			
**Placebo**	95	51.6	
**Active**	80	43.5	
**Both**	8	4.4	
**Neither**[c]	1	0.5	

Abbreviations: *IQR = Inter-quartile range; min = minimum; and max = maximum*

[a]11 reports did not specify the number of centres

[b]2 reports did not specify trial duration

[c]One trial compared interventional drug to behavioural change intervention

Collection and assessment methods

For peer review only

Sixty-two percent (n=114) of reports made reference to some form of passive (e.g. spontaneously reported by patients) AE monitoring or collection methods. Of these only 46.5% (53/114) or 29% of total reports included specific details (prompts e.g. questions about specific events or AEs in general, questionnaires, or diaries) regarding these collection methods (Supplementary Table A3, examples 1-2).[20, 21] The timing of collection was well documented (91%, 48 out of 53 reports) in the reports that included specific details about the prompts used to collect AEs. Although specific details on clinical examinations (e.g. vital signs and blood pressure) and laboratory tests were not widely reported (only 57% of reports (95 out of 166 reports with clinical examinations and/or laboratory results presented) included details on the timing of such assessments) it was often clear from the results presented that participants had undergone these assessments (83% and 79% of studies reported clinical and laboratory results respectively) (Table 2).

For peer review only

For peer review only - http://bmjopen.bmj.com/site/about/guidelines.xhtml

Table 2: Collection, assessment and analysis methods reported by studies

Section	Component	Data item	N=184	
**Collection**			n	%
	**How was AE/harm information collected?**			
		Passive collection	114	62.0
		Prompted collection (n=114)	53	46.5
		No method of collection reported	70	38.0
	**Did they undertake proactive screening?**			
		Clinical examinations	153	83.2
		Laboratory tests	146	79.4
	**Timing of prompted collection specified** (n=53)		48	90.6
	**Timing of active collection specified** (n=166)		95	57.2
	**Which, if any, dictionary was used to code AE data?**			
		CTCAE	18	9.8
		MedDRA	43	23.4
		CTCAE and MedDRA	1	0.5
		DAIDS	2	1.1
		ICD-10	1	0.5
		Researcher defined	2	1.1
		Other	3	1.6
		No dictionary reported	114	62.0
**Assessment**				
	**Who assigned attribution to study drug?**			
		Blinded assessor	9	4.9
		Unblinded assessor	7	3.8
		Both	1	0.5
		Not specified	164	89.1
		Not applicable[a]	3	1.6
**Analysis**				
	**Was any analysis for AEs specified in the methods section?**			
		Yes	57	31.0
	**Was a population for AE analysis specified?**			
		Yes	82	44.6
	**Was there a planned interim analysis with stopping criteria?**			
		No	138	75.0
		Yes for efficacy	24	13.0
		Yes for efficacy & futility	11	6.0
		Yes for efficacy & safety	3	1.6
		Yes for efficacy, futility & safety	2	1.1
		Yes but no other details given	6	3.3

Abbreviations: *AE = Adverse event; CTCAE = Common Terminology Criteria for Adverse Events; MedDRA = Medical Dictionary for Regulatory Activities; DAIDS = The Division of AIDS; and ICD-10 = International Classification of Diseases 10th revision.*

NOTE: Denominator specified in item column if it differs from total sample

[a]3 reports made no reference to AE data throughout the article

For peer review only - http://bmjopen.bmj.com/site/about/guidelines.xhtml

Prespecified analysis

Thirty-one percent of reports provided information on the planned analysis for AEs in the statistical analysis section of the paper and 45% pre-specified a safety population (Supplementary Table A3, examples 3-4 and Table 2).[22, 23] A quarter of trials reported planned interim analysis with stopping criteria (Table 2), five (2.7%) of which included specific criteria on stopping for a harmful event (Supplement Table A4[24-28]).

Selection of AEs and reporting practices

Two reports only made generic statements regarding AE data: "*there were no significant adverse events related to the procedure*" and "*no excess in mortality or major adverse events were found…*". Three reports made no mention of AEs throughout the manuscript.[29-33]

Twenty-four (13%) trials only provided a summary of the number of AEs or serious AEs rather than listing the actual AEs that occurred. For example "*Six serious adverse events occurred in the acetaminophen group and 12 in the ibuprofen group.*"[34] Of these 24 trials, 10 did provide specific details of the types of events in an appendix. This means 8% of trials either did not report AEs or only included a summary (Table 3).

For peer review only - http://bmjopen.bmj.com/site/about/guidelines.xhtml

Table 3: Summaries of results presented by studies

Component	Data item	N=184	
		n	%
**What was reported in the manuscript?**			
	Actual AE terms	73	39.7
	Summaries of AE type (e.g. AE, SAE)	24	13.0
	Both	80	43.5
	Neither	7	3.8
**What was reported in the appendix?**			
	Actual AE terms	76	41.3
	Summaries of AE type (e.g. AE, SAE)	7	3.8
	Both	22	12.0
	Neither	3	1.6
	Not applicable[a]	76	41.3
**Which population was the AE analysis performed on?**			
	All randomised	54	29.4
	Those that took at least a single dose	75	40.8
	Other	35	19.0
	Not specified	17	9.2
	Not applicable[b]	3	1.6
**Were drop-outs/withdrawals reported?**			
	No	33	17.9
	Yes by treatment arm	144	78.3
	Yes overall	2	1.1
	Not applicable[c]	5	2.7
**Were withdrawals due to AEs reported?** (n=146)			
	No	89	61.0
	Yes	51	34.9
	Not applicable[d]	6	4.1
**Were specific AEs causing withdrawals reported?** (n=51)			
	No	39	76.5
	Yes	12	23.5
**How were binary AE outcomes summarised by arm?**			
	Not summarised[e]	6	3.3
	Number of people with an event	154	83.7
	Number of events	11	6.0
	Both	12	6.5
	Unclear	1	0.5
**Were frequencies of AEs reported by arm?**			
	No	5	2.7
	Yes for some	13	7.1
	Yes for all	160	87.0
	Not applicable[e]	6	3.3
**Were percentages of AEs reported by arm?**			
	No	18	9.8
	Yes for some	25	13.6

For peer review only - http://bmjopen.bmj.com/site/about/guidelines.xhtml

For peer review only

	Yes for all	135	73.4
	Not applicable[e]	6	3.3
**Were between arm differences and 95% CI of AEs reported?**			
	No	141	76.6
	Yes for some	18	9.8
	Yes for all	19	10.3
	Not applicable[e]	6	3.3
**Were statistical significance tests between arms on AEs reported?**			
	No	92	50.0
	Yes for some	31	16.9
	Yes for all	55	29.9
	Not applicable[e]	6	3.3
**Were continuous AEs outcomes dichotomised for summaries?**			
	No	10	5.4
	Yes for some	28	15.2
	Yes for all	108	58.7
	Not applicable	38	20.7
**If continuous outcomes were left as continuous what between arm analyses was performed?** (n=38)			
**Differences in measures of central tendency estimated with 95% CI**			
	No	23	60.5
	Yes for some	1	2.6
	Yes for all	14	36.8
**Between arm hypothesis tests performed**			
	No	12	31.6
	Yes for some	2	5.3
	Yes for all	24	63.2
**Were any 'signal detection' approaches used?**			
	No	184	100.0
	Yes	0	0.0
**Were there any graphical presentations of AE outcomes?**			
	No	162	88.0
	Yes	22	12.0
**Were summaries of severity rating of AEs reported?**			
	No	103	56.0
	Yes for some	41	22.3
	Yes for all	35	19.0
	Not applicable[f]	5	2.7
**Were number of serious AEs reported?**			
	No	44	23.9
	Yes overall	2	1.1
	Yes by treatment arm	132	71.7
	Not applicable[g]	6	3.3
**For serious AEs was relatedness given?** (n=134)			
	No	77	57.5
	Yes for some	18	13.4
	Yes for all	38	28.4

For peer review only - http://bmjopen.bmj.com/site/about/guidelines.xhtml

For peer review only

	Yes overall	1	0.8
**Were there any AEs where information on duration of events was reported?**			
	No	175	95.1
	Yes	9	4.9
**Were there any AEs where information on the time of occurrence of events was reported?**			
	No	132	71.7
	Yes	52	28.3
**If any significance tests were performed on AEs was multiplicity of events accounted for?**			
	No	81	44.0
	Yes	3	1.6
	Not applicable	100	54.4
**Did the report reference the CONSORT extension to harms**			
	No	184	100.0
	Yes	0	0.0

Abbreviations: *AE = Adverse Event; SAE = Serious Adverse Event; CI = Confidence Interval; and CONSORT = Consolidated Standards for Reporting Trials.*

[a] Make no reference to the appendix

[b] 3 reports made no reference to AE data throughout the article

[c] 5 reports indicate no withdrawals

[d] 6 reports specify the number of withdrawals and reasons but none of the reasons are related to AEs

[e] This includes 3 reports with no AE data (as per footnote [b]), 2 reports that provide generic statements regarding AE data and 1 report that only reported continuous outcomes

[f] This includes 3 reports with no AE data and 2 reports that provide generic statements regarding AE data (as per footnote [e])

[g] 6 papers specifically state that no serious adverse events occurred

Eighty-nine percent of trials reported a subset of all the AEs they collected. How AEs are 'selected' for inclusion in the article was not consistent or clear, and in 3% of studies it was impossible to discern how the authors had selected the AEs they presented for inclusion. Twenty-six percent of reports selected events based on a frequency threshold e.g. events experienced by greater than x% in any group; 9% of reports used a measure of severity to select events e.g. AEs of grade 3 or higher; 23% of reports included events based on seriousness; and 8% included AEs based on relatedness to treatment (percentages are not independent as the majority of reports used several different criteria for selection). Supplementary Tables A5 and A6 provide full details of selection criteria used.

For peer review only - http://bmjopen.bmj.com/site/about/guidelines.xhtml

We found that 41% of trials analysed AEs in participants that received at least one dose, 29% of trials used all randomised participants and 9% did not specify the analysis population (Table 3). Further details on analysis populations used are given in supplementary Table A7.

Nearly 80% of trials reported the number of participants who withdrew from the trial; of these 35% (51 of 146 reports) reported whether the withdrawals were due to AEs and of these 24% (12 of 51 reports) reported the actual events that caused withdrawals. Results presented and analysis performed was predominantly on 'patients with at least 1 event' with 84% of reports providing no information on the number of events occurring. An example of how to incorporate information on number of events is presented in reference [35]. Forty-one percent of trials reported information on the severity of AEs. Five percent of trials include a report of at least one event with duration, but presenting such data is limited in the main report. The trials that did present this information did so in a variety of ways. For example incorporating the information into the AE table with summary statistics such as the mean duration of certain events or presenting it for a subgroup of events in the footnotes of AE tables e.g. "*One event of non-serious squamous cell carcinoma (day 210, resolved on day 215; adalimumab treatment was not interrupted).*"[36-38] Twenty-eight percent of reports included information on the timing of AEs (Table 3).

Serious adverse events were typically well documented (73%) and six reports (3%) explicitly stated that no serious events had occurred. However, for forty-four reports (24%) it was not possible to discern if no serious events had occurred or whether they were simply omitted from the report. Forty-two percent (57 of 134 reports) of reports included details on whether the events had been classified as related to the intervention (Table 3).

For peer review only - http://bmjopen.bmj.com/site/about/guidelines.xhtml

Analysis of AE outcomes

Binary

The majority of trials summarised binary outcomes using frequencies (94%) and percentages (87%). Despite a lack of power to undertake formal hypothesis testing, 47% reported p-values for binary outcomes. For example "*There were no between-group differences in the rate of patients with at least 1 adverse event (16.7% [14 patients] in the clopidogrel group vs 21.8% [19 patients] in the placebo group; difference, −5.2% [95% CI, −17% to 6.6%]; P = .44)*." However, with a total safety population of 171 such a test would have only had 13% power to detect such a difference and was therefore substantially underpowered. The conclusion that "*No significant increase in adverse events was observed*" makes no reference to the 95% confidence interval presented which indicates that the findings were in fact compatible with a 17% decrease in experiencing at least on AE as well as a near 7% increase.[39]

Continuous

There was a pervasive practise (59%) of categorising continuous clinical and laboratory outcomes. Of the trials that did not dichotomise continuous AE data nearly 70% performed some form of statistical significance testing (Table 3). Whilst continuous outcomes do not suffer to the same degree regarding lack of power, multiple testing is still a problem, however no multiplicity corrections for continuous outcomes were performed.

Of the trials that performed statistical significance testing on AE data, only three made an adjustment for multiplicity of tests (all three on dichotomised outcomes).[36, 40, 41] Two of which used a Bonferroni correction and adjusted for the number of pairwise comparisons between each of the

For peer review only - http://bmjopen.bmj.com/site/about/guidelines.xhtml

treatment groups for each individual event rather than the total number of significance tests performed. As such both analyses would have still been effected by issues of multiple testing.

Twelve percent of reports used graphs to illustrate AE data (Table 3). The CONSORT extension highlighted the value of graphs for summarising such data, especially for conveying information on time-to-event outcomes.[42] An example of such a plot is included in the supplement of reference [43] (eFigure2).

We assessed any reference to the CONSORT harm extension and found that none of the included studies mentioned it. Of the four journals included in the review, the Lancet was the only journal that made specific reference to the harm extension in their guidelines to authors.

DISCUSSION

The safety profile of a medicinal product is established through evidence collected from several sources including clinical trials, observational studies and spontaneous reports.[44] The advantage of clinical trial data is that these provide a controlled comparison of the rate of AEs allowing causality to be evaluated but have the disadvantage that the sample size is often not large enough to detect rare ADRs.

To ensure that a useful and comprehensive picture of the safety profile is provided to all relevant parties clear reporting of AEs from clinical trials is required. Recent research has shown the quality of reporting is substandard.[7-16] The aim of this study was to review contemporary practice across four leading medical journals for AE collection, analysis and reporting practices, highlighting any areas for

For peer review only - http://bmjopen.bmj.com/site/about/guidelines.xhtml

improvement and examples of good practice. We found that the collection, reporting and analysis of AE data in clinical trials is inconsistent and RCTs as a source of safety data are underutilised. Analysis of AE was often inappropriate with suboptimal practice including ignoring valuable information on repeated events and inappropriate practice of underpowered multiple hypothesis testing.

Collection and assessment methods

The CONSORT extension to harm was developed with the aim to improve reporting of safety data in RCTs.[42] None of the included studies referenced the CONSTORT harm extension and of the items in our review that are covered in CONSORT many were not well reported.[17] This suggests that the CONSORT extension is not being routinely adopted by authors to aid their reporting. Most journals now request that authors include a completed CONSORT checklist when they submit their article but we are not aware of any journal that request the CONSORT harm extension to also be submitted. Of the four journals in this review the Lancet is the only journal that makes specific reference to the harm extension in their guidelines to authors. The CONSORT statement contains a single item related to safety, item 19: ‘all important harms or unintended effects in each group’ should be reported.[42] This may explain why some items listed on the CONSORT extension for harm were reported by so few trials. The mandatory submission of CONSORT harms by journals may support better reporting.

For peer review only

We found that the method of AE collection was poorly reported. This has important implications for the type and frequency of AEs reported with “passive collection resulting in fewer recorded AEs”.[45, 46] Where the method was given the timing of collection was typically also reported and we would recommend continuation of this practice. The frequency of AE collection has further important implications on the number of events reported. More frequent assessment and longer follow-up will

For peer review only - http://bmjopen.bmj.com/site/about/guidelines.xhtml

result in more AEs reported.[17] It is important to consider these factors when making conclusions about the safety profile.

The method of attribution between drug and AE was another area where reporting practice was inadequate. However, the joint pharmaceutical/journal collaboration indicate that such attribution has 'limited value' given the 'inherent subjectivity in such attribution'.[18]

Prespecified analysis

We found that formal assessments of AEs regarding stopping for emerging ADRs utilising statistical rules was rare. Subjective assessments of overwhelming amounts of data could easily lead to potential signals of harm being missed. There could be benefits to incorporating more objective statistical methods alongside clinical review to assist the evaluation of AE information to help better identify drug harm relationships. Graphical displays have gone some way towards aiding interpretation.[47-51]

Selection of AEs and reporting practices

Due to space constraints in journal reports AE information is often included in the appendix. Whilst we encourage use of appendices and supplementary material for including additional detail on AEs, we caution authors against depositing all AE data into such documents without attempting to present a summary of the AE profile in the main article. It is important that the main report strikes a

For peer review only - http://bmjopen.bmj.com/site/about/guidelines.xhtml

balance between efficacy and harm therefore allowing a risk-benefit assessment to be made solely from the article.

The failure to report any information on AEs restricts interpretation and prevents a risk-benefit assessment. We identified two reports that made generic summaries of the overall safety profile and it was clear in both that there had been harmful effects. However, the authors did not include any further information. Three reports contained no information leaving readers uninformed as to any additional information these studies may provide on the safety profile. Ambiguous reporting prevents building an accurate picture of the safety profile. As such profiles are developed on accumulating evidence, it is important that each study report to the same standard and information is not wasted.

We found that the selection criteria used by authors to decide what AEs to include in the report were arbitrary and inconsistent. This will have important implications when synthesising data across studies to construct safety profiles. Authors would benefit from guidance to facilitate consistency but research in this area is lacking. Lineberry et al. recommended clinically relevant events that should always be reported (deaths, SAEs and events leading to discontinuation of intervention) and criteria that should be considered when deciding what other AEs to report e.g. interest based on the disease(s) under investigation, comorbidities of the study population, intervention mechanism, trial duration.[18] Standard outcomes for a drug class would be one potential solution to avoid issues of inconsistency suggested by Cornelius et al.[11]

CONSORT recommend that AE analyses should be performed on the intention-to-treat (ITT) population to maintain the random assignment.[17] However it is clear from our review that this

For peer review only - http://bmjopen.bmj.com/site/about/guidelines.xhtml

population label is not always appropriately and consistently applied. There is a tendency for studies to make modifications to the ITT population. Using the ITT or modified-ITT population is likely to underestimate the risk by inflating the denominator with participants who may have never received the study drug.[52] Such estimates are appropriate for health economic evaluations where estimates of the cost-effectiveness will inform policy level decisions regarding how to treat the population. However, a more appropriate population for AE analysis to inform prescriber and patient decisions may be those that receive at least one dose. It is important that authors clearly define and specify a suitable safety analysis population and consider how this affects their conclusions.

Proxy outcomes can be used as a measure of the impact of AEs on patients. Examples include the number of withdrawals due to any reason, withdrawals due to AEs, the number of events an individual experiences, the severity of the AE and the duration. A high proportion of trials reported withdrawal for any reason and this is likely to be as a result of the CONSORT recommendations.[42] The other outcomes were not frequently reported and increasing this could facilitate interpretation.[17] This information would permit better evaluation of the impact of AEs and the tolerability of the intervention to inform patients' and clinicians' treatment decisions. Reporting numbers that experience at least one event only and not providing information on repeated events masks valuable information that may be important to the patient and the cost-effectiveness evaluation. For example, chronic, repeated headaches over an extended duration will have an important impact for patients compared to a single headache or headaches over a short duration but it is not possible to distinguish between these two scenarios when reported as 'at least one event'.[18] Severity of events was also an important aspect that was often not differentiated. For example, there would be a different impact on patients' quality-of-life with mild compared to severe nausea, which could lead to changes in dosing regimens. Displaying such information for all AEs in tables would soon become overwhelming and make interpretation difficult. Graphical approaches

For peer review only

For peer review only - http://bmjopen.bmj.com/site/about/guidelines.xhtml

have been suggested as a solution to aid review. Examples of such a plots can be found in reference [53]. Online appendices and supplementary material provide more opportunity to include this important information.

For serious adverse events information on the time of likely onset can be useful information to inform patient monitoring plans. For example, the documented risk of suicide and suicidal ideation within the first few weeks of starting anti-depressant allows patients and prescribers to remain alert and monitor closely for this period. Nearly a third of reports included such information and we would encourage authors to adopt this practice.

Analysis of AE outcomes

The majority of trials in this review included a balanced report of AEs alongside benefit. However many included generic statements regarding the safety profile such as 'the intervention was well tolerated' or 'the intervention exhibited a good safety profile' and these were frequently based on post-hoc statistical tests. Guidelines caution against such tests.[18] The results of which are difficult to interpret as a lack of significance does not indicate that the intervention is safe and conversely multiple testing without adjustment will increase the number of significant differences due to chance.[54, 55]

Graphs are an efficient method to convey and interpret large amounts of data and can make it easier to flag potential safety signals.[50, 51, 53] Twelve percent of studies included in the review used graphs to present AE data and an example of one such report is given in the supplementary eTable of reference [56].

For peer review only - http://bmjopen.bmj.com/site/about/guidelines.xhtml

For peer review only

Recommendations for consideration for immediate adoption by the clinical trial community are summarised in Table 4.

Table 4: Recommendations to improve adverse event analysis and reporting in clinical trial report publications

	Recommendation
**Analysis**	Incorporate objective statistical methods to assist the evaluation of adverse event information.
	Consider avoiding dichotomising continuous data.
	When count outcomes are available (such as repeated events within participants) use appropriate statistical methods.
	Clearly define exposure and specify a suitable safety analysis population.
	Use graphical approaches to help summarise large amounts of data.
**Reporting**	Report adverse event data according to the CONSORT harm checklist.
	Increase the uptake of mandatory submission of CONSORT harm by journals.
	Include a relevant summary of the adverse event profile in the main article. Resist depositing all adverse event data into appendices without summarising.

Limitations of trials

Trials are a valuable source for high quality adverse event data but compared to observational studies have smaller sample size, follow-up periods and generalisability, which restrict the ability to detect rare ADRs, ADRs with long latency and drug interactions in complex populations. The typical duration of a trial means there is often insufficient follow-up to fully characterise the safety profile as it provides limited information on long-term exposure. Stringent inclusion criteria restrict the population the intervention is assessed in and so limited information on drug-interactions is obtained.[5]

For peer review only - http://bmjopen.bmj.com/site/about/guidelines.xhtml

Limitations of this study

Articles included in this review were published in four of the top ranked medical journals therefore results are likely to be biased towards better findings than we would expect if we included all RCTs. Articles are only for year 2015-2016 and as such may not reflect current practice. We also acknowledge that only completing 10% independent check of extracted data would not have removed subjectivity from the data extraction but are happy that ongoing discussion between authors to clarify any queries would have kept this to a minimum. Despite these limitations, this review characterises what those leading the field are doing and provides some examples of good practice that could be adopted.

Conclusions and recommendations for future work

RCTs are a valuable source of information establishing the safety profile of medicinal products. Our review has demonstrated that data is not being fully utilised. Analysis of AE data is frequently inappropriate and RCT reports published over a recent period in high impact general medical journals often provide insufficient and inconsistent information to allow a comprehensive summary of the safety profile to be established.

This research has identified two areas that would benefit from future research. i) Improving the consistency of reporting important AE outcomes across trials to facilitate comparison and synthesis. This is in line with work from the COMET Initiative group (http://www.comet-initiative.org/). The development of CORE safety outcomes by drug class could be considered.[7] ii) Evaluation of methods to analyse AEs in RCTs.

For peer review only - http://bmjopen.bmj.com/site/about/guidelines.xhtml

For peer review only

COMPETING INTERESTS

None declared.

FUNDING

This research was supported by the NIHR grant number DRF-2017-10-131.

DATA SHARING STATEMENT

No additional data are available.

AUTHOR STATEMENT

RP conceived the idea for this review, conducted the search, carried out data extraction and analysis, and wrote the manuscript. VC conceived the idea for the review, performed data extraction, critical revision of the manuscript and supervised the project. LH performed data extraction and critical revision of the manuscript. OS performed critical revision of the manuscript.

For peer review only

For peer review only - http://bmjopen.bmj.com/site/about/guidelines.xhtml

REFERENCES

1. Edwards IR and Biriell C. Harmonisation in pharmacovigilance. *Drug safety* 1994; 10: 93-102. DOI: 10.2165/00002018-199410020-00001.
2. The International Council for Harmonisation of Technical Requirements for Pharmaceuticals for Human Use (ICH). ICH Harmonised Tripartite Guideline. E2A Clinical Safety Data Management: Definitions and Standards for Expedited Reporting. 1994.
3. Ma H, Ke C, Jiang Q, et al. Statistical Considerations on the Evaluation of Imbalances of Adverse Events in Randomized Clinical Trials. *Therapeutic Innovation and Regulatory Science* 2015; 49: 957-965. DOI: http://dx.doi.org/10.1177/2168479015587363.
4. Food and Drug Administration. *Attachment B: Clinical Safety Review of an NDA or BLA of the Good Review Practice. Clinical Reveiw Template (MAPP 6010.3 Rev. 1)*. 2010.
5. Siddiqui O. Statistical methods to analyze adverse events data of randomized clinical trials. *Journal of Biopharmaceutical Statistics* 2009; 19: 889-899. DOI: http://dx.doi.org/10.1080/10543400903105463.
6. Tsang R, Colley L and Lynd LD. Inadequate statistical power to detect clinically significant differences in adverse event rates in randomized controlled trials. *Journal of Clinical Epidemiology* 2009; 62: 609-616.
7. Ioannidis JPA and Contopoulos-Ioannidis DG. Reporting of safety data from randomised trials. *The Lancet* 1998; 352: 1752-1753. DOI: 10.1016/S0140-6736(05)79825-1.
8. Edwards JE, McQuay HJ, Moore RA, et al. Reporting of Adverse Effects in Clinical Trials Should Be Improved. *Journal of Pain and Symptom Management* 1999; 18: 427-437. DOI: 10.1016/S0885-3924(99)00093-7.
9. Ioannidis JA and Lau J. Completeness of safety reporting in randomized trials: An evaluation of 7 medical areas. *JAMA* 2001; 285: 437-443. DOI: 10.1001/jama.285.4.437.
10. Pitrou I, Boutron I, Ahmad N, et al. Reporting of safety results in published reports of randomized controlled trials. *Archives of Internal Medicine* 2009; 169: 1756-1761. DOI: 10.1001/archinternmed.2009.306.
11. Cornelius VR, Sauzet O, Williams JE, et al. Adverse event reporting in randomised controlled trials of neuropathic pain: Considerations for future practice. *PAIN* 2013; 154: 213-220. DOI: 10.1016/j.pain.2012.08.012.
12. Maggi CB, Griebeler IH and Dal Pizzol Tda S. Information on adverse events in randomised clinical trials assessing drug interventions published in four medical journals with high impact factors. *Int J Risk Saf Med* 2014; 26: 9-22. 2014/05/07. DOI: 10.3233/JRS-140609.
13. Smith SM, Wang AT, Katz NP, et al. Adverse event assessment, analysis, and reporting in recent published analgesic clinical trials: ACTTION systematic review and recommendations. *PAIN* 2013; 154: 997-1008. DOI: 10.1016/j.pain.2013.03.003.
14. Peron J, Maillet D, Gan HK, et al. Adherence to CONSORT adverse event reporting guidelines in randomized clinical trials evaluating systemic cancer therapy: a systematic review. *J Clin Oncol* 2013; 31: 3957-3963. 2013/09/26. DOI: 10.1200/JCO.2013.49.3981.
15. Favier R and Crépin S. The reporting of harms in publications on randomized controlled trials funded by the "Programme Hospitalier de Recherche Clinique," a French academic funding scheme. *Clinical Trials* 2018; 0: 1740774518760565. DOI: 10.1177/1740774518760565.
16. Hum SW, Golder S and Shaikh N. Inadequate harms reporting in randomized control trials of antibiotics for pediatric acute otitis media: a systematic review. *Drug Safety* 2018 May 08. DOI: 10.1007/s40264-018-0680-0.
17. Ioannidis JA, Evans SW, Gøtzsche PC, et al. Better reporting of harms in randomized trials: An extension of the consort statement. *Annals of Internal Medicine* 2004; 141: 781-788. DOI: 10.7326/0003-4819-141-10-200411160-00009.

For peer review only

18. Lineberry N, Berlin JA, Mansi B, et al. Recommendations to improve adverse event reporting in clinical trial publications: A joint pharmaceutical industry/journal editor perspective. *BMJ (Online)* 2016; 355: i5078.
19. StataCorp. Stata Statistical Software: Release 15. College Station, TX: StataCorp LLC, 2017.
20. Litonjua AA, Carey VJ, Laranjo N, et al. Effect of Prenatal Supplementation With Vitamin D on Asthma or Recurrent Wheezing in Offspring by Age 3 Years: The VDAART Randomized Clinical Trial. *JAMA* 2016; 315: 362-370. 2016/01/28. DOI: 10.1001/jama.2015.18589.
21. Miller PD, Hattersley G, Riis BJ, et al. Effect of Abaloparatide vs Placebo on New Vertebral Fractures in Postmenopausal Women With Osteoporosis: A Randomized Clinical Trial. *JAMA* 2016; 316: 722-733. 2016/08/18. DOI: 10.1001/jama.2016.11136.
22. Libman IM, Miller KM, DiMeglio LA, et al. Effect of Metformin Added to Insulin on Glycemic Control Among Overweight/Obese Adolescents With Type 1 Diabetes: A Randomized Clinical Trial. *JAMA* 2015; 314: 2241-2250. 2015/12/02. DOI: 10.1001/jama.2015.16174.
23. Writing Committee for the Diabetic Retinopathy Clinical Research Network, Gross JG, Glassman AR, et al. Panretinal Photocoagulation vs Intravitreous Ranibizumab for Proliferative Diabetic Retinopathy: A Randomized Clinical Trial. *JAMA* 2015; 314: 2137-2146. 2015/11/14. DOI: 10.1001/jama.2015.15217.
24. Myles PS, Smith JA, Forbes A, et al. Stopping vs. Continuing Aspirin before Coronary Artery Surgery. *N Engl J Med* 2016; 374: 728-737. 2016/03/05. DOI: 10.1056/NEJMoa1507688.
25. Billings FTt, Hendricks PA, Schildcrout JS, et al. High-Dose Perioperative Atorvastatin and Acute Kidney Injury Following Cardiac Surgery: A Randomized Clinical Trial. *JAMA* 2016; 315: 877-888. 2016/02/26. DOI: 10.1001/jama.2016.0548.
26. Beardsley J, Wolbers M, Kibengo FM, et al. Adjunctive Dexamethasone in HIV-Associated Cryptococcal Meningitis. *N Engl J Med* 2016; 374: 542-554. 2016/02/11. DOI: 10.1056/NEJMoa1509024.
27. Kor DJ, Carter RE, Park PK, et al. Effect of Aspirin on Development of ARDS in At-Risk Patients Presenting to the Emergency Department: The LIPS-A Randomized Clinical Trial. *JAMA* 2016; 315: 2406-2414. 2016/05/18. DOI: 10.1001/jama.2016.6330.
28. Nichol A, French C, Little L, et al. Erythropoietin in traumatic brain injury (EPO-TBI): a double-blind randomised controlled trial. *The Lancet* 2015; 386: 2499-2506. DOI: 10.1016/s0140-6736(15)00386-4.
29. Aitken E, Jackson A, Kearns R, et al. Effect of regional versus local anaesthesia on outcome after arteriovenous fistula creation: a randomised controlled trial. *The Lancet* 2016; 388: 1067-1074. DOI: 10.1016/s0140-6736(16)30948-5.
30. Kaul U, Bangalore S, Seth A, et al. Paclitaxel-Eluting versus Everolimus-Eluting Coronary Stents in Diabetes. *N Engl J Med* 2015; 373: 1709-1719. 2015/10/16. DOI: 10.1056/NEJMoa1510188.
31. Cohen MS, Chen YQ, McCauley M, et al. Antiretroviral Therapy for the Prevention of HIV-1 Transmission. *N Engl J Med* 2016; 375: 830-839. 2016/07/19. DOI: 10.1056/NEJMoa1600693.
32. Isanaka S, Langendorf C, Berthe F, et al. Routine Amoxicillin for Uncomplicated Severe Acute Malnutrition in Children. *N Engl J Med* 2016; 374: 444-453. 2016/02/04. DOI: 10.1056/NEJMoa1507024.
33. Natalucci G, Latal B, Koller B, et al. Effect of Early Prophylactic High-Dose Recombinant Human Erythropoietin in Very Preterm Infants on Neurodevelopmental Outcome at 2 Years: A Randomized Clinical Trial. *JAMA* 2016; 315: 2079-2085. 2016/05/18. DOI: 10.1001/jama.2016.5504.
34. Sheehan WJ, Mauger DT, Paul IM, et al. Acetaminophen versus Ibuprofen in Young Children with Mild Persistent Asthma. *N Engl J Med* 2016; 375: 619-630. 2016/08/18. DOI: 10.1056/NEJMoa1515990.
35. Lind M, Hirsch IB, Tuomilehto J, et al. Liraglutide in people treated for type 2 diabetes with multiple daily insulin injections: randomised clinical trial (MDI Liraglutide trial). *BMJ* 2015; 351: h5364. 2015/10/30. DOI: 10.1136/bmj.h5364.

For peer review only

36. Lincoff AM, Mehran R, Povsic TJ, et al. Effect of the REG1 anticoagulation system versus bivalirudin on outcomes after percutaneous coronary intervention (REGULATE-PCI): a randomised clinical trial. *The Lancet* 2016; 387: 349-356. DOI: 10.1016/s0140-6736(15)00515-2.
37. Ribrag V, Koscielny S, Bosq J, et al. Rituximab and dose-dense chemotherapy for adults with Burkitt's lymphoma: a randomised, controlled, open-label, phase 3 trial. *The Lancet* 2016; 387: 2402-2411. DOI: 10.1016/s0140-6736(15)01317-3.
38. Nguyen QD, Merrill PT, Jaffe GJ, et al. Adalimumab for prevention of uveitic flare in patients with inactive non-infectious uveitis controlled by corticosteroids (VISUAL II): a multicentre, double-masked, randomised, placebo-controlled phase 3 trial. *The Lancet* 2016; 388: 1183-1192. DOI: 10.1016/s0140-6736(16)31339-3.
39. Rodes-Cabau J, Horlick E, Ibrahim R, et al. Effect of Clopidogrel and Aspirin vs Aspirin Alone on Migraine Headaches After Transcatheter Atrial Septal Defect Closure: The CANOA Randomized Clinical Trial. *JAMA* 2015; 314: 2147-2154. 2015/11/10. DOI: 10.1001/jama.2015.13919.
40. Ruperto N, Pistorio A, Oliveira S, et al. Prednisone versus prednisone plus ciclosporin versus prednisone plus methotrexate in new-onset juvenile dermatomyositis: a randomised trial. *The Lancet* 2016; 387: 671-678. DOI: 10.1016/s0140-6736(15)01021-1.
41. Whitehead KJ, Sautter NB, McWilliams JP, et al. Effect of Topical Intranasal Therapy on Epistaxis Frequency in Patients With Hereditary Hemorrhagic Telangiectasia: A Randomized Clinical Trial. *JAMA* 2016; 316: 943-951. 2016/09/07. DOI: 10.1001/jama.2016.11724.
42. Schulz KF, Altman DG, Moher D, et al. Consort 2010 statement: Updated guidelines for reporting parallel group randomized trials. *Annals of Internal Medicine* 2010; 152: 726-732. DOI: 10.7326/0003-4819-152-11-201006010-00232.
43. Lingvay I, Perez Manghi F, Garcia-Hernandez P, et al. Effect of Insulin Glargine Up-titration vs Insulin Degludec/Liraglutide on Glycated Hemoglobin Levels in Patients With Uncontrolled Type 2 Diabetes: The DUAL V Randomized Clinical Trial. *JAMA* 2016; 315: 898-907. 2016/03/05. DOI: 10.1001/jama.2016.1252.
44. Cornelius VR, Liu K, Peacock J, et al. Variation in adverse drug reactions listed in product information for antidepressants and anticonvulsants, between the USA and Europe: a comparison review of paired regulatory documents. *BMJ Open* 2016; 6. DOI: 10.1136/bmjopen-2015-010599.
45. Stephens MD, Talbot JC and Routledge PA. *The Detection of New Adverse Reactions*. 4 ed. London: Macmillan Reference 1998.
46. Bent S, Padula A and Avins AL. Brief communication: Better ways to question patients about adverse medical events: a randomized, controlled trial. *Annals of Internal Medicine* 2006; 144: 257-261. DOI: 10.7326/0003-4819-144-4-200602210-00007.
47. Davis S, Sun H and Jung K. Best practices for reporting safety data to data monitoring committees. *Trials Conference: 4th International Clinical Trials Methodology Conference , ICTMC and the 38th Annual Meeting of the Society for Clinical Trials United Kingdom* 2017; 18.
48. Furey A and Bechhofer R. Effective graphical analyses of adverse events in DMC reports. *Trials Conference: 4th International Clinical Trials Methodology Conference , ICTMC and the 38th Annual Meeting of the Society for Clinical Trials United Kingdom* 2017; 18.
49. Buhr KA, Downs M, Rhorer J, et al. Reports to Independent Data Monitoring Committees: An Appeal for Clarity, Completeness, and Comprehensibility. *Therapeutic Innovation & Regulatory Science* 2017: 2168479017739268. DOI: 10.1177/2168479017739268.
50. Amit O, Heiberger RM and Lane PW. Graphical approaches to the analysis of safety data from clinical trials. *Pharmaceutical Statistics* 2008; 7: 20-35.
51. Zink RC, Wolfinger RD and Mann G. Summarizing the incidence of adverse events using volcano plots and time intervals. *Clinical Trials* 2013; 10: 398-406.
52. Detry MA and Lewis RJ. The intention-to-treat principle: How to assess the true effect of choosing a medical treatment. *JAMA* 2014; 312: 85-86. DOI: 10.1001/jama.2014.7523.

For peer review only

53. Sample Closed Session DMC Report, https://www.biostat.wisc.edu/sites/default/files/Sample_Report_Closed_20160920.pdf (2016, accessed 26/03/2018).
54. Altman DG and Bland JM. Statistics notes: Absence of evidence is not evidence of absence. *BMJ* 1995; 311: 485. DOI: 10.1136/bmj.311.7003.485.
55. Jonville-Béra A, Giraudeau B and Autret-Leca E. Reporting of drug tolerance in randomized clinical trials: When data conflict with authors' conclusions. *Annals of Internal Medicine* 2006; 144: 306-307. DOI: 10.7326/0003-4819-144-4-200602210-00024.
56. Coovadia A, Abrams EJ, Strehlau R, et al. Efavirenz-Based Antiretroviral Therapy Among Nevirapine-Exposed HIV-Infected Children in South Africa: A Randomized Clinical Trial. *JAMA* 2015; 314: 1808-1817. 2015/11/04. DOI: 10.1001/jama.2015.13631.

For peer review only

Supplementary material

Table A1: Data items extracted from publications

			Items collected	Instructions
**Study details**		1	Study number	
		2	Journal	
		3	Funding source: public, private, both or unspecified.	Studies will be assumed to be funded by industry only if this is explicitly stated.
**Study characteristics**		4	Control: placebo, active or both	Select placebo if no active treatment is given, else active. Both should be selected for trials with multiple arms where there is at least one group receiving no active treatment and one group receiving an active treatment.
		5	Number of centres	
		6	Number randomised	
		7	Study duration (length of trial follow-up)	
**Methods**	Details of how AE outcomes were defined (coding, attribution) and were collected (mode of collection, timing)	8	Describe the collection method: passive surveillance, patient prompted, clinical examinations (e.g. vital signs or urine samples), and laboratory tests. (Select all that apply)	*Passive*: If authors state that AEs were collected throughout the study with no further information we will assume that collection was passive.<br>*Prompted*: Prompted methods include, but are not limited to: questions about both specific events and AEs in general, questionnaires, or diaries.
		9	Stated the timing of collection.	
		10	Mention dictionary for coding of events: Researcher defined, MedDRA, CTCAE, WHO-ART, COSTART, ICD-10, other or not applicable	
		11	Describe who undertook the assessment of attribution to study drug: blinded assessor, unblinded assessor or not specified.	
**Planned analysis**	Details of any plans for analysing AE outcomes	12	Describe analysis for AE outcomes in the statistical methods.	Reference must be made to harmful events e.g. AEs or a specific harm event, this cannot be simply how binary events will be analysed.
		13	Define a 'safety' population for analysis.	
		14	Specify a planned interim analysis with stopping criteria: based on efficacy, based on safety, based on both efficacy and safety, yes but no other details given, no planned interim analysis or unclear	Criteria for stopping must be set out, it is not enough to say that the DMC reviewed the data.

For peer review only - http://bmjopen.bmj.com/site/about/guidelines.xhtml

Results	Details of what was reported and where	15	What was reported in the main paper: summaries of type of AEs (e.g. AE, SAE, AR, ADR), actual AE terms, both, neither or not applicable?	Not applicable is relevant when for example authors explicitly state that there are no events or there is only one event so summaries are inappropriate.
		16	What was reported in the appendix: summaries of type of AEs (e.g. AE, SAE, AR, ADR), actual AE terms, both, neither or not applicable?	Not applicable is relevant when for example authors explicitly state that there are no events or there is only one event so summaries are inappropriate.<br><br>We will only search the appendix/supplementary material for AE data if the main article makes reference to it.
		17	Who was the AE analysis performed on: all randomised, participants who took at least a single dose, other or not specified?	
		18	How were number of drop-outs/withdrawals reported: By treatment arm, overall, not reported or not applicable?	Not applicable is relevant when there are no drop-outs/withdrawals.<br><br>This does not include discontinuation of treatment.
		19	Were drop-outs/withdrawals due to AEs reported: Yes, no or not applicable?	Not applicable if drop-outs/withdrawals are not reported or if it is reported that there are no drop-outs/withdrawals.
		20	Were specific AEs causing withdrawals reported: Yes, no or not applicable?	Not applicable if drop-outs/withdrawals due to AEs are not reported or if it is reported that there are no drop-outs/withdrawals due to AEs.
		21	What was the selection criteria for the AEs reported?	Free text response where possibilities can include for example: most frequent, above a severity threshold, SAEs.<br><br>Include details of what's in the main journal article and what is in the appendix separately.
	Details of how AEs were summarised and presented - binary outcomes	22	What summary information was given: Number of people, number of events, both, unclear, not summarised or not applicable?	Only select 'number of events' if presented for each individual event not just overall number of events.<br><br>Not applicable is only relevant when report that there are no AEs.
		23	What analysis was performed: frequencies, percentages, differences and 95% confidence intervals, significance tests, other? (Select all that apply)	

For peer review only - http://bmjopen.bmj.com/site/about/guidelines.xhtml

	Details of how AEs were summarised and presented - continuous outcomes	24	Were continuous outcomes dichotomised: Yes for all, yes for some, no or not applicable?	This includes measures that will have been captured as continuous and then dichotomised for example blood levels, blood pressure etc.
		25	If continuous outcomes were analysed as continuous what analysis was performed: differences in measures of central tendency, significance tests, other? (Select all that apply)	
	Details of how AEs were summarised and presented	26	Were signal detection methods used?	
		27	Were any graphical summaries of AEs presented?	
		28	Were severity ratings given: Yes for all, yes for some, no or not applicable?	
		29	Were numbers of serious events presented: Yes by treatment arm, yes overall, no or not applicable?	If death is reported as part of the efficacy outcome it is not enough to constitute reporting serious events.
		30	Were serious events coded as treatment related: Yes for all, yes for some, no or not applicable?	
		31	Provided information on the duration of events?	This refers to the length of the actual AE i.e. how long did it last.
		32	Provided information on the timing of events?	This refers to the time of onset of the AE.
		33	Accounted for multiplicity of statistical tests?	
		34	Referenced CONSORT extension for harms?	

For peer review only

Table A2: Rationale for items extracted

Item	Rationale
How AE data was collected (mode of collection, timing) and defined (coding, attribution) during the study.	Variation in the collection and definition of events could explain differences in the incidence of observed events.[13, 14] For example specifically asking participants about an event of interest in one treatment group whilst relying on patient report in another is likely to lead to a disparity in incidence of events unlikely to be related to the medicinal product.
Assessment practices of severity of the event or relatedness to the medicinal product.	Attribution of causality by an unblinded assessor allows for subjectivity and bias (even if subconscious) to enter into their decision which can have important implications on the risk-benefit assessment.
Planned AE analysis (final and interim monitoring plans and analysis populations).	For example, the intention-to-treat population is likely to underestimate the AE risk by inflating the denominator. Therefore, this needs to be considered when making conclusions about a drug's safety profile.
How events were selected for inclusion in the journal article.	Due to the space constraints in journal articles it is not always feasible to report all AEs experienced by participants. Therefore, articles often only report a subset of AEs and how these are selected for inclusion has important implications for the safety evaluation. Arbitrary selection criteria can lead to inconsistencies in what is presented across trials for the same disease and/or drug. This prevents an accurate overview of the AEs experienced and invalidates any potential systematic review of events.
How and what summary event information was presented in the journal article.	For example, the number of events and duration of events provides insight into the impact of AEs, with repeated or longer events potentially having far wider clinical implications than a single, shorter event for both patients and prescribers.
How AEs were analysed.	There are many challenges to be considered when analysing AEs in clinical trials. For example, inappropriate statistical testing can lead to misleading conclusions e.g. failure to find a statistically significant result leading authors to conclude that the medicinal product is safe or chance imbalance could lead the authors to erroneously stopping a trial too early.[3-6]

For peer review only

For peer review only - http://bmjopen.bmj.com/site/about/guidelines.xhtml

Table A3: Examples of reporting practice in reviewed articles

Example no.	Study	Example practice	Example
1	Litonjua et al.[20]	Description of AE collection method	*"Study staff met with pregnant women monthly to administer a brief health questionnaire, assess medication use, and monitor for complications (via the questionnaire and medical record review)… After delivery, children were monitored by telephone every 3 months and in-person annually for 3 years, during which time infants' health, respiratory symptoms, and medications were assessed"*
2	Miller et al.[21]	Description of AE collection method	*"Safety evaluations included physical examinations, assessment of vital signs, clinical laboratory tests, and reporting of adverse events at each study visit"*
3	Libman et al.[22]	Description of planned AE analysis	*"The proportions of participants experiencing any adverse event, any related adverse event, any gastrointestinal event, any event other than a gastrointestinal event, at least 1 severe hypoglycaemic event, and at least 1 diabetic ketoacidosis event in each treatment group were compared using the Fisher exact test. The number of adverse events, new adverse events, serious adverse events, and non-serious adverse events were compared between groups using a Wilcoxon rank sumtest."*
4	Gross et al.[23]	Description of planned AE analysis	*"Safety analyses and secondary efficacy analyses used binomial regression, analysis of covariance, or the marginal Cox proportional hazards model as appropriate"*

For peer review only

For peer review only - http://bmjopen.bmj.com/site/about/guidelines.xhtml

Table A4: Stopping criteria for safety

Study	Main article text	Appendix text
Myles et al.[24]	"O'Brien–Fleming stopping boundaries were used to assess efficacy, and <u>*a less stringent boundary was used to assess harm*</u>."	
Billings et al.[25]	"The data and safety monitoring board (DSMB) reviewed patient recruitment practices, safety reporting, and data quality after 30 patients completed the study; performed an interim analysis after 277 patients … had completed the study to assess <u>*safety of the intervention*</u>; and performed a second interim analysis after 546 patients … had completed the study to assess the safety, efficacy, and futility of the intervention. The DSMB made recommendations based on qualitative assessments of the safety, efficacy, and futility of the intervention…"	"<u>*Suspend enrolment*</u> *in any study arm … due to* <u>*safety concerns*</u> *based on study intervention.* <u>*Safety concerns*</u> *include*:<br>• Increase in in-hospital all-cause mortality in subjects randomized to A or B such that the DSMB deems the increase is excessive compared to A or B.<br>• Increased treatment toxicity in either treatment group deemed excessive. Toxicity is defined as moderate or severe myalgias.<br>• Increased severity of adverse events deemed "Probably Related" or "Possibly Related" to study intervention in either treatment group. Itemized adverse event reports separated by treatment will be provided.<br>• Increased AKI incidence in either treatment group deemed excessive. • Increased incidence of stroke or hemodialysis requirement in either group (secondary endpoints) deemed excessive."
Beardsley et al.[26]	"An independent data and safety monitoring committee oversaw trial safety and analyzed unblinded data after every 50 deaths, according to its charter ..."	"The Haybittle-Peto boundary, requiring p<0.001 at interim analysis to consider stopping for efficacy, will be used as guidance. <u>*A level of significance of 1% will be used as a guide for stopping the trial early because of a detected harm of dexamethasone*</u>. In addition, the DMEC will receive conditional power curves to assess whether it remains realistic that the trial will demonstrate superiority of dexamethasone conditional on the data accrued up to the point of the interim analysis. Importantly, the DMEC recommendations will not be based purely on statistical tables but will also use clinical judgment."
Kor et al.[27]	"In addition to statistical criteria for significance, the study included a priori "go-no-go" definitions for recommending continuation to phase 3 study … Briefly, continuation to phase 3 would occur with a positive primary outcome finding along with an acceptable safety profile. An acceptable safety profile was defined as a serious adverse event profile for aspirin that was not statistically worse than placebo (95% CI for the relative risk of any serious adverse event covers the null value of relative risk = 1.0). The "no-go decision" was defined as early termination by the data and safety monitoring board for safety or unfavorable risk/benefit ratio. An indeterminate case in which there was a non–statistically significant effect but this effect was in a clinically meaningful direction was also defined."	Initiate Phase III Study: Demonstrated efficacy signal in addition to adequate safety profile Criteria: Early termination for benefit at interim analysis or p<0.08885 at final analysis (alpha=0.10 for study). <u>*Serious adverse event profile of ASA not statistically worse than placebo (95% confidence interval for the relative risk of any SAE covers the null value of RR=1.0).*</u><br>Further Development Potentially Required: Weak efficacy signal Criteria: Primary endpoint did not achieve a priori level of significance but there were at least a general consistency of secondary endpoints indicating propensity for efficacy with a larger sample size and/or more specific primary endpoint.<br>Abandon Treatment Platform: Harm (in efficacy or safety endpoints) Criteria: Study terminated early per recommendation <u>*by DSMB for safety and/or risk/benefit ratio concerns*</u> (i.e., stop for futility, harm, unacceptable risk profile, etc.)

For peer review only - http://bmjopen.bmj.com/site/about/guidelines.xhtml

Nichol et al.[28]	We used a group sequential statistical approach to do two equally spaced pre-planned interim analyses (at 33% and 67% of total recruitment) to assess accumulated safety data (differential proportions of deep venous thrombosis and total mortality). This ***<u>approach was chosen to provide for early stopping for probable harm</u>*** or strong evidence of benefit. We applied the Haybittle-Peto criterion ($\|Zk\| \geq 3$) for early stopping at these analyses.	

For peer review only

Table A5: Selection criteria used to select AEs presented in the main journal report

Selection criteria	n	%
All AEs presented	20	10.87
AEs in greater than x% in any group	10	5.43
AEs in greater than x% in treatment group	4	2.17
AEs in greater than x% in all patients	1	0.54
Most common (no criteria specified)	9	4.89
Predefined AEs	26	14.13
SAEs	15	8.15
AEs leading to study drug discontinuation/interruption	3	1.63
Treatment related AEs	5	2.72
Grade 3>= events	9	4.89
AEs in greater than x% in any group & predefined/special interest AEs	4	2.17
AEs in greater than x% in any group & frequency between groups differed by more than y% & predefined/special interest AEs	1	0.54
AEs in greater than x% in all patients & predefined/special interest AEs	3	1.63
AEs in greater than x% in treatment group & AEs of special interest	2	1.09
AEs in greater than x% in any group & all SAEs	2	1.09
AEs in greater than x% in all patients & all SAEs	1	0.54
AEs in greater than x% in any group & SAEs related to treatment	1	0.54
Most common (no criteria specified) & predefined/special interest AEs	3	1.63
Most common (no criteria specified) & all SAEs	4	2.17
Most common (no criteria specified) & all SAEs & AEs leading to study drug discontinuation/interruption	1	0.54
Most common (no criteria specified) & treatment related SAEs	1	0.54
AEs where frequency between groups differed by more than y% & all SAEs	1	0.54
AEs of special interest	6	3.26
Grade >=3 AEs in greater than x% of patients	1	0.54
Grade >=3 AEs in greater than x% in intervention & y% in control	1	0.54
Most common (no criteria specified) grade 3>= AEs	1	0.54
Most common SAEs (no criteria specified)	1	0.54
SAEs & AE of special interest	1	0.54
Treatment related AEs in greater than x% of patients	1	0.54
Treatment related AEs in greater than x% in any group	1	0.54
AEs in greater than x% in treatment group & SAEs	1	0.54
AEs in greater than x% in treatment group & SAEs & predefined AEs	2	1.09
AEs in greater than x% in any group & significantly different & SAEs	1	0.54
AEs in greater than x% in any group & treatment related AEs/SAEs	2	1.09
AEs in greater than x% in treatment group & treatment related AEs & SAEs	1	0.54
AEs in greater than x% in treatment group & treatment related AEs in greater than y% in all patients	1	0.54
AEs in greater than x% in any group & Grade 3>= events	1	0.54
AEs in greater than x% in all patients & Grade 3>= events	1	0.54
AEs in greater than x% in all patients & Grade 2>= treatment related AEs	1	0.54
AEs in greater than x% in any group & Grade 3>= events in greater than y% in any group	1	0.54
AEs in greater than x% in any group & SAEs in treatment group	1	0.54
AEs in greater than x% in any group & AEs of special interest & most common (no criteria specified) AEs leading to treatment discontinuation/interruption & predefined AEs	1	0.54
AEs in greater than x% in any group, AEs of special interest in greater than y% in treatment group & treatment related deaths	1	0.54

For peer review only

AEs in greater than x% in treatment group & SAEs in greater than y% in any group	1	0.54
AEs and SAEs occurring more often in treatment group than control	1	0.54
AEs in greater than x% in treatment group & occurred more often in treatment group than control & predefined/special interest AEs	1	0.54
AEs in greater than x% in any group & frequency between groups differed by more than y%, SAEs in greater than z% in any group & all grade >=3 AEs	1	0.54
AEs in greater than x% patients & more than y% difference between treatment groups & AEs leading to treatment discontinuation/interruption & most common SAEs (no criteria specified) & death	1	0.54
Predefined AEs, AEs leading to hospitalisation/death/study drug discontinuation/interruption & SUSARS	2	1.09
Some form of overall summary	6	3.26
Not specified how selected	6	3.26
Not summarised in main paper	11	5.98

For peer review only

Table A6: Selection criteria used to select AEs presented in the appendix

Selection criteria	n	%
All AEs	18	9.78
SAEs	18	9.78
All AEs & SAEs	4	2.17
AEs in greater than x% in any group	7	3.8
AEs in greater than x% in treatment group	2	1.09
AEs in greater than x% in all patients	1	0.54
AEs in greater than x% in any group & all SAEs	2	1.09
AEs in greater than x% in treatment group & all SAEs	1	0.54
AEs in greater than x% in all patients & all SAEs	3	1.63
AEs in greater than x% in treatment group & all SAEs	1	0.54
AEs in greater than x% in treatment group & greater than in control group & all SAEs	1	0.54
SAEs in greater than x% in any group	1	0.54
AEs in greater than x% in any group & SAEs in greater than y% in any group	1	0.54
AEs in greater than x% in any group & AEs of special interest	2	1.09
Treatment related AEs	5	2.72
Treatment related AEs in greater than x% in any group	2	1.09
Grade 3>= events	2	1.09
Predefined AEs	8	4.35
AEs of special interest	1	0.54
AEs leading to study drug discontinuation/interruption	2	1.09
AEs leading to study drug discontinuation & SAEs	1	0.54
Grade 3>= events leading to study drug discontinuation & grade 3>= laboratory results	1	0.54
Treatment related AEs & AEs leading to study drug discontinuation	1	0.54
AEs in greater than x% in all patients leading to treatment discontinuations, SAEs in greater than x% in any group, serious predefined/special interest AEs and clinically significant laboratory results	1	0.54
AEs in greater than x% in any group, treatment related AEs in greater than x% in any group, treatment related SAEs and select AEs	1	0.54
Clinical laboratory data	1	0.54
Predefined AEs, AEs leading to hospitalisation/death/study drug discontinuation/interruption & SUSARS	3	1.63
Deaths	2	1.09
Some form of overall summary	5	2.72
Not specified how selected	2	1.09
Not summarised in the appendix	84	45.65

For peer review only

Table A7: Population used for AE analysis

Analysis population	n	%
Those that took at least a single dose	75	40.76
All randomised	54	29.35
Randomised and not withdrawn/ineligible	19	10.33
Not specified	17	9.24
Not applicable	3	1.63
Took a single dose and underwent AE/toxicity assessment	3	1.63
Active treatment groups	2	1.09
Completed treatment and assessed for primary outcome	2	1.09
Other	2	1.09
Patients who treatment was at least attempted on	1	0.54
Intention-to-treat population	1	0.54
Randomised and assessed for primary outcome	1	0.54
Randomised and attended at least on follow-up visit	1	0.54
Randomised and remained in follow-up	1	0.54
Randomised and underwent AE/toxicity assessment	1	0.54
Randomised, eligible and received at least a single dose	1	0.54

For peer review only

For peer review only - http://bmjopen.bmj.com/site/about/guidelines.xhtml

Reporting checklist for systematic review and meta-analysis.

Based on the PRISMA guidelines.

Instructions to authors

Complete this checklist by entering the page numbers from your manuscript where readers will find each of the items listed below.

Your article may not currently address all the items on the checklist. Please modify your text to include the missing information. If you are certain that an item does not apply, please write "n/a" and provide a short explanation.

Upload your completed checklist as an extra file when you submit to a journal.

In your methods section, say that you used the PRISMA reporting guidelines, and cite them as:

Moher D, Liberati A, Tetzlaff J, Altman DG, The PRISMA Group. Preferred Reporting Items for Systematic Reviews and Meta-Analyses: The PRISMA Statement

		Reporting Item	Page Number
	#1	Identify the report as a systematic review, meta-analysis, or both.	1
Structured summary	#2	Provide a structured summary including, as applicable: background; objectives; data sources; study eligibility criteria, participants, and interventions; study appraisal and synthesis methods; results; limitations; conclusions and implications of key findings; systematic review registration number	2
Rationale	#3	Describe the rationale for the review in the context of what is already known.	5-6
Objectives	#4	Provide an explicit statement of questions being addressed with reference to participants, interventions, comparisons, outcomes, and study design (PICOS).	6

For peer review only

Protocol and registration	#5	Indicate if a review protocol exists, if and where it can be accessed (e.g., Web address) and, if available, provide registration information including the registration number.	n/a - review protocol was not published
rEEligibility criteria	#6	Specify study characteristics (e.g., PICOS, length of follow-up) and report characteristics (e.g., years considered, language, publication status) used as criteria for eligibility, giving rational	6-7
Information sources	#7	Describe all information sources in the search (e.g., databases with dates of coverage, contact with study authors to identify additional studies) and date last searched.	6
Search	#8	Present full electronic search strategy for at least one database, including any limits used, such that it could be repeated.	n/a - we manually searched the electronic contents table of the journals for reports of original RCTs
Study selection	#9	State the process for selecting studies (i.e., for screening, for determining eligibility, for inclusion in the systematic review, and, if applicable, for inclusion in the meta-analysis).	7
Data collection process	#10	Describe the method of data extraction from reports (e.g., piloted forms, independently by two reviewers) and any processes for obtaining and confirming data from investigators.	7
Data items	#11	List and define all variables for which data were sought (e.g., PICOS, funding sources), and any assumptions and simplifications made.	7

For peer review only

Risk of bias in individual studies	#12	Describe methods used for assessing risk of bias in individual studies (including specification of whether this was done at the study or outcome level, or both), and how this information is to be used in any data synthesis.	n/a – the review was to identify current practice, we did not look at and synthesize the actual results of individual studies and as such this assessment was not relevant
Summary measures	#13	State the principal summary measures (e.g., risk ratio, difference in means).	8
Planned methods of analyis	#14	Describe the methods of handling data and combining results of studies, if done, including measures of consistency (e.g., I2) for each meta-analysis.	8
Risk of bias across studies	#15	Specify any assessment of risk of bias that may affect the cumulative evidence (e.g., publication bias, selective reporting within studies).	n/a – please see item #12
Additional analyses	#16	Describe methods of additional analyses (e.g., sensitivity or subgroup analyses, meta-regression), if done, indicating which were pre-specified.	n/a – no such analysis was performed
Study selection	#17	Give numbers of studies screened, assessed for eligibility, and included in the review, with reasons for exclusions at each stage, ideally with a flow diagram.	n/a – manual search resulted in only eligible articles being downloaded
Study characteristics	#18	For each study, present characteristics for which data were extracted (e.g., study size, PICOS, follow-up period) and provide the citation.	9
Risk of bias within studies	#19	Present data on risk of bias of each study and, if available, any outcome-level assessment (see Item 12).	n/a – please see item #12
Results of individual studies	#20	For all outcomes considered (benefits and harms), present, for each study: (a) simple summary data for each intervention group	n/a – only simple descriptive statistics are presented to describe current practice

For peer review only

		and (b) effect estimates and confidence intervals, ideally with a forest plot.	
Synthesis of results	#21	Present the main results of the review. If meta-analyses are done, include for each, confidence intervals and measures of consistency.	9-13
Risk of bias across studies	#22	Present results of any assessment of risk of bias across studies (see Item 15).	n/a – please see item #12
Additional analysis	#23	Give results of additional analyses, if done (e.g., sensitivity or subgroup analyses, meta-regression [see Item 16]).	n/a – please see item #16
Summary of Evidence	#24	Summarize the main findings, including the strength of evidence for each main outcome; consider their relevance to key groups (e.g., health care providers, users, and policy makers	13-21
Limitations	#25	Discuss limitations at study and outcome level (e.g., risk of bias), and at review level (e.g., incomplete retrieval of identified research, reporting bias).	26/27
Conclusions	#26	Provide a general interpretation of the results in the context of other evidence, and implications for future research.	27
Funding	#27	Describe sources of funding or other support (e.g., supply of data) for the systematic review; role of funders for the systematic review.	28

The PRISMA checklist is distributed under the terms of the Creative Commons Attribution License CC-BY. This checklist was completed on 29. May 2018 using http://www.goodreports.org/, a tool made by the EQUATOR Network in collaboration with Penelope.ai

For peer review only
